# Neutralization, effector function and immune imprinting of Omicron variants

**DOI:** 10.1038/s41586-023-06487-6

**Published:** 2023-08-30

**Authors:** Amin Addetia, Luca Piccoli, James Brett Case, Young-Jun Park, Martina Beltramello, Barbara Guarino, Ha Dang, Guilherme Dias de Melo, Dora Pinto, Kaitlin Sprouse, Suzanne M. Scheaffer, Jessica Bassi, Chiara Silacci-Fregni, Francesco Muoio, Marco Dini, Lucia Vincenzetti, Rima Acosta, Daisy Johnson, Sambhavi Subramanian, Christian Saliba, Martina Giurdanella, Gloria Lombardo, Giada Leoni, Katja Culap, Carley McAlister, Anushka Rajesh, Exequiel Dellota, Jiayi Zhou, Nisar Farhat, Dana Bohan, Julia Noack, Alex Chen, Florian A. Lempp, Joel Quispe, Lauriane Kergoat, Florence Larrous, Elisabetta Cameroni, Bradley Whitener, Olivier Giannini, Pietro Cippà, Alessandro Ceschi, Paolo Ferrari, Alessandra Franzetti-Pellanda, Maira Biggiogero, Christian Garzoni, Stephanie Zappi, Luca Bernasconi, Min Jeong Kim, Laura E. Rosen, Gretja Schnell, Nadine Czudnochowski, Fabio Benigni, Nicholas Franko, Jennifer K. Logue, Courtney Yoshiyama, Cameron Stewart, Helen Chu, Hervé Bourhy, Michael A. Schmid, Lisa A. Purcell, Gyorgy Snell, Antonio Lanzavecchia, Michael S. Diamond, Davide Corti, David Veesler

**Affiliations:** 1 Department of Biochemistry, University of Washington, Seattle, WA USA; 2 Humabs BioMed, Bellinzona, Switzerland; 3 Department of Medicine, Washington University School of Medicine, St Louis, MO USA; 4 Vir Biotechnology, San Francisco, CA USA; 5 Institut Pasteur, Université Paris Cité, Lyssavirus Epidemiology and Neuropathology Unit, Paris, France; 6 Faculty of Biomedical Sciences, Università della Svizzera italiana, Lugano, Switzerland; 7 Department of Medicine, Ente Ospedaliero Cantonale, Bellinzona, Switzerland; 8 Division of Nephrology, Ente Ospedaliero Cantonale, Lugano, Switzerland; 9 Clinical Trial Unit, Ente Ospedaliero Cantonale, Lugano, Switzerland; 10 Division of Clinical Pharmacology and Toxicology, Institute of Pharmacological Sciences of Southern Switzerland, Ente Ospedaliero Cantonale, Lugano, Switzerland; 11 Department of Clinical Pharmacology and Toxicology, University Hospital Zurich, Zurich, Switzerland; 12 Clinical School, University of New South Wales, Sydney, New South Wales Australia; 13 Clinical Research Unit, Clinica Luganese Moncucco, Lugano, Switzerland; 14 Clinic of Internal Medicine and Infectious Diseases, Clinica Luganese Moncucco, Lugano, Switzerland; 15 Division of Nephrology, Cantonal Hospital Aarau, Aarau, Switzerland; 16 Institute of Laboratory Medicine, Cantonal Hospital Aarau, Aarau, Switzerland; 17 Division of Allergy and Infectious Diseases, University of Washington, Seattle, WA USA; 18 Department of Pathology and Immunology, Washington University School of Medicine, St Louis, MO USA; 19 Department of Molecular Microbiology, Washington University School of Medicine, St Louis, MO USA; 20 Andrew M. and Jane M. Bursky Center for Human Immunology and Immunotherapy Programs, Washington University School of Medicine, St Louis, MO USA; 21 Center for Vaccines and Immunity to Microbial Pathogens, Washington University School of Medicine, St Louis, MO USA; 22 Howard Hughes Medical Institute, University of Washington, Seattle, WA USA

**Keywords:** RNA vaccines, SARS-CoV-2, Viral infection, Immunological memory, Antibodies

## Abstract

Currently circulating SARS-CoV-2 variants have acquired convergent mutations at hot spots in the receptor-binding domain^1^ (RBD) of the spike protein. The effects of these mutations on viral infection and transmission and the efficacy of vaccines and therapies remains poorly understood. Here we demonstrate that recently emerged BQ.1.1 and XBB.1.5 variants bind host ACE2 with high affinity and promote membrane fusion more efficiently than earlier Omicron variants. Structures of the BQ.1.1, XBB.1 and BN.1 RBDs bound to the fragment antigen-binding region of the S309 antibody (the parent antibody for sotrovimab) and human ACE2 explain the preservation of antibody binding through conformational selection, altered ACE2 recognition and immune evasion. We show that sotrovimab binds avidly to all Omicron variants, promotes Fc-dependent effector functions and protects mice challenged with BQ.1.1 and hamsters challenged with XBB.1.5. Vaccine-elicited human plasma antibodies cross-react with and trigger effector functions against current Omicron variants, despite a reduced neutralizing activity, suggesting a mechanism of protection against disease, exemplified by S309. Cross-reactive RBD-directed human memory B cells remained dominant even after two exposures to Omicron spikes, underscoring the role of persistent immune imprinting.

## Abstract (web summary)

Convergent mutations in hot spots of the spike proteins of currently circulating SARS-CoV-2 Omicron variants increase the binding affinity for the host receptor and promote more efficient fusion with host cell membranes.

## Main

The emergence of the SARS-CoV-2 Omicron (B.1.1.529) variant at the end of 2021 marked a new phase of the COVID-19 pandemic^2^, with lineages harbouring tens of amino acid mutations in their spike (S) glycoprotein leading to enhanced receptor engagement, an altered cell internalization route and unprecedented evasion from neutralizing antibodies^3–6^ (nAbs). As a result, repeated waves of infections driven by successive lineages (such as BA.1/BA.1.1, BA.2 and BA.5) occurred globally, including in individuals who had received multiple COVID-19 vaccine doses.

**Fig. 1:**
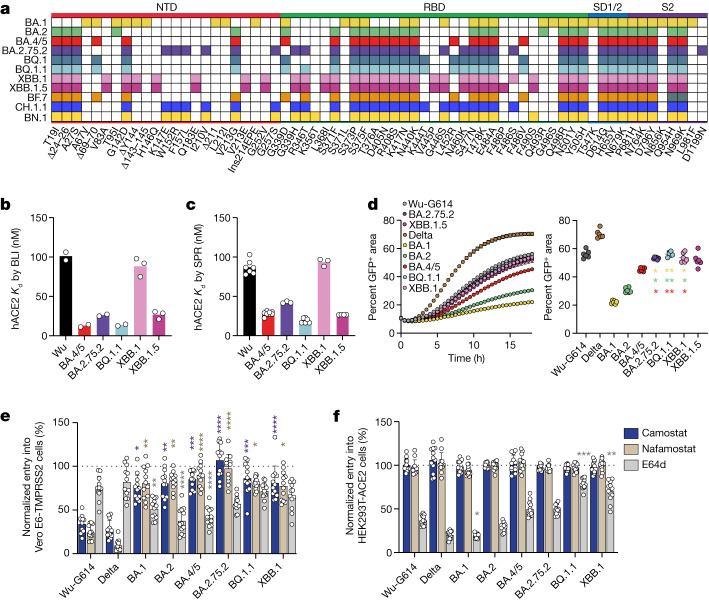
Functional properties of the BQ.1.1, XBB.1, XBB.1.5 and BA.2.75.2 variant S glycoproteins. **a**, Schematic view of S mutations in SARS-CoV-2 variants evaluated in this study. Ins, insertion; SD1/2, subdomains 1 and 2. **b**,**c**, Equilibrium dissociation constants (*K*_d_) measured by BLI (**b**; *n* = 2 or 3 independent experiments) and SPR (**c**) for binding of the monomeric human ACE2 (hACE2) ectodomain to the indicated immobilized variant RBDs. **d**, Left, cell–cell fusion (indicated as the percentage of GFP^+^ area) between cells expressing the indicated variant S glycoproteins and Vero E6-TMPRSS2 cells measured over an 18-h time-course experiment using a split-GFP system. Right, cell–cell fusion at 18 h (mean ± s.e.m.). Data are from six fields of view from a single experiment and representative of results from two biological replicates. Comparisons of fusogenicity mediated by BA.1, BA.2, or BA.4/5 S to BA.2.75.2, BQ.1.1, XBB.1 and XBB.1.5 S were completed using the one-sided Dunnett’s test; colours of asterisks indicate the reference group for the comparison (BA.1, gold; BA.2, green; BA.4/5, red). **e,f**, Relative entry of VSV pseudotyped with the indicated S variant in Vero E6-TMPRSS2 (**e**) or HEK293T-ACE2 (**f**) cells treated with 50 µM camostat, nafamostat or E64d. Normalized entry was calculated on the basis of entry values obtained for Vero E6-TMPRSS2 or HEK293T-ACE2 cells treated with DMSO only for each pseudovirus. Data are mean ± s.d. Twelve technical replicates were performed for each pseudovirus and inhibitor and one experiment representative of two independent biological replicates is shown. Comparison of relative entry values were made between Wu-G614 S VSV pseudovirus and each of the examined SARS-CoV-2 variant S VSV pseudoviruses using the one-sided Dunnett’s test. *P < 0.05; **P < 0.01; ***P < 0.001; ****P < 0.0001.

RBD-directed antibodies account for most of the neutralizing activity against vaccine-matched and mismatched viruses, whereas the N-terminal domain is mostly targeted by variant-specific nAbs^7–10^. Owing to convergent evolution, currently circulating Omicron variant lineages independently acquired identical or similar amino acid mutations at key antigenic sites in the RBD and in the N-terminal domain (NTD), relative to their presumed BA.2 and BA.5 ancestors^1^. The BA.2.75.2 lineage increased in frequency in multiple countries (such as India) and has the RBD mutations D339H, R346T, G446S, N460K, F486S and R493Q relative to BA.2 (Fig. 1a). CH.1.1 emerged in November 2022 and later accounted for around 12% of infections in Europe and carries the K444T and L452R RBD residue mutations relative to BA.2.75.2. BN.1 descended from BA.2.75 and harbours D339H, R346T, K356T, G446S, N460K, F490S and R493Q RBD mutations relative to BA.2. The BN.1 lineage, which accounted for more than half of the SARS-CoV-2 genomes sequenced in South Korea in January 2023, features an additional RBD N-linked glycosylation sequon at position N354 due to the K356T mutation^11^. XBB is a recombinant from BJ.1 and BM.1.1.1 (BA.2.75 sublineage) and addition of the G252V mutation in S yielded XBB.1, which has D339H, R346T, L368I, V445P, G446S, N460K, F486S, F490S and R493Q RBD substitutions relative to BA.2 (Fig. 1a). Furthermore, the XBB.1.5 lineage, which contains a proline at position 486 instead of a serine (F486 in the Wuhan-Hu-1 strain (hereafter referred to as Wu)), had become globally dominant by early March 2023. BQ.1 and BQ.1.1 were dominant in several Western countries and accounted for up to 55% of all sequenced SARS-CoV-2 genomes in the USA in January 2023. BQ.1.1 has R346T, K444T and N460K RBD mutations relative to BA.5 (Fig. 1a). In this Article, we set out to understand how the constellation of S mutations in circulating SARS-CoV-2 variants affects viral functional properties and the available clinical countermeasures, including vaccines and therapeutic antibodies. Furthermore, we investigate humoral and memory immune responses in human cohorts representative of real-world exposures to SARS-CoV-2 and COVID-19 vaccines to study immune imprinting and guide future vaccine design and deployment.

## Properties of BQ.1.1, XBB.1.5 and BA.2.75.2 S

**Extended Data Fig. 1:**
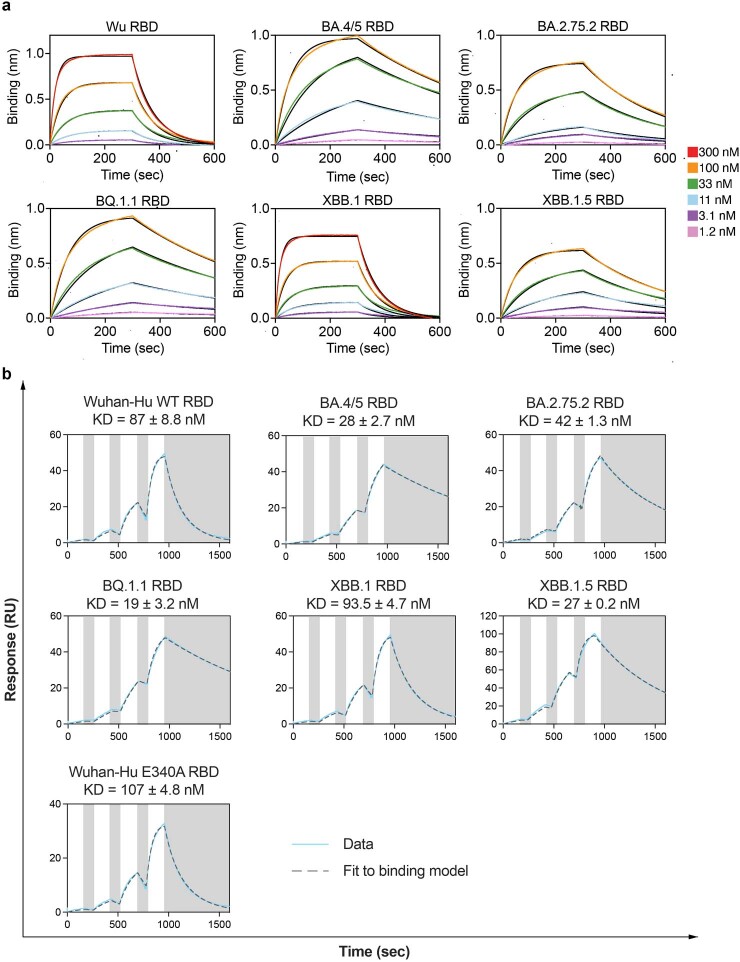
Evaluation of human ACE2 binding to SARS-CoV-2 variant RBDs. **a**, Biolayer interferometry binding curves obtained for monomeric human ACE2 binding to biotinylated Wu, BA.4/5, BA.2.75.2, BQ.1.1, XBB.1 or XBB.1.5 RBDs immobilized at the surface of streptavidin biosensors. Kinetic rate constants and affinities are shown in Supplementary Table 1. Fits are shown as solid black lines. **b**, Sensorgrams of monomeric human ACE2 binding to the Wu, BA.2.75.2, BA.4/5, BQ.1.1, XBB.1, XBB.1.5 and Wu E340A RBDs immobilized at the surface of an SPR chip coated with anti-Avi polyclonal Ab. Experiments were performed with serial dilutions of Fabs and run as single-cycle kinetics. Gray blocks denote the dissociation phase. Fits are shown as dashed grey lines. Kinetic rate constants and affinities are shown in Supplementary Table 2.

We first determined the binding kinetics and affinity of the monomeric human ACE2 ectodomain to immobilized variant RBDs using biolayer interferometry (BLI) (Fig. 1b, Extended Data Fig. 1 and Supplementary Table 1). We measured similar affinities for the BQ.1.1 and BA.5 RBDs (equilibrium dissociation constant (*K*_d_) = 12.8 nM and 13.7 nM, respectively), indicating that the additional BQ.1.1 mutations, which map outside of the ACE2-binding interface, do not influence receptor engagement (Fig. 1b, Extended Data Fig. 1 and Supplementary Table 1). The enhanced ACE2 binding affinity of the BA.2.75.2 RBD (*K*_d_ = 26.2 nM) relative to BA.2, results from the R493Q reversion, as G446S has a negligible effect and F486S has a deleterious effect on ACE2 engagement^12^. ACE2 bound to the XBB.1 RBD with an affinity similar to that of the Wu RBD (*K*_d_ = 88.4 nM and *K*_d_ = 101.1 nM, respectively) whereas it bound more tightly to the XBB.1.5 RBD (*K*_d_ = 26.8 nM), owing to substitution of a serine for a proline at S residue 486 enhancing receptor engagement^12,13^. We observed a similar ranking of these variant RBDs using surface plasmon resonance (SPR) to determine ACE2 binding affinities (Fig. 1c, Extended Data Fig. 1 and Supplementary Table 2). Modulation of ACE2 binding affinities resulted largely from off-rate differences, in agreement with observations made with previous variants^3,5,12,14^. BQ.1.1, BA.2.75.2, XBB.1.5 and BA.5 have similarly high ACE2 binding affinity, suggesting that their viral fitness is not limited by this step of host cell invasion. The markedly higher ACE2 binding affinity of the XBB.1.5 RBD relative to XBB.1 is likely to explain the rapid rise of XBB.1.5 worldwide, as RBD position 486 is the only difference distinguishing these two genomes.

We next compared the kinetics and magnitude of cell–cell fusion promoted by the Wu-G614, Delta, BA.1, BA.2, BA.5, BQ.1.1, BA.2.75.2, XBB.1 and XBB.1.5 S glycoproteins using a split-GFP system^3^. We observed slower and reduced fusogenicity for the BA.5, BA.2 and BA.1 S glycoproteins compared with Wu-G614 and even more so relative to Delta S^15^ (Fig. 1d and Supplementary Fig. 1), in line with previous findings and the lack of syncytia formation observed with authentic viruses^3,16^. BQ.1.1, BA.2.75.2, XBB.1 and XBB.1.5 S, however, promoted membrane fusion more efficiently than the earlier Omicron variants (Fig. 1d and Supplementary Fig. 1), suggesting enhanced fusogenicity, which could augment viral replication kinetics, as described for the Delta variant^17,18^.

BA.1, BA.2 and BA.5 have an altered cell entry pathway relative to previous SARS-CoV-2 variants, and enter preferentially through the endosomal (cathepsin-mediated) route as opposed to the plasma membrane (TMPRSS2-mediated) route^6,16,19,20^. To assess the preferred cell entry route of currently circulating variants, we investigated the effect of protease inhibitors on entry of non-replicative vesicular stomatitis virus (VSV) pseudotyped with S glycoproteins into Vero E6-TMPRSS2 cells (which enables both plasma membrane and endosomal entry routes) and HEK293T-ACE2 cells (which enable endosomal entry only). The serine protease (TMPRSS2) inhibitors camostat and nafamostat potently blocked entry of Wu-G614 and Delta S VSV in Vero E6-TMPRSS2 cells, but had a limited effect on the Omicron variants (Fig. 1e). Reciprocally, the cathepsin B and L inhibitor E64d reduced the entry of BA.1, BA.2 and BA.5 S VSV, whereas there was no significant difference in entry for Delta, BA.2.75.2, BQ.1.1 or XBB.1 S VSV compared with Wu-G614 in Vero E6-TMPRSS2 cells (Fig. 1e). Furthermore, entry mediated by BQ.1.1 and XBB.1 S was reduced by E64d to a lower extent than all other variant S proteins evaluated in HEK293T-ACE2 cells (Fig. 1f). This inefficient use of TMPRSS2 concurs with the identical BQ.1.1, BA.2.75.2 and XBB.1 sequences in the C-terminal part of the S_1_ subunit and the entire S_2_ subunit, which were proposed to mediate the switch in entry route^6,16,19^.

## BQ.1.1, XBB.1 and BN.1 RBD structures

**Fig. 2:**
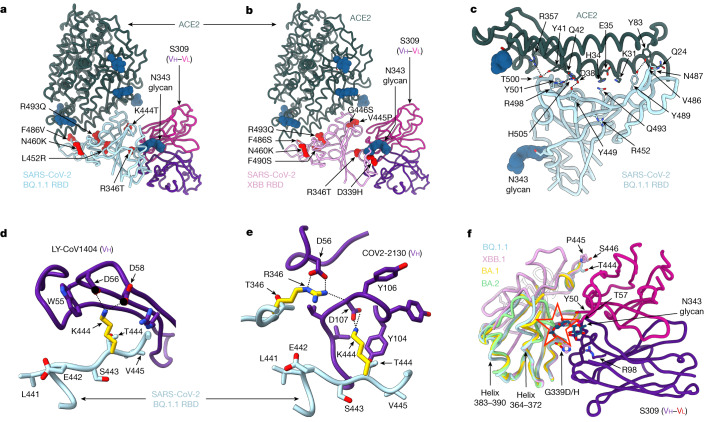
Structural analysis of BQ.1.1 and XBB.1 RBDs. **a**,**b**, Cryo-EM structures of the BQ.1.1 RBD (**a**; cyan) or the XBB.1 RBD (**b**; pink) bound to the human ACE2 ectodomain (green) and the S309 Fab fragment (Vh in purple and Vl in magenta). Amino acid residues mutated relative to Omicron BA.2 are shown as red spheres. **c**, Zoomed-in view of the BQ.1.1 RBD interactions formed with human ACE2 with select amino acid residue side chains shown as sticks. N-linked glycans are shown as dark blue spheres in **a**–**c**. **d**,**e**, RBD-based superimposition of the LY-CoV1404-bound Wu RBD structure (**d**; purple, Protein Data Bank (PDB) ID: 7MMO) or of the COV2-2130-bound Wu RBD structure (**e**; purple, PDB ID: 7L7E) onto the BQ.1.1 RBD cryo-EM structure, highlighting the expected disruptions of electrostatic interactions with the monoclonal antibodies resulting from the K444T and the R346T RBD mutations. **f**, RBD-based superimpositions of the S309-bound BA.1 S (gold, PDB ID: 7TLY), apo BA.2 S (green, PDB ID: 7UB0), S309- and ACE2-bound BQ.1.1 (cyan) and XBB.1 (pink) RBD cryo-EM structures. The N343 glycan along with select side chains are rendered as sticks. The expected N343 glycan clashes with BA.2 residues N370 and F371 (sticks) are indicated with a red star. Residues 368–373 are disordered in the XBB.1 RBD cryo-EM map, as is the case for the adjacent residues 380–392 and were not modelled. Select electrostatic interactions are highlighted with dotted lines in **c**–**e**.

**Extended Data Fig. 2:**
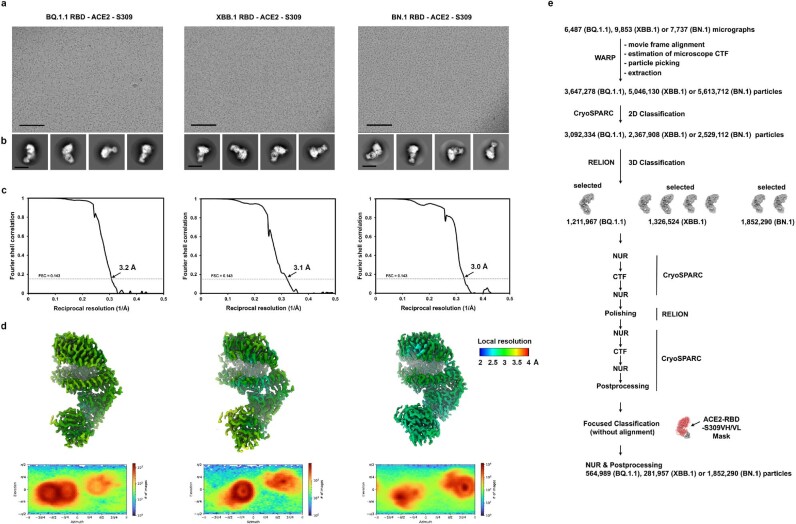
CryoEM data processing of the BQ.1.1, XBB.1 and BN.1 RBDs bound to ACE2 and S309. **a**-**b**, Electron micrographs representative of 6,487, 6,355, or 3,822 micrographs, respectively, (a) and 2D class averages (b) of the BQ.1.1 RBD (left), the XBB.1 RBD (middle) or BN.1 RBD (right) bound to the human ACE2 ectodomain and the S309 Fab fragment embedded in vitreous ice. The scale bar represents 100 nm (a) or 100 Å (b). **c**-**d**, Gold-standard Fourier shell correlation curves (c) and local resolution maps along with angular distribution heat maps calculated using cryoSPARC (d) for the 3D reconstructions of the BQ.1.1 RBD (left), the XBB.1 RBD (middle) or BN.1 RBD (right) bound to the human ACE2 ectodomain and the S309 Fab fragment. The 0.143 cutoff is indicated by a horizontal dashed line. **e**, Data processing flowchart. CTF: contrast transfer function; NUR: non-uniform refinement.

To reveal how amino acid substitutions in the BQ.1.1 and XBB.1 RBDs alter receptor recognition and key antigenic sites, we determined cryo-electron microscopy (cryo-EM) structures for each RBD bound to the human ACE2 ectodomain and to the fragment antigen-binding (Fab) region of the S309 antibody (Fig. 2a,b, Extended Data Fig. 2 and Extended Data Table 1). The R493Q reversion enhances ACE2 binding relative to BA.2^12^, possibly owing to the removal of the positively charged arginine side chain restoring a network of local interactions similar to that made with the Wu RBD^21^ (Fig. 2c and Supplementary Fig. 2a–c). As V445P does not change the conformation of the ACE2-bound XBB.1 RBD in our structure, relative to BQ.1.1, and none of the three residue substitutions relative to BA.2.75.2 involve side-chain-mediated contacts with the host receptor, the V445P mutation might alter the backbone conformational dynamics of the free XBB.1 RBD and possibly dampen ACE2 binding. The BQ.1.1 RBD structure shows that the K444T substitution would abrogate salt bridges with the carboxyl side chains of the LY-CoV1404 (bebtelovimab parent) heavy chain residues D56 and D58 or of the COV2-2130 (cilgavimab parent) heavy chain residue D107 (Fig. 2d,e). Moreover, R346T (present in BQ.1.1 and XBB.1) would abrogate a salt bridge with the COV2-2130 heavy chain residue D56 (Fig. 2e); G446S (present in XBB.1) is expected to reduce COV2-2130 binding sterically^5^ and V445P (XBB.1) probably reduces binding to LY-CoV1404, owing to a loss of van der Waals interactions (Supplementary Fig. 2d). These data explain the markedly reduced binding and neutralization of LY-CoV1404, COV2-2130 and the COV2-2130/COV2-2196 (Evusheld parent) cocktail against the BQ.1.1 and XBB.1 variants^1,12,22^.

**Fig. 3:**
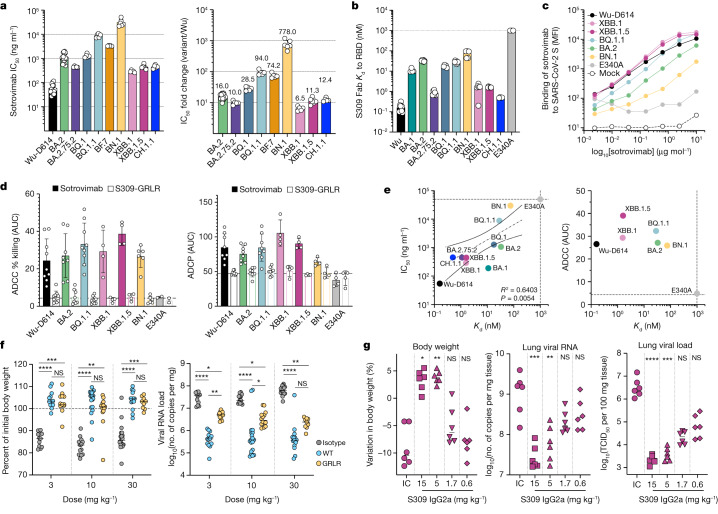
S309-mediated neutralization, effector functions and in vivo protection. **a**, Sotrovimab-mediated neutralization of SARS-CoV-2 variant S VSV pseudoviruses presented as absolute potency (half-maximal inhibitory concentration (IC_50_)) (left) or relative to neutralization of Wu-D614 S VSV (right). Each symbol represents individual biological replicates (*n* = 5–20). **b**, SPR analysis of S309 Fab binding to SARS-CoV-2 RBD variants. Each symbol represents *K*_d_ values from independent experiments (*n* = 3–10). **c**, Binding of sotrovimab immunoglobulin G (IgG) to cell-surface expressed SARS-CoV-2 S variants. **d**, Left, natural killer cell-mediated ADCC in the presence of sotrovimab or S309-GRLR. Data are presented as mean area under the curve (AUC) ± s.d. of percentage killing (*n* = 4–10 donors). Right, ADCP of target cells via CD14^+^ peripheral blood mononuclear cells in the presence of sotrovimab or S309-GRLR. Data are presented as mean AUC ± s.d. (*n* = 4–8 donors). **e**, Correlation of sotrovimab Fab binding affinity (from **b**) with neutralizing activity (from **a**) or ADCC (from **d**). Dotted lines indicate the limit of detection for neutralization and binding affinity or the mean S309-GRLR AUCs for different variants. *R*^2^ and *P* values are derived from two-tailed Pearson correlation. **f**, Body weight loss (left) and lung viral RNA load (right) on day 6 after infection of K18-hACE2 mice receiving S309, S309-GRLR or 30 mg kg^−1^ of an isotype-matched control antibody (anti-WVN^51^) one day before challenge. Solid lines represent the median; dotted lines represent the lower limit of quantification; *n* = 9–20 mice per group. Kruskal–Wallis ANOVA with Dunn’s post-test. **g**, Body weight (left), viral genomic RNA (middle) and replicating viral titres (right) measured in lungs on day 4 after infection of Syrian hamsters receiving S309 hamster IgG2a or 15 mg kg^−1^ of an isotype control (IC) monoclonal antibody (MPE8 IgG2a) one day before challenge. *n* = 6 hamsters per group. Kruskal–Wallis ANOVA with Dunn’s post-test between isotype control and S309. NS, not significant. Source data

The structures demonstrate that S309 binds to both the BQ.1.1 and XBB.1 RBDs and reveal the molecular basis for the accommodation of the H339 residue in the XBB.1 epitope, involving extensive H339 side-chain interactions with S309 heavy chain complementarity-determining region 1 (CDRH1) and CDRH3 (Fig. 2f). The S309 binding pose is indistinguishable from that observed when it is bound to the Wu^23^ or the BA.1^5^ RBD (Fig. 2f). The S371F mutation, which is present in BA.2, BA.5, BQ.1.1, XBB.1, XBB.1.5 and BA.2.75.2, leads to conformational changes of the RBD helix comprising residues 364–372 that are sterically incompatible with the glycan N343 conformation observed in S309-bound S structures^24^. In the S309-BQ.1.1 complex structure, helix 364–372 is weakly resolved and adopts a conformation similar to that observed in the S309-bound BA.1 structure^5^ but distinct from apo BA.2^25^ or apo BA.5 S^26^ structures (Fig. 2f). Residues 368–373 are disordered in the cryo-EM map of the S309–XBB.1 RBD complex—as is the case for the adjacent residues 380–392—and were therefore not modelled (Fig. 2f). These findings underscore the conformational frustration of helix 364–372, which is constrained to adopt an energetically disfavoured conformation for a F371-harbouring mutant owing to S309 binding, which could explain the reduced neutralizing activity of S309 against these variants^1,13,22,27,28^ (Fig. 3a and Supplementary Table 3).

**Extended Data Fig. 3:**
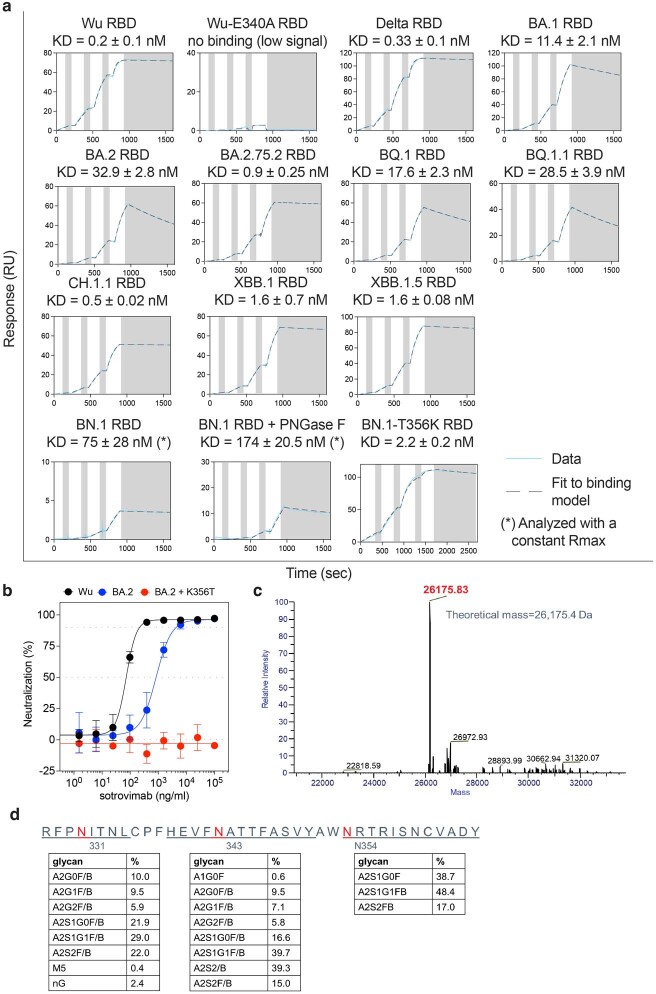
Cross-reactivity of S309 with SARS-CoV-2 variant RBDs. **a**, Representative sensograms of S309 Fab binding to the SARS-CoV-2 Wu, Wu E340A, Delta, BA.1, BA.2, BA.2.75.2, BA.5, BQ.1, BQ.1.1, CH.1.1, XBB.1, XBB.1.5, BN.1 (deglycosylated or not with PNGase F) and BN.1-T356K RBDs immobilized at the surface of a SPR chip coated with anti-Avi polyclonal Ab. Experiments were performed with serial dilutions of Fabs and were run as single-cycle kinetics. Gray blocks denote the dissociation phase. Fits are shown as dashed grey lines. Kinetic rate constants and affinities are shown in Supplementary Table 4. **b**, Sotrovimab-mediated neutralization of Wu-D614, BA.2 and BA.2-K356T S VSV pseudoviruses using VeroE6 as target cells. Dose-response curves of one representative experiment out of 2. **c**, Intact mass-spectrometry analysis of PNGase F-treated BN.1 RBD showing complete removal of N-linked glycans. **d**, Individual glycan profiling of the three glycosylation sites of the BN.1 RBD (N331, N343, N354) by LC-MS peptide map analysis. nG: no glycan detected.

On the basis of the cryo-EM visualization of S309 binding to the BQ.1.1 and XBB.1 RBDs and the fact that sotrovimab remains the only therapeutic antibody with in vitro neutralizing activity against currently circulating variants, we investigated the binding kinetics and affinity of the S309 Fab to the immobilized Wu, Delta, BA.1, BA.2, BA.2.75.2, BQ.1, BQ.1.1, BN.1, XBB.1, XBB.1.5, CH.1.1 and Wu-E340A RBDs using SPR (Fig. 3b and Supplementary Tables 3 and 4). As expected, the E340A escape mutant in the Wu RBD abolished S309 binding^23,29^. The binding affinity of S309 against Omicron variant RBDs decreased up to around 160-fold, primarily owing to faster dissociation rates compared with the Wu RBD. For the BN.1 RBD, however, the S309 Fab exhibited a decrease of around 100-fold in the on-rate compared with that of the Wu RBD, resulting in an approximately 370-fold decrease in affinity (Fig. 3b, Extended Data Fig. 3 and Supplementary Table 4). The K356T mutation is likely to abolish a crucial salt bridge formed with S309 heavy chain variable region (Vh) E108 (as resolved in PDB entry 7TN0^5^), as (1) the BN.1 RBD harbouring the T356K reversion bound S309 similarly to the BA.2.75.2 RBD (the BN.1 RBD is distinguished by K356T, S486F and F490S, with only residue 356 being involved in the epitope); and (2) sotrovimab did not neutralize BA.2 S(K356T) VSV pseudovirus (Extended Data Fig. 3a,b and Supplementary Table 3). Moreover, deglycosylation of the BN.1 RBD with peptide:N-glycosidase F (PNGase) did not improve S309 binding compared with untreated BN.1, despite complete removal of the N354 glycan introduced by the K356T mutation, as confirmed by mass spectrometry (Extended Data Fig. 3c,d and Supplementary Table 4). Finally, we determined a cryo-EM structure of the BN.1 RBD bound to the human ACE2 ectodomain and the S309 Fab (sotrovimab parent) (Extended Data Fig. 2 and Extended Data Table 1) which resolves the *N*-acetylglucosamine linked to N354, which is located near—but does not make contact with—the S309 CDRH3 (Supplementary Fig. 3). Collectively, these results indicate that the loss of the K356(RBD)–E108(S309 Vh) salt bridge is the main mechanism of dampened binding affinity to and neutralization of BN.1 and that the newly introduced N354 glycan has a minimal effect on S309.

**Extended Data Fig. 4:**
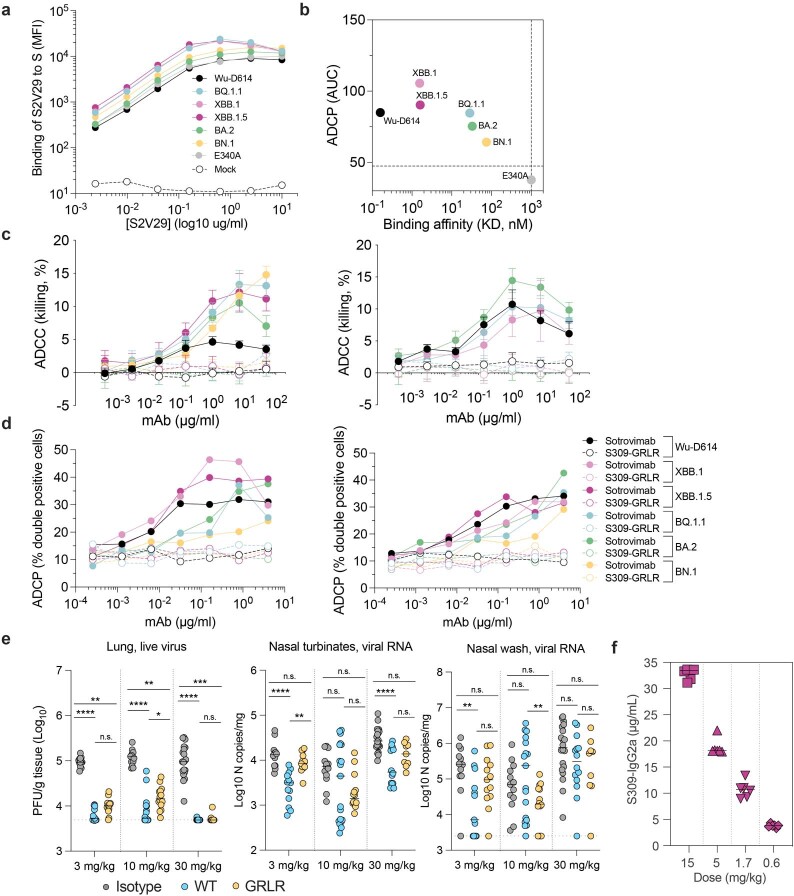
Sotrovimab promotes Fc-mediated effector functions and protects against viral challenge with the SARS-CoV-2 BQ1.1 and XBB.1.5 variants. **a**, Binding of the S2V29 monoclonal Ab to SARS-CoV-2 S variants expressed at the surface of ExpiCHO-S cells as measured by flow cytometry. S2V29 retains potent and equal binding against Wu-D614, BQ.1.1, XBB.1, XBB.1.5, BA.2, BN.1 and BA.2-E340A VSV pseudoviruses and was therefore used for quantifying cell-surface S expression. **b**, Correlation of sotrovimab Fab binding affinity with ADCP. The ADCP AUC values from Fig. 3d are plotted on the y-axis and the binding affinity to each RBD variant obtained in Fig. 3b is plotted on the x-axis. Dotted lines indicate the limit of detection for binding affinity and the mean of S309-GRLR AUCs from the different variants. **c**, ExpiCHO cells transiently transfected with S variants were incubated with the indicated concentrations of sotrovimab or S309-GRLR (G236R/L328R loss-of-function mutations introduced in the Fc domain of the human IgG1 heavy chain) and mixed with NK cells isolated from healthy donors in a range from 6:1 to 9:1 (NK:target cells). Target cell lysis was determined by a lactate dehydrogenase release assay. Data are presented as mean values +/– standard deviations (SD) from duplicates obtained using NK cells from two representative donors, both being homozygous for genotype V/V158 FcγRIIIa. **d**, ExpiCHO cells transiently transfected with S variants were fluorescently labelled with PKH67, incubated with the indicated concentrations of sotrovimab or S309-GRLR mAb and mixed with PBMCs labelled with CellTrace Violet from two healthy donors heterozygous for genotype R/H131 FcγRIIa at a ratio of 20:1 (PBMC:target cells). Association of CD14^+^ monocytes with S-expressing target cells (ADCP) was determined by flow cytometry. **e**, Eight-week-old female K18-hACE2 mice received 3, 10 or 30 mg/kg of S309 (parent of sotrovimab) or S309-GRLR or 30 mg/kg of an isotype-matched control monoclonal Ab (anti-West Nile virus hE16^51^) by intraperitoneal injection one day before intranasal inoculation with 10^4^ FFU of SARS-CoV-2 BQ.1.1. n = 9–20 animals per group. Tissues were collected at six days after infection. Lung live virus titer (left panel) and nasal turbinate (center panel) or nasal wash (right panel) viral RNA determined by RT-qPCR on day 6 are plotted (short, solid lines indicate the median; dotted lines indicate the LLOQ; n = 9–20 mice per group; Kruskal-Wallis ANOVA with Dunn’s post-test; ns, not significant; *P < 0.05, **P < 0.01, ***P < 0.001, **** *P* < 0.0001). **f**, Serum concentration of S309 hamster IgG2a measured by ELISA at day 4 post-infection. n = 6 hamsters per group.The horizontal bar represents the median.

**Extended Data Fig. 5:**
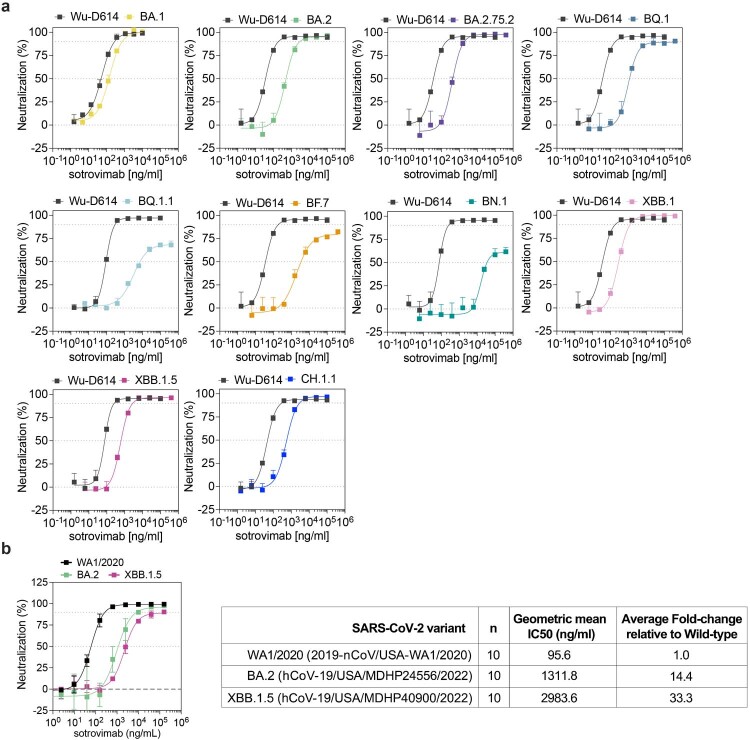
Sotrovimab neutralization of SARS-CoV-2 Omicron variants. **a**, Sotrovimab-mediated neutralization of Wu-D614, BA.2, BA.2.75.2, BQ.1, BQ.1.1, BF.7, XBB.1, BN.1, XBB.1.5. and CH.1.1 S VSV pseudoviruses using VeroE6 as target cells. Dose-response curves displaying the means of triplicates ± SD of one representative experiment out of at least 5 experiments are shown. **b**, Sotrovimab-mediated neutralization of WA1/2020 (2019-nCoV/USA-WA1/2020), Omicron BA.2 (hCoV-19/USA/MDHP24556/2022) and Omicron XBB.1.5 (hCoV-19/USA/MDHP40900/2022) authentic viruses using VeroE6-TMPRSS2 as target cells. Neutralization data (left panel) represent the means of triplicates ± standard deviation from one representative of n = 10 biologically independent experiments. Shown is also the geometric mean IC_50_ and average fold-change relative to wild-type of the 10 performed experiments (right panel).

As illustrated by our cryo-EM structures (Fig. 2f), S309-induced selection of a structurally frustrated backbone conformation around position F371 and of a subset of amino acid side chain rotamers compatible with Fab binding at position 339^5^, along with the extensive interactions formed between the RBD H339 side chain and S309 CDRH1 and CDRH3, probably participate in modulating the distinct affinities observed by SPR. Moreover, the reduction of S309 neutralization potency against BQ.1.1 relative to BQ.1 is probably due to R346T in BQ.1.1, as this mutation abrogates electrostatic interactions with the S309 light chain variable region (Vl) D93. Nevertheless, we found that the sotrovimab IgG cross-reacted with full-length, cell-surface-expressed BQ.1.1, XBB.1 and XBB.1.5 S trimers to a similar or greater degree than those observed for BA.2 S, and to a lesser extent with BN.1 S (Fig. 3c and Extended Data Fig. 4a). These data show that sotrovimab IgG bound avidly to all currently dominant Omicron SARS-CoV-2 variants, although its neutralization potency varied widely, ranging from a 6.5-fold reduction in potency against XBB.1 to a 94-fold reduction against BQ.1.1 and 778-fold reduction against BN.1 relative to Wu (Fig. 3a and Extended Data Fig. 5a,b), in line with recent reports^1,22,27,28,30^.

## S309 protects against BQ.1.1 and XBB.1.5

We next evaluated the ability of sotrovimab to activate antibody-dependent cellular cytotoxicity (ADCC) using primary natural killer effector cells and antibody-dependent cellular phagocytosis (ADCP) with ExpiCHO-S target cells expressing SARS-CoV-2 S of the different Omicron variants at their surface. Sotrovimab efficiently promoted ADCC and ADCP of cells expressing Wu-D614, BA.2, BQ.1.1, XBB.1, XBB.1.5 and BN.1 S in a concentration- and Fc-dependent manner, but did not do so with the BA.2-E340A S (negative control^23,29^) escape mutant or with the S309(G236R/L328R) (hereafter S309-GRLR) Fc mutant, which cannot engage human Fcγ receptors^31^ (Fig. 3d, Extended Data Fig. 4c,d and Supplementary Table 3). Although we observed a linear relationship between the Fab binding affinity and IgG neutralization potency of S309, we found no correlation with the magnitude of ADCC or ADCP, which was similar for all variants despite up to 100-fold differences in their monovalent binding affinities (Fig. 3e and Extended Data Fig. 4b).

To determine the in vivo relevance of these findings, we prophylactically administered S309 (human IgG1) at doses of 3, 10 or 30 mg kg^−1^ to K18-hACE2 transgenic mice (which express human ACE2 under the control of the human *KRT18* promoter) 1 day before challenge with BQ.1.1 (Fig. 3f). S309 administration completely protected mice from weight loss and reduced lung viral RNA loads and infectious virus titres at all doses compared with animals receiving a control antibody (Fig. 3f and Extended Data Fig. 4e), consistent with recent reports on BQ.1.1-challenged hamsters^32^ and non-human primates^33^. To investigate the role of effector functions in protection, we evaluated the in vivo efficacy of S309-GRLR, which is unable to engage human or mouse Fcγ receptors^31^. Our data reveal that although effector functions are not necessary for S309-mediated protection from weight loss, they participate in reducing lung viral RNA burden at 3 and 10 mg kg^−1^ doses (Fig. 3f). Moreover, prophylactic administration of 5 or 15 mg kg^−1^ S309 (hamster IgG2a) to Syrian hamsters challenged with XBB.1.5 reduced weight loss and viral burden (Fig. 3g), with a similar effect on body weight loss to that observed against Delta^24^, highlighting a disconnect between in vivo efficacy and in vitro neutralization potency. Collectively, these data demonstrate that S309 protects animals from challenge with two of the most immune-evasive circulating SARS-CoV-2 variants and that the elicited effector functions contribute to this activity at low antibody doses in a prophylactic setting.

## Bivalent vaccines elicit cross-nAbs

Vaccination represents a main line of defence against SARS-CoV-2, and recent mRNA vaccine updates have led to the administration of bivalent formulations. To assess the effects of the constellation of S mutations in the currently dominant variants on vaccine-elicited antibody responses, we quantified plasma neutralizing activity using VSV pseudotyped with Wu-G614, BA.1, BA.5, BQ.1.1, XBB.1, XBB.1.5 or BA.2.75.2 S. We compared plasma from individuals obtained 15–30 days after vaccination or PCR-confirmed breakthrough infection in 8 cohorts: (i) vaccinated 4 times with the Wu monovalent S mRNA vaccine, with no known infection (Wu_4_ vaccinated); (ii) vaccinated 3 times with Wu monovalent S mRNA vaccine and then 1 time with Wu/BA.5 bivalent S mRNA vaccine, with no known infection (Wu/BA.5 bivalent vaccinated); (iii) infected in 2020 and subsequently vaccinated 3 to 4 times with Wu monovalent S mRNA vaccine and then 1 time with Wu/BA.5 bivalent S mRNA vaccine (pre-Omicron infected–Wu/BA.5 bivalent vaccinated); and (iv) vaccinated with Wu monovalent S mRNA vaccine before experiencing a breakthrough infection with Omicron BA.1, BA.2, BA.2.12.1 or BA.5, followed by a vaccination with the Wu/BA.5 bivalent S mRNA vaccine (Omicron BT–Wu/BA.5 bivalent vaccinated). We also studied 4 additional cohorts from Switzerland, where a Wu/BA.1 bivalent S mRNA booster was available: (v) vaccinated 3 times with Wu monovalent S mRNA vaccine, with no known infection (Wu_3_ mono); (vi) vaccinated 3 times with Wu monovalent S mRNA vaccine after pre-Omicron infection (pre-Omicron–Wu_3_ mono); (vii) vaccinated 3 times with Wu monovalent S mRNA vaccine and then 1 time with Wu/BA.1 bivalent S mRNA vaccine, with no known infection (Wu/BA.1 biv); and (viii) vaccinated 3 times with Wu monovalent S mRNA vaccine and then 1 time with Wu/BA.1 bivalent S mRNA vaccine, with a BA.1 or a BA.2 breakthrough infection (Omicron BT–Wu/BA.1 biv).

**Fig. 4:**
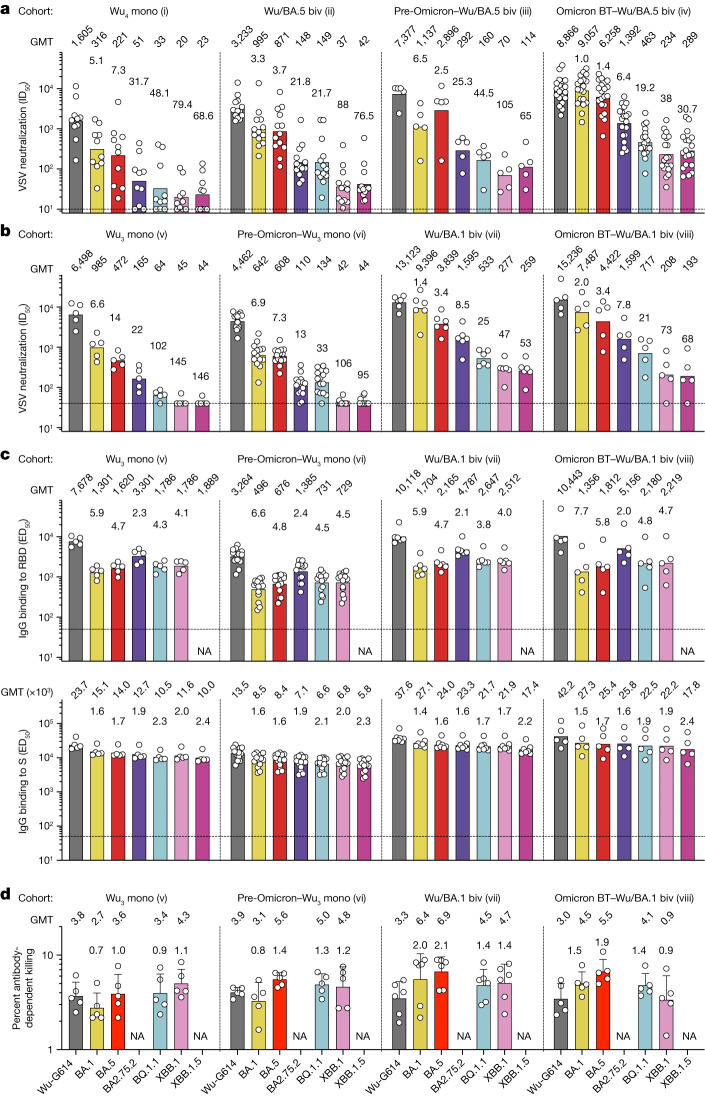
Neutralization, binding and Fc-dependent effector functions of vaccine- and infection-elicited antibodies against emerging Omicron variants. **a**,**b**, Neutralization of VSV pseudotyped with the indicated SARS-CoV-2 variant S by plasma samples from cohorts i–iv (**a**) and cohorts v–viii (**b**). Plasma neutralizing titres are expressed as half-maximal inhibitory dilution (ID_50_) values from *n* = 2 biological (**a**) and technical (**b**) replicates. Bars and values above graphs represent geometric mean titre (GMT). The fold loss of neutralization against each Omicron variant compared with Wu-G614 is shown above each bar. Horizontal dashed lines indicate the limit of detection (In **a**, ID_50_ = 10; in **b**, ID_50_ = 40). Cohorts: (i) vaccinated 4 times with the Wu monovalent S mRNA vaccine, with no known infection (Wu_4_ mono); (ii) vaccinated 3 times with Wu monovalent S mRNA vaccine and then 1 time with Wu/BA.5 bivalent S mRNA vaccine, with no known infection (Wu/BA.5 biv); (iii) infected in 2020 and subsequently vaccinated 3 to 4 times with Wu monovalent S mRNA vaccine and then 1 time with Wu/BA.5 bivalent S mRNA vaccine (pre-Omicron–Wu/BA.5 biv); (iv) vaccinated with Wu monovalent S mRNA vaccine before experiencing a breakthrough infection with Omicron BA.1, BA.2, BA.2.12.1 or BA.5, followed by a vaccination with the Wu/BA.5 bivalent S mRNA vaccine (Omicron BT–Wu/BA.5 biv); (v) vaccinated 3 times with Wu monovalent S mRNA vaccine, with no known infection (Wu_3_ mono); (vi) vaccinated 3 times with Wu monovalent S mRNA vaccine after pre-Omicron infection (pre-Omicron–Wu_3_ mono); (vii) vaccinated 3 times with Wu monovalent S mRNA vaccine and then 1 time with Wu/BA.1 bivalent S mRNA vaccine, with no known infection (Wu/BA.1 biv); and (viii) vaccinated 3 times with Wu monovalent S mRNA vaccine and then 1 time with Wu/BA.1 bivalent S mRNA vaccine, with a BA.1 or a BA.2 breakthrough infection (Omicron BT–Wu/BA.1 biv). **c**, Binding of plasma IgGs to SARS-CoV-2 RBDs and S trimers from indicated variants as measured by ELISA. Bars and values above the graphs represent GMT from *n* = 2 technical replicates. The fold change of binding titre to the Omicron variant compared with Wu is shown above each bar. Horizontal dashed lines indicate the cut-off in the assay based on binding to uncoated plates (median effective dose (ED_50_) = 50). **d**, ADCC as measured by natural killer cell-mediated cell lysis of ExpiCHO-S cells transiently transfected with Wu-D614, BA.5, BQ.1.1 or XBB.1 S and incubated with plasma samples. The percentage of cell lysis is shown for each donor plasma sample diluted 1/200 from cohorts v–viii (*n* = 5 donors for cohort v, *n* = 5 for cohort vi, *n* = 6 for cohort vii and *n* = 5 for cohort viii). Bars and values above the graphs represent GMT. Error bars show s.d. The fold change of activation with Omicron variants compared with Wu-G614 is shown above each bar. NA, not assayed. Demographics are summarized in Supplementary Table 5. Statistically significant differences of mean neutralization and binding titres within and between cohorts are shown in Supplementary Table 7. Samples from cohorts i–iv were obtained in Seattle, USA; samples from cohorts v–viii were obtained from Ticino, Switzerland.

**Extended Data Fig. 6:**
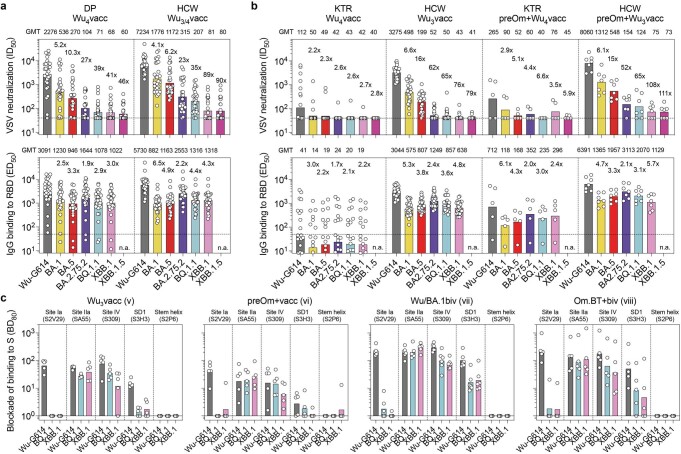
Neutralization, binding and fine specificity of vaccine- and infection-elicited plasma Abs against emerging Omicron variants in dialysis patients, kidney transplant recipients and healthy individuals. **a**,**b**, Neutralization of SARS-CoV-2 pseudotyped VSV carrying Wu-G614, BA.1, BA.5, BA.2.75.2, BQ.1.1, XBB.1 and XBB.1.5 (upper panels) by plasma Abs and binding to matched RBDs by plasma IgGs from dialysis patients (DP) (a) or kidney transplant recipients (KTR) (b) after receiving 4 (Wu_4_vacc) doses. Samples are compared to those from healthcare workers (HCW) collected 2–4 weeks (a) or 2–4 months (b) after receiving 3 or 4 doses of monovalent Wu vaccine. Shown are ID_50_ values from n = 2 technical replicates. Bars and values on top represent geometric mean ID_50_ titers (GMT). Fold-loss of neutralization against Omicron variants as compared to Wu-G614 is shown above each corresponding bar. Horizontal dashed lines indicate the limit of detection in the neutralization assay (ID_50_ = 40) and the cut-off in the ELISA assay based on binding to uncoated plates (ED_50_ = 50). Cohort demographics are summarized in Supplementary Table 6. Statistically significant differences of mean neutralization and binding titers within and between cohorts are shown in Supplementary Table 8. **c**, Competition ELISA (blockade of binding) between individual S site-specific monoclonal Abs and plasma from vaccinated individuals (cohorts v-viii). S2V29 binds to the RBM. Each plot shows the magnitude of inhibition of binding to immobilized Wu-G614, BQ.1.1 and XBB.1 S in the presence of each monoclonal Ab, expressed as reciprocal plasma dilution blocking 80% of the maximum binding response (BD_80_). Points represent the BD_80_ measured for each individual plasma donor as determined from n = 1 experiment and bars represent geometric mean BD_80_ titers.

Vaccination with Wu/BA.5 or Wu/BA.1 bivalent S mRNA vaccine elicited similar nAb titres against Wu-G614 S pseudovirus to those observed in matched cohorts vaccinated against Wu S but increased nAb titres against BA.1 S and BA.5 S pseudoviruses (Fig. 4a,b and Supplementary Figs. 4 and 5). Moreover, bivalent vaccination elicited detectable neutralizing activity against vaccine-mismatched XBB.1, XBB.1.5, BA.2.75.2 and BQ.1.1 S pseudoviruses, irrespective of prior infection status, whereas little to no neutralization of these variants was detected after vaccination with monovalent Wu S mRNA vaccine (Fig. 4a,b). Moreover, plasma neutralizing activity against currently circulating Omicron variants after four doses of monovalent Wu S vaccine was low for patients on maintenance dialysis and undetectable against any variants for immunosuppressed individuals following kidney transplantation, underscoring the difficulties associated with protecting these at-risk populations (Extended Data Fig. 6a,b). Overall, these data suggest that bivalent Wu/BA.1 and Wu/BA.5 mRNA vaccines elicit more potent and broader antibody responses against vaccine-matched and mismatched Omicron variants than the monovalent Wu S mRNA vaccine.

## Plasma antibodies promote effector functions

**Extended Data Fig. 7:**
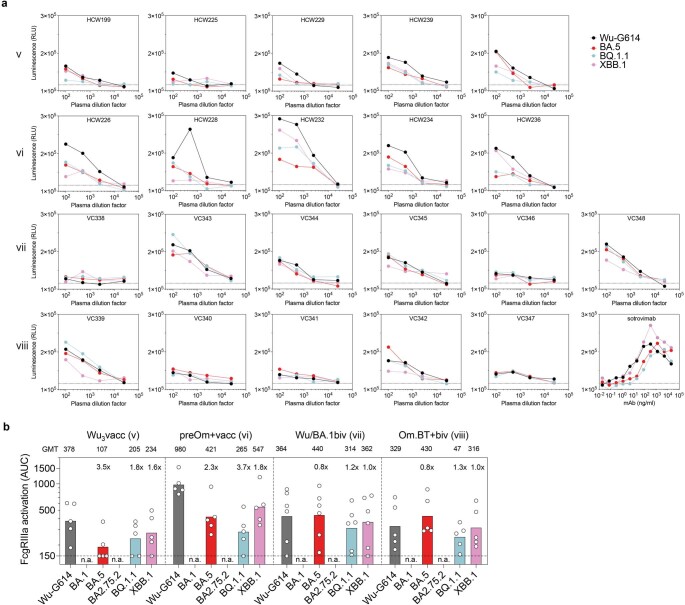
Activation of FcγRIIIa by individual plasma samples. **a** Activation of high-affinity (V158) FcγRIIIa measured using Jurkat reporter cells and SARS-CoV-2 Wu-G614, BA.5, BQ.1.1 and XBB.1 S-expressing ExpiCHO as target cells. Luminescence (RLU) values from one experiment are shown with plasma samples from cohorts v-viii (n = 5 donors for cohort v, n = 5 for cohort vi, n = 6 for cohort vii and n = 5 for cohort viii) and compared to sotrovimab. Horizontal dotted line indicates the lowest limit of detectable activation (RLU = 115,737). **b**, AUC values from one experiment. Bars and values on top represent geometric mean AUC titers (GMT). Fold-change of activation with Omicron variants as compared to Wu-G614 is shown above each corresponding bar. Horizontal dashed line indicates the lowest limit of detectable activation (AUC = 150). n.a., not assayed.

On the basis of the findings that Fc-mediated effector functions contribute to S309-mediated protection in a mouse model of BQ.1.1 infection, we assessed binding to RBD and S as well as ADCC mediated by plasma antibodies from cohorts v to viii. The marked reduction of nAb titres against currently dominant Omicron variants was not paralleled by a similar decrease in IgG binding titres to matched RBDs or S trimers (Fig. 4c). The lack of plasma antibodies competing for binding of an RBD site Ia (class 1) monoclonal antibody to BQ.1.1 and XBB.1 S (Extended Data Fig. 6c) suggests that the reduction of neutralizing activity against these variants is driven by dampened cross-reactivity of receptor-binding motif (RBM)-directed antibodies, a correlate of neutralization potency^7^. This is consistent with the notion that SARS-CoV-2 is evolving primarily to escape nAb responses^1^. Whereas binding titres remained equivalent against all Omicron variants for healthy individuals and patients undergoing dialysis, the strong immunosuppression of kidney transplant recipients was associated with limited or no detectable binding and neutralizing plasma antibodies (Extended Data Fig. 6a,b). Plasma antibodies retained the ability to promote cell lysis mediated by natural killer cells (ADCC) and activation of Fcγ receptor IIIa (FcγRIIIa) (V158 allele) against BA.5, BQ.1.1 and XBB.1 S variants expressed at the surface of ExpiCHO target cells (Fig. 4d and Extended Data Fig. 7). Integrating these findings with the in vivo data for S309 presented above suggests that antibodies triggering Fc-mediated effector functions are broadly reactive with Omicron variants and contribute to protection against COVID-19.

## Cross-reactive MBC dominance

**Fig. 5:**
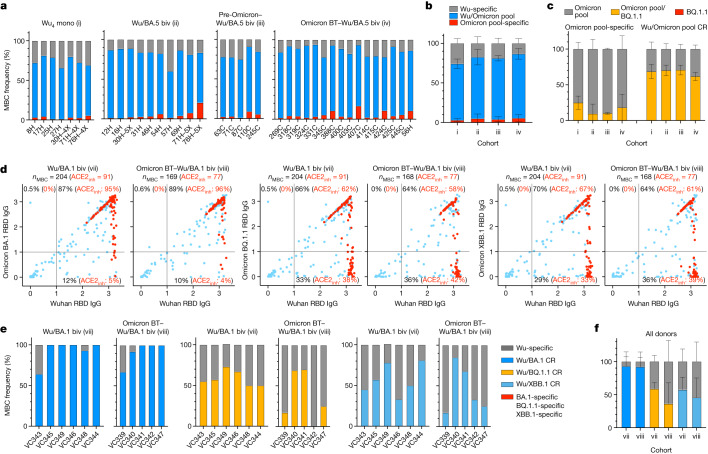
Cross-reactivity of vaccine- and infection-elicited SARS-CoV-2 RBD-binding MBCs. **a**,**b**, Frequency of Wu RBD-binding (grey), Omicron (BA.1, BA.2 and BA.5) RBD pool-binding (red) and cross-reactive (blue) MBCs from donors of cohorts i–iv, as measured by flow cytometry. Data are individual frequencies for each donor (**a**) and mean frequency ± s.d. for each cohort (*n* = 4–16 donors) (**b**). **c**, Analysis of cross-reactivity with the BQ.1.1 RBD of Omicron (BA.1, BA.2 and BA.5) RBD pool-binding (red bars in **b**) and Wu/Omicron (BA.1, BA.2 and BA.5) RBD pool-cross-reactive (CR) (blue bars in **b**) MBCs. Data are mean frequency ± s.d. for each cohort (*n* = 4–16 donors). **d**, Cumulative cross-reactivity with the Wu RBD and the Omicron BA.1, BQ.1.1 or XBB.1 RBDs of IgGs secreted from in vitro-stimulated MBCs, as measured by ELISA. Data are mean absorbance values with the blank subtracted from *n* = 2 replicates of MBC cultures analysed from donors in cohorts vii and viii approximately 3 months after receiving their last vaccine dose. RBD-directed IgGs inhibiting binding of ACE2 to the Wu RBD are depicted in red. The total number (*n*_MBC_) and the number of ACE2-inhibiting (ACE2_inh_) RBD-directed IgG-positive cultures are indicated on top of each graph. Percentages of total (black) and ACE2-inhibiting (red) Wu-binding, Omicron-binding and Wu/Omicron-cross-reactive IgG-positive cultures are indicated within each quadrant. **e**,**f**, Individual frequencies (**e**) and mean (± s.d.) frequencies for each cohort (*n* = 5–6 donors) (**f**) of Wu RBD-specific, Omicron-specific and RBD cross-reactive (BA.1, BQ.1.1 and XBB.1) IgG-positive cultures from donors of cohorts vii and viii.

**Extended Data Fig. 8:**
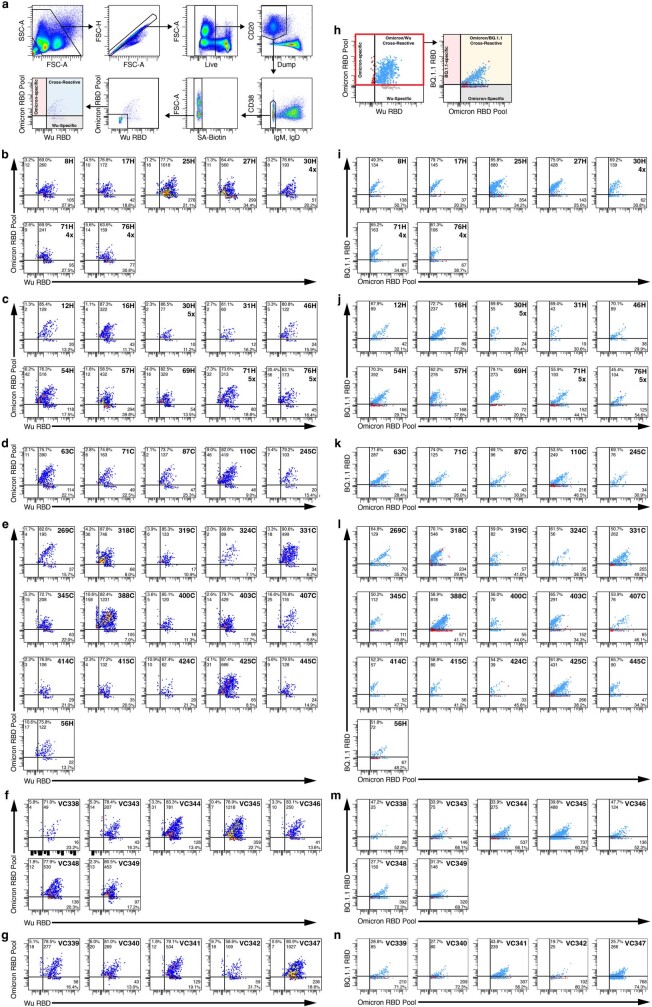
MBC analysis by flow cytometry. **a**, Gating strategy to identify Omicron (BA.1/BA.2/BA.5) RBD pool- and Wu RBD-recognizing MBCs. Dump includes markers for CD3, CD8, CD14, and CD16. Gating for RBD-positive memory B cells was based on staining of PBMCs from healthy donors collected in 2019 prior to the COVID-19 pandemic. Individual plots showing Omicron (BA.1/BA.2/BA.5) RBD pool- and Wu RBD-positive MBCs for Wu_4_ vaccinated (b), Wu/BA.5 bivalent vaccinated (c), pre-Omicron infected-Wu/BA.5 bivalent vaccinated (d), Omicron BT-Wu/BA.5 bivalent vaccinated (e), Wu/BA.1 bivalent vaccinated (f), and Omicron BT-Wu/BA.1 bivalent vaccinated individuals (g). **h**, Gating strategy to determine whether Omicron (BA.1, BA.2, and BA.4/5) RBD pool-positive MBCs recognize the BQ.1.1 RBD. Individual plots showing Omicron (BA.1, BA.2, and BA.4/5) RBD pool and BQ.1.1 RBD-recognizing memory B cells for Wu4 vaccinated (i), Wu/BA.5 bivalent vaccinated (j), pre-Omicron infected-Wu/BA.5 bivalent vaccinated (k), Omicron BT-Wu/BA.5 bivalent vaccinated (l), Wu/BA.1 bivalent vaccinated (m), and Omicron BT-Wu/BA.1 bivalent vaccinated individuals (n). Proportion and counts of memory B cells recognizing one or more RBD(s) is presented for each individual.

We next compared memory B cell (MBC) populations in the Wu_4_ vaccinated and the Wu/BA.5 bivalent vaccinated cohorts (which includes one individual (31H) who received the Janssen adenovirus-vectored (Ad26.COV2.S) COVID-19 vaccine rather than the Moderna (mRNA-1273) or Pfizer (BNT162b2) mRNA COVID-19 vaccines as their primary vaccine series), pre-Omicron infected–Wu/BA.5 bivalent vaccinated, and Omicron BT–Wu/BA.5 bivalent vaccinated subjects (cohorts i–iv) by measuring the frequency of Wu RBD-binding, pooled Omicron (BA.1, BA.2 and BA.5) RBD-binding, and both Wu and pooled Omicron (BA.1, BA.2 and BA.5) RBD-binding cross-reactive MBCs by flow cytometry. Individuals who were exposed to Wu S only (Wu_4_ vaccinated) exhibited the highest frequency of Wu RBD-binding MBCs (mean frequency: 25.8%) and the lowest frequency of cross-reactive MBCs (mean frequency: 71.1%) of the four cohorts (Fig. 5a,b and Extended Data Fig. 8). Individuals who were exposed only once to Omicron S through vaccination had few Omicron RBD-specific MBCs (mean frequency: 4.7%), regardless of whether they had experienced a pre-Omicron SARS-CoV-2 infection (Wu/BA.5 bivalent vaccinated and pre-Omicron infected–Wu/BA.5 bivalent vaccinated cohorts). Most RBD-binding MBCs in these cohorts cross-reacted with the Wu RBD and the pooled Omicron (BA.1, BA.2 and BA.5) RBDs, with uninfected individuals having similar frequencies of cross-reactive MBCs (mean: 77.5%) to individuals who had experienced a pre-Omicron infection (mean frequency: 77.1%) (Fig. 5a,b and Extended Data Fig. 8). These data are consistent with previous analyses of MBC populations in individuals vaccinated against Wu who had experienced an Omicron breakthrough infection, and suggest that immune imprinting limits the development of new Omicron-specific MBCs, although there is efficient recall of cross-reactive MBCs after a single exposure to Omicron S^1,24,34^. Although Omicron BT–Wu/BA.5 bivalent vaccinated subjects had two exposures to Omicron S (one through infection and one through vaccination), they had few Omicron-specific  MBCs (mean: 5.6%), similar to individuals who received only the bivalent booster. MBCs cross-reactive with the Wu RBD and the Omicron (BA.1, BA.2 and BA.5) RBD pool were further enriched (mean frequency: 81.1%) in this cohort compared with the cohort vaccinated with Wu/BA.5 bivalent vaccine without Omicron BT infection (Fig. 5a,b and Extended Data Fig. 8).

**Extended Data Fig. 9:**
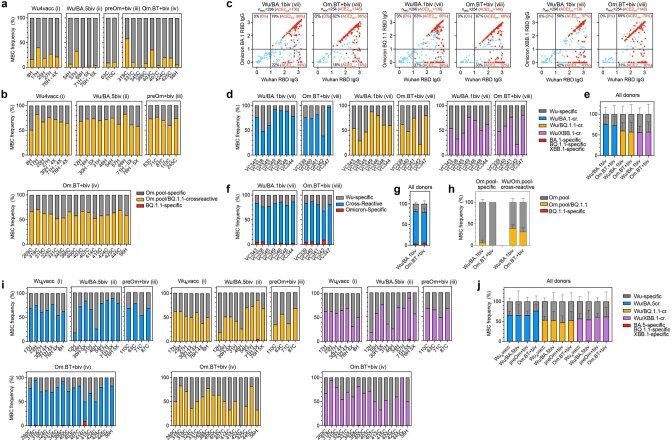
Subanalysis of cross-reactivity of vaccine- and infection-elicited MBCs. **a**,**b**, Analysis of cross-reactivity with the BQ.1.1 RBD of Omicron (BA.1/BA.2/BA.5) RBD pool-specific (a) and Wu/Omicron (BA.1/BA.2/BA.5) RBD pool-cross-reactive (b) MBCs. Om.pool: MBCs reactive with the Omicron (BA.1/BA.2/BA.5) RBD pool in cohorts i–iv. **c**, Cumulative cross-reactivity with the Wu RBD and the Omicron BA.1, BQ.1.1 or XBB.1 RBDs of IgGs secreted from in vitro stimulated MBCs as measured by ELISA. Data represent average OD values with blank subtracted from n = 2 replicates of MBC cultures analyzed from donors of cohorts vii and viii at about 14 days after receiving the last vaccine dose. RBD-directed IgGs inhibiting binding of ACE2 to the Wu RBD are depicted in red. Number of total and ACE2-inhibiting (ACE2_inh_) RBD-directed IgG positive cultures are indicated on top of each graph. Percentages of Wu-specific, Omicron-specific and Wu/Omicron-cross-reactive IgG positive cultures are indicated within each quadrant. **d**,**e**, Individual frequencies (d) and mean frequencies ± SD for each cohort (n = 5-6) (e) of Wu RBD-specific (grey), Omicron-specific (red) and RBD cross-reactive (blue for BA.1, yellow for BQ.1.1 and purple for XBB.1) IgG positive cultures from donors of cohorts vii and viii at about 14 days after receiving the last vaccine dose. **f**,**g**, Frequency of Wu RBD-specific (grey), Omicron (BA.1/BA.2/BA.5) RBD pool-specific (red) and cross-reactive (blue) MBCs from donors of cohorts vii-viii at about 14 days after receiving the last vaccine dose, as measured by flow cytometry. Individual frequencies are shown in panels f and and mean frequencies ± SD for each cohort (n = 4–16) are shown in g. **h**, Analysis of cross-reactivity with the BQ.1.1 RBD of Omicron (BA.1/BA.2/BA.5) RBD pool-specific (red bars of panel g) and Wu/Omicron (BA.1/BA.2/BA.5) RBD pool-cross-reactive (blue bars of panel g) MBCs. Om.pool, MBCs recognizing the Omicron (BA.1/BA.2/BA.5) RBD pool. **M**ean frequencies ± SD are presented for each cohort (n = 5-6). **i**,**j**, Frequency of IgGs specific for the Wu RBD (grey), cross-reactive with the Wu/BA.5 RBDs (blue), the Wu/BQ.1.1 RBDs (orange), the Wu/XBB.1 RBDs (purple) or specific for either the BA.5, BQ.1.1 or XBB.1 RBD (red) as measured by ELISA after in vitro stimulation of MBCs from cohorts i–iv. Individual frequencies and mean ± SD (n = 4–16) are shown in panels i and j, respectively.

We then assessed whether MBCs recognizing the Omicron (BA.1, BA.2 and BA.5) RBD pool could bind the BQ.1.1 RBD (Fig. 5c and Extended Data Fig. 9a,b). Most MBCs that were cross-reactive to Wu and Omicron (BA.1, BA.2 and BA.5) RBDs also recognized the BQ.1.1 RBD (mean frequency: 66.3%), whereas a smaller fraction of MBCs that bound to the Omicron (BA.1, BA.2 and BA.5) RBD pool but not the Wu RBD also recognized the BQ.1.1 RBD (mean frequency: 16.9%), regardless of infection and vaccination status. These Omicron (BA.1, BA.2 and BA.5) RBD pool-specific MBCs were probably elicited de novo upon exposure to Omicron S (through infection and/or vaccination) and their breadth towards currently dominant Omicron variants may increase over time through affinity maturation, similar to observations made after infection with Wu or the WA-1 variant, or after monovalent Wu vaccination^35–40^.

We next determined whether the bivalent boosters formulated against BA.5 or BA.1 differentially affected the composition of the RBD-binding MBC population (Fig. 5d–f and Extended Data Fig. 9c–e). We assessed the cross-reactivity of IgGs secreted by in vitro-stimulated MBCs to the Wu, BA.1, BQ.1.1 and XBB.1 RBDs 2–4 weeks and 3 months following bivalent Wu/BA.1 S vaccination of uninfected individuals and individuals who had experienced an Omicron breakthrough infection (cohorts vii and viii). We did not detect Omicron RBD-specific MBCs after bivalent Wu/BA.1 S vaccination in uninfected or Omicron breakthrough cohorts. Most RBD-binding IgGs, including those that inhibited binding of ACE2 to the Wu RBD, were cross-reactive with the BA.1 RBD, regardless of infection status (mean frequencies at 3 months: 87% in uninfected individuals and 89% in Omicron breakthrough cohorts), whereas a smaller fraction cross-reacted with the BQ.1.1 (66% and 64%, respectively) and XBB.1 (70% and 64%, respectively) RBDs. Consistent with the loss of plasma antibodies cross-reacting with the BQ.1.1 and XBB.1.1 RBMs (Extended Data Fig. 6c), we observed a low frequency of MBC-derived IgGs that blocked binding of BQ.1.1 and XBB.1.1 RBDs to ACE2, most of which were cross-reactive with the Wu RBD (Supplementary Fig. 6). Analysis of cohorts vii and viii by flow cytometry and of cohorts i–iv by in vitro stimulation of MBCs confirmed that there was limited de novo elicitation of MBCs in these individuals (Extended Data Figs. 8 and 9f–j).

Thus, two exposures to Omicron S were not sufficient to overcome the immune imprinting induced by repeated exposures to Wu S, but instead mostly enriched for MBCs cross-reacting with multiple RBD variants. These results concur with the broader plasma nAb responses that we observed upon bivalent mRNA vaccination compared with monovalent Wu mRNA vaccination.

## Discussion

Here we report that recently emerged SARS-CoV-2 Omicron variants show unprecedented immune evasion, reducing nAb titres up to ~150-fold for XBB.1 and XBB.1.5. BQ.1.1, XBB.1.5 and BA.2.75.2 retain high ACE2 binding affinity, similar to earlier Omicron variants, whereas XBB.1 has a lower affinity, similar to that of the Wu RBD. Although XBB.1 and XBB.1.5 are the most immune-evasive of these Omicron variants, the reduced affinity of XBB.1 for ACE2 relative to other co-circulating strains may have hindered its spread. The enhanced ACE2 binding affinity of the more recently emerged XBB.1.5 variant, which harbours the S486P RBD mutation (relative to XBB.1), may explain the current rapid spread of this variant^30^. Our findings illustrate the interplay of immune evasion, fusogenicity and ACE2 binding affinity driving SARS-CoV-2 evolution.

ADCC and ADCP are Fc-mediated effector functions that can promote virus clearance and enhance adaptive immune responses in vivo, independently of direct viral neutralization. Indeed, both neutralizing and binding antibody titres were reported as correlates of protection in a phase 3 clinical study^41^. Sotrovimab retains in vitro effector functions against BA.2 and conferred Fc-dependent protection in the lungs of mice infected with BA.2^42^, in line with the low rate of hospitalization and death during the BA.2 and BA.5 waves in patients treated with sotrovimab^43–45^. Here we show that sotrovimab triggered in vitro effector functions against all Omicron variants assessed at levels similar to that observed with Wu. Prophylactic administration of S309 (the sotrovimab parent antibody) protected mice against BQ.1.1 challenge with a contribution of effector functions, and protected hamsters against XBB.1.5 challenge, despite a reduced in vitro neutralizing activity against these variants. Our observation that vaccine-elicited polyclonal plasma antibodies cross-reacted and promoted ADCC upon recognition of the BA.1, BA.5, BQ.1.1 and XBB.1 S glycoproteins concur with observations made with previous Omicron variants^46,47^ and further hint at a protective role for broadly reactive antibodies with effector functions. Our findings suggest that the erosion of nAb titres is associated with an increased frequency of breakthrough infections, and the persistence of cross-reactive antibodies mediating effector functions may contribute to protection against severe COVID-19.

Immune imprinting—also known as ‘original antigenic sin’—describes how the first exposure to a virus shapes the immunological outcome of subsequent exposures to antigenically related strains. For instance, antibodies secreted by plasmablasts obtained one to two weeks after infection with the antigenically shifted H1N1 influenza virus (formerly known as swine flu) that caused the 2009 flu pandemic were recalled from pre-existing, cross-reactive MBCs^48,49^, whereas plasma cells obtained after the subsequent antigenic exposure (through vaccination) were subtype-specific (that is, targeting non-conserved epitopes). Similarly, Omicron breakthrough infections of Wu-vaccinated subjects primarily recall cross-reactive MBCs specific for epitopes shared by multiple SARS-CoV-2 variants rather than priming naive B cells that recognize Omicron RBD-specific epitopes^1,24,34^. We observed an unexpectedly small number of MBCs specific for Omicron RBDs (and not cross-reacting with the Wu RBD) even after two exposures to Omicron S antigens, including after Wu/BA.5 or Wu/BA.1 bivalent mRNA vaccination. This may be owing to strong immune imprinting resulting from repeated Wu-like S exposures, and possible antigenic dominance of the Wu S antigen in bivalent vaccines^50^. However, relative to monovalent Wu mRNA vaccination, bivalent Wu/BA.5 mRNA vaccination results in enrichment for MBCs that are cross-reactive with vaccine-matched and mismatched RBD variants.

## Methods

### Cells and viruses

Cell lines used in this study were obtained from ATCC (HEK293T and Vero E6), Thermo Fisher Scientific (ExpiCHO-S cells, FreeStyle 293-F cells and Expi293F cells), Takara (Lenti-X 293T cells), a gift from J. Bloom (HEK293T-ACE2)^52^, or generated in-house (Vero E6-TMPRSS2, BHK-21-GFP_1–10_ and Vero E6-TMPRSS2-GFP_11_)^3,53^. None of the cell lines used were authenticated or tested for mycoplasma contamination. SARS-CoV-2 isolates used in this study were obtained through BEI Resources, NIAID, NIH: (hCoV-19/USA-WA1/2020, NR-52281 deposited by the Centers for Disease Control and Prevention ; Lineage B.1.1.529, BA.2; Omicron Variant Isolate hCoV-19/USA/CO-CDPHE-2102544747/2021, NR-56520; Lineage XBB.1.5; Omicron Variant Isolate hCoV-19/USA/MD-HP40900/2022, NR-59104, contributed by A. S. Pekosz). Viruses were propagated and titrated on Vero E6-TMPRSS2 cells in house. The genomic sequences of all strains were confirmed by Sanger and/or next generation sequencing.

### Human donors

Samples from cohorts v–viii along with those from patients undergoing dialysis (DP) kidney transplant recipients (KTR) and healthcare workers (HCW) were obtained from SARS-CoV-2 convalescent and vaccinated individuals under study protocols approved by the local institutional review boards (Canton Ticino and Canton Aargau Ethics Committees, Switzerland). PBMCs for effector function experiments were collected from healthy human donors under the informed consent and authorization of the Comitato Etico of Canton Ticino (Switzerland). All donors provided written informed consent for the use of blood and blood derivatives (such as peripheral blood mononuclear cells, sera or plasma) for research. Sera and PBMCs from cohorts i–iv were obtained from the HAARVI study approved by the University of Washington Human Subjects Division Institutional Review Board (STUDY00000959). Demographic data for these individuals is presented in Supplementary Tables 5 and 6.

### Constructs

The full-length Wu/G614, Delta, BA.1, BA.2, and BA.4/5 S constructs with a 21-amino-acid C-terminal deletion used for pseudovirus assays were previously described elsewhere^3,54^. The full-length BA.2.75.2 and XBB.1 S constructs containing a 21-amino-acid C-terminal deletion were codon optimized, synthesized, and inserted the HDM vector by Genscript. The full-length BQ.1.1 S construct containing a 21-amino acid C-terminal deletion was generated by mutagenesis of the BA.4/5 S construct and the full-length XBB.1.5 containing a 21-amino-acid C-terminal deletion was generated by mutagenesis of the XBB.1 S construct by Genscript.

S expression plasmids used for the generation of VSV pseudoviruses harbour the following mutations. BA.1: A67V, Δ69-70, T95I, G142D, Δ143–145, Δ211, L212I, ins214EPE, G339D, S371L, S373P, S375F, K417N, N440K, G446S, S477N, T478K, E484A, Q493R, G496S, Q498R, N501Y, Y505H, T547K, D614G, H655Y, N679K, P681H, N764K, D796Y, N856K, Q954H, N969K, L981F; BA.2: T19I, L24-, P25-, P26-, A27S, G142D, V213G, G339D, S371L, S373P, S375F, D405N, R408S, K417N, N440K, S477N, T478K, E484A, Q493R, Q498R, N501Y, Y505H, D614G, H655Y, N679K, P681H, N764K, D796Y, N856K, Q954H, N969K; K417N, N440K, G446S, N460K, S477N, T478K, E484A, Q498R, N501Y, Y505H, D614G, H655Y, N679K, P681H, N764K, D796Y, Q954H, N969K; BA.2.75.2: T19I, L24-, P25-, P26-, A27S, G142D, K147E, W152R, F157L, I210V, V213G, G257S, G339H, R346T, S371F, S373P, S375F, T376A, D405N, R408S, K417N, N440K, G446S, N460K, S477N, T478K, E484A, F486S, Q498R, N501Y, Y505H, D614G, H655Y, N679K, P681H, N764K, D796Y, Q954H, N969K, D1199N; BQ.1: T19I, L24-, P25-, P26-, A27S, Δ69-70, G142D, V213G, G339D, S371F, S373P, S375F, T376A, D405N, R408S, K417N, N440K, K444T, L452R, N460K, S477N, T478K, E484A, F486V, Q498R, N501Y, Y505H, D614G, H655Y, N679K, P681H, N764K, D796Y, Q954H, N969K; BQ.1.1: T19I, L24-, P25-, P26-, A27S, Δ69-70, G142D, V213G, G339D, R436T, S371F, S373P, S375F, T376A, D405N, R408S, K417N, N440K, K444T, L452R, N460K, S477N, T478K, E484A, F486V, Q498R, N501Y, Y505H, D614G, H655Y, N679K, P681H, N764K, D796Y, Q954H, N969K; BF.7: T19I, L24-, P25-, P26-, A27S, Δ69-70, G142D, V213G, G339D, R436T, S371F, S373P, S375F, T376A, D405N, R408S, K417N, N440K, L452R, S477N, T478K, E484A, F486V, Q498R, N501Y, Y505H, D614G, H655Y, N679K, P681H, N764K, D796Y, Q954H, N969K; XBB.1: T19I, L24-, P25-, P26-, A27S, V83A, G142D, Y144-, H146Q, Q183E, V213E, G252V, G339H, R346T, L368I, S371F, S373P, S375F, T376A, D405N, R408S, K417N, N440K, V445P, G446S, N460K, S477N, T478K, E484A, F486S, F490S, Q498R, N501Y, Y505H, D614G, H655Y, N679K, P681H, N764K, D796Y, Q954H, N969K; XBB.1.5: T19I, L24-, P25-, P26-, A27S, V83A, G142D, Y144-, H146Q, Q183E, V213E, G252V, G339H, R346T, L368I, S371F, S373P, S375F, T376A, D405N, R408S, K417N, N440K, V445P, G446S, N460K, S477N, T478K, E484A, F486P, F490S, Q498R, N501Y, Y505H, D614G, H655Y, N679K, P681H, N764K, D796Y, Q954H, N969K; CH.1.1: T19I, del24–26, A27S, G142D, K147E, W152R, F157L, I210V, V213G, G257S, G339H, R346T, S371F, S373P, S375F, T376A, D405N, R408S, K417N, N440K, K444T, G446S, L452R, N460K, S477N, T478K, E484A, F486S, Q498R, N501Y, Y505H, D614G, H655Y, N679K, P681H, N764K, D796Y, Q954H, N969K; BN.1: T19I, del24–26, A27S, G142D, K147E, W152R, F157L, I210V, V213G, G257S, G339H, R346T, K356T, S371F, S373P, S375F, T376A, D405N, R408S, K417N, N440K, G446S, N460K, S477N, T478K, E484A, F490S, Q498R, N501Y, Y505H, D614G, H655Y, N679K, P681H, N764K, D796Y, Q954H, N969K.

For BLI and cryo-EM, the SARS-CoV-2 Wu RBD construct containing an N-terminal mu-phosphatase secretion signal and a C-terminal octa-histidine tag followed by flexible linker and Avi tag was previously described elsewhere^55^. The BA.4/5 RBD construct containing an N-terminal BM40 secretion tag and a C-terminal octa-histidine tag followed by flexible linker and Avi tag was previously described elsewhere^3^. The BA.2.75.2, BQ.1.1, XBB.1, and XBB.1.5 RBD constructs containing an N-terminal BM40 secretion tag and a C-terminal octa-histidine tag followed by flexible linker and Avi tag were codon optimized, synthesized, and inserted into the pcDNA3.1(+) vector by Genscript. The boundaries of the construct are N-328RFPN331 and 528KKST531-C. The monomeric human ACE2 ectodomain (residues 19–615) construct used for BLI contains an N-terminal signal peptide and a 10x His tag and was synthesized and inserted into pTwist-CMV by Twist Bioscience.

For SPR, SARS-CoV-2 RBD plasmids encoding residues 328–531 of the S protein from GenBank NC_045512.2 with an N-terminal signal peptide and a C-terminal 8×His–Avi Tag or thrombin cleavage site–8×His–Avi tag. The ACE2 construct used for SPR and cryo-EM, encodes for residues 19–615 from Uniprot entry Q9BYF1 with a C-terminal thrombin cleavage site–TwinStrep–10×His–GGG tag, and N-terminal signal peptide.

### Generation of VSV pseudoviruses

Replication-defective VSV pseudovirus expressing SARS-CoV-2 Wu and variant S were generated as previously described^4^ with some modifications to evaluate cohorts v–viii, DP, KTP and HCW, and monoclonal antibodies. Lenti-X 293T cells (Takara) were seeded in 15-cm^2^ dishes at a density of 10 × 10^6^ cells per dish and the following day were transfected with 25 µg of S expression plasmid with TransIT-Lenti (Mirus, 6600) according to the manufacturer’s instructions. One day after transfection, cells were infected with VSV-luc (VSV-G) with a multiplicity of infection (MOI) of 3 for 1 h, rinsed three times with PBS containing Ca^2+^ and Mg^2+^, then incubated for an additional 24 h in complete medium at 37 °C. The cell supernatant was clarified by centrifugation, aliquoted, and frozen at −80 °C.

Pseudotyped VSV was produced as previously described^3^ to evaluate cohorts i–iv. In brief, HEK293T cells were split into poly-d-lysine-coated 15-cm plates and grown overnight until they reached approximately 70–80% confluency. The cells were washed 3 times with Opti-MEM (Gibco) and transfected with either the Wu-G614, Delta, BA.1, BA.2, BA.4/5, BA.2.75.2, BQ.1.1, XBB.1, or XBB.1.5 S constructs using Lipofectamine 2000 (Life Technologies). After 4–6 h, the medium was supplemented with an equal volume of DMEM supplemented with 20% FBS and 2% penicillin-streptomycin. The cells were incubated for 20–24 h, washed 3 times with DMEM, and infected with VSVΔG-luc. Two hours after VSVΔG-luc infection, the cells were washed an additional five times with DMEM. The cells were grown in DMEM supplemented with anti-VSV-G antibody (I1-mouse hybridoma supernatant diluted 1:25, from CRL-2700, ATCC) for 18–24 h, after which the supernatant was harvested and clarified by low-speed centrifugation at 2,500*g* for 10 min. The supernatant was then filtered (0.45 μm) and some virus stocks were concentrated 10 times using a 30-kDa centrifugal concentrator (Amicon Ultra). The pseudotyped viruses were then aliquoted and frozen at −80 °C.

### VSV pseudovirus neutralization

For cohorts v–viii, DP, KTP and HCW, and monoclonal antibodies, Vero E6 cells were grown in DMEM supplemented with 10% FBS and seeded into white-walled 96-well plates (PerkinElmer, 6005688) at a density of 20,000 cells per well. The next day, monoclonal antibodies were serially diluted in pre-warmed complete medium, mixed with pseudoviruses and incubated for 1 h at 37 °C in round bottom polypropylene plates. Medium from cells was aspirated and 50 µl of pseudovirus–monoclonal antibody complexes were added to cells, which were then incubated for 1 h at 37 °C. An additional 100 µl of pre-warmed complete medium was then added on top of complexes and cells were incubated for an additional 16–24 h. Conditions were tested in duplicate or triplicate wells on each plate and 6–8 wells per plate contained untreated infected cells (defining the 0% of neutralization (MAX RLU) value) and uninfected cells (defining the 100% of neutralization (MIN RLU) value). Virus–monoclonal antibody-containing medium was then aspirated from cells and 50 or 100 µl of a 1:2 dilution of SteadyLite Plus (PerkinElmer) or Bio-Glo (Promega) in PBS with Ca^2+^ and Mg^2+^ was added to cells. Plates were incubated for 15 min at room temperature and then analysed on the Synergy-H1 (BioTek). The average relative light units (RLU) of untreated infected wells (MAX RLUave) were subtracted by the average of MIN RLU (MIN RLUave) and used to normalize percentage of neutralization of individual RLU values of experimental data according to the following formula: (1 − (RLUx – MIN RLUave)/(MAX RLUave – MIN RLUave)) × 100. Data were analysed with Microsoft Excel (v16) and Prism (v.9.1.0). IC_50_ values were calculated from the interpolated value from the log(inhibitor) versus response, using variable slope (four parameters) non-linear regression with an upper constraint of <100. Each neutralization experiment was conducted on at least two independent experiments—that is, biological replicates—in which each biological replicate contains a technical duplicate or triplicate.

For cohorts i–iv, Vero E6-TMPRSS2 were split into white-walled, clear-bottom 96-well plates (Corning) and grown overnight until they reached approximately 70% confluency. Plasma was diluted in DMEM starting at a 1:10 dilution and serially diluted in DMEM at a 1:3 dilution thereafter. Pseudotyped VSV was diluted at a 1:25 to 1:100 ratio in DMEM and an equal volume was added to the diluted plasma. The virus–plasma mixture was incubated for 30 min at room temperature and added to the Vero E6-TMPRSS2 cells. After two hours, an equal volume of DMEM supplemented with 20% FBS and 2% penicillin-streptomycin was added to the cells. After 20–24 h, ONE-Glo EX (Promega) was added to each well and the cells were incubated for 5 min at 37 °C. Luminescence values were measured using a BioTek Synergy Neo2 plate reader. Luminescence readings from the neutralization assays were normalized and analysed using GraphPad Prism 9.1.0. The RLU values recorded from uninfected cells were used to define 100% neutralization and RLU values recorded from cells infected with pseudovirus without plasma were used to define 0% neutralization. ID_50_ were determined from the normalized data points using a [inhibitor] versus normalized response–variable slope model using at least two technical repeats to generate the curve fits. At least two biological replicates with two distinct batches of pseudovirus were conducted for each sample.

### Neutralization of authentic SARS-CoV-2 viruses

Vero E6-TMPRSS2 cells were seeded into black-walled, clear-bottom 96-well plates at 20,000 cells per well and cultured overnight at 37 °C. The next day, 9-point fourfold serial dilutions of monoclonal antibodies were prepared in growth medium (DMEM + 10% FBS). The different SARS-CoV-2 strains were diluted in infection medium (DMEM + 2% BSA) at a final MOI of 0.01 plaque-forming units per cell, added to the monoclonal antibody dilutions and incubated for 30 min at 37 °C. Medium was removed from the cells, monoclonal antibody–virus complexes were added and incubated at 37 °C for 18 h (WA-1 and XBB.1.5) or 24 h (BA.2). Cells were fixed with 4% PFA (Electron Microscopy Sciences, 15714S), permeabilized with Triton X-100 (SIGMA, X100-500ML) and stained with an antibody against the viral nucleocapsid protein (Sino Biologicals, 40143-R001) followed by a staining with the nuclear dye Hoechst 33342 (Fisher Scientific, H1399) and a goat anti-rabbit Alexa Fluor 647 antibody (Invitrogen, A-21245). Plates were imaged on a Cytation5 plate reader. Whole well images were acquired (12 images at 4× magnification per well) and nucleocapsid-positive cells were counted using the manufacturer’s software.

### Pseudotyped VSV entry assays with protease inhibitors

Vero E6-TMPRSS2 or HEK293T-ACE2 were split into white-walled, clear-bottom 96-well plates (Corning) at a density of 18,000 or 36,000 cells, respectively, and grown overnight. The following day, the growth medium was removed and, for assays conducted with Vero E6-TMPRSS2, the cells washed once with DMEM. The cells were incubated for 2 h with DMEM containing 50 µM of Camostat (Sigma), Nafamostat (Sigma), E64d (Sigma), or 0.5% DMSO. All three protease inhibitors were dissolved in DMSO to a concentration of 10 mM and diluted in DMEM. The protease inhibitors were removed and pseudovirus diluted 1:50 or 1:200 in DMEM was added to the cells. After 2 h, an equal volume of DMEM supplemented with 20% FBS and 2% penicillin-streptomycin was added to the cells. After 20–24 h, ONE-Glo EX (Promega) was added to each well and the cells were incubated for 5 min at 37 °C. Luminescence values were measured using a BioTek Synergy Neo2 plate reader. Luminescence readings from the neutralization assays were normalized and analysed using GraphPad Prism 9.1.0. The RLU values recorded from uninfected cells were used to define 0% infectivity and RLU values recorded from cells incubated with 0.5% DMSO only and infected with pseudovirus were used to define 100% infectivity. Twelve technical replicates were performed for each inhibitor and pseudovirus and at least two biological replicates with two distinct batches of pseudovirus were conducted.

### Recombinant protein production for BLI, FACS and cryo-EM

SARS-CoV-2 RBDs and human ACE2 were produced and purified from Expi293F cells as previously described^3^. In brief, cells were grown to a density of 3 × 10^6^ cells per ml and transfected using the ExpiFectamine 293 Transfection Kit (Thermo Fisher Scientific). Three to five days post-transfection, proteins were purified from clarified supernatants using HisTrap HP affinity columns (Cytiva) and washed with ten column volumes of 20 mM imidazole, 25 mM sodium phosphate pH 8.0, and 300 mM NaCl before elution on a gradient to 500 mM imidazole, 25 mM sodium phosphate pH 8.0, and 300 mM NaCl. Proteins were buffer exchanged into 20 mM sodium phosphate pH 8 and 100 mM NaCl and concentrated using centrifugal filters (Amicon Ultra) before being flash frozen.

For cryo-EM, recombinant was expressed in ExpiCHO-S cells at 37 °C and 8% CO_2_ with kifunensine added to 10 µM. Cell culture supernatant was collected eight days post-transfection, supplemented with buffer to a final concentration of 80 mM Tris-HCl pH 8.0, 100 mM NaCl, and then incubated with BioLock (IBA) solution. ACE2 was purified using a 5-ml StrepTrap HP column (Cytiva) followed by isolation of the monomeric ACE2 by size-exclusion chromatography using a Superdex 200 Increase 10/300 GL column (Cytiva) pre-equilibrated in PBS.

Recombinant S309 Fab used for cryo-EM was expressed by ATUM Bio using HEK293-derived suspension cells (lacking the N55Q mutation introduced for improving its manufacturability), purified using CaptureSelect IgG-CH1 resin and buffer exchanged into PBS (ATUM Bio).

### Recombinant protein production for SPR binding assays and antigen-specific MBC repertoire analysis by ELISA

Proteins were expressed in Expi293F cells (Thermo Fisher Scientific) at 37 °C and 8% CO_2_. Transfections were performed using the ExpiFectamine 293 Transfection Kit (Thermo Fisher Scientific). Cell culture supernatants were collected 4–5 days after transfection and supplemented with 10× PBS to a final concentration of 2.5× PBS (342.5 mM NaCl, 6.75 mM KCl and 29.75 mM phosphates). SARS-CoV-2 RBDs were purified by IMAC using Cobalt or Nickel resin followed by buffer exchange into PBS using Amicon centrifugal filters (Milipore Sigma) or by size-exclusion chromatography using a Superdex 200 Increase 10/300 GL column (Cytiva). For SPR binding measurements, recombinant ACE2 (residues 19–615 from Uniprot entry Q9BYF1 with a C-terminal thrombin cleavage site–TwinStrep–10×His–GGG tag and N-terminal signal peptide) was expressed in Expi293F cells at 37 °C and 8% CO_2_. Transfection was performed using the ExpiFectamine 293 Transfection Kit (Thermo Fisher Scientific). Cell culture supernatant was collected 7–8 days after transfection, supplemented to a final concentration of 80 mM Tris-HCl pH 8.0, 100 mM NaCl, and then incubated with BioLock solution (IBA GmbH). ACE2 was purified using a 1-ml StrepTrap HP column (Cytiva) followed by isolation of the monomeric ACE2 by size-exclusion chromatography using a Superdex 200 Increase 10/300 GL column (Cytiva) pre-equilibrated in PBS. Recombinant S309 Fab used for SPR binding studies was produced in either ExpiCHO-S cells and purified using a Capture Select CH1-XL MiniChrom Column (Thermo Fisher), followed by buffer exchange into PBS using a HiPrep 26/10 Desalting Column (Cytiva) or in HEK293 suspension cells, purified using CaptureSelect IgG-CH1 resin and buffer exchanged into PBS (ATUM Bio).

### Biolayer interferometry

BLI was used to assess binding of SARS-CoV-2 RBDs to human ACE2 using an Octet Red96 (Sartorius) and the Octet Data acquisition v11.1. Biotinylated Wu, BA.4/5, BA.2.75.2, BQ.1.1, XBB.1, and XBB.1.5 RBDs were diluted to a concentration of 5 ng µl^−1^ in 10X Octet kinetics buffer (Sartorius) and loaded onto pre-hydrated Streptavidin biosensors to a 1 nm total shift. The loaded tips were dipped into a 1:3 dilution series of monomeric human ACE2 starting at 900 nM or 300 nM for 300 s followed by dissociation in 10× kinetic buffer for 300 s. All steps of the affinity measurements using BLI were carried out at 30 °C with a shaking speed of 1,000 rpm. The resulting data were baseline subtracted and affinity measurements were calculated using a 1:1 global fit binding model with Octet Data Analysis HT software v12.0. Binding curves were plotted using GraphPad Prism 9.1.0.

### In vivo studies

Mouse studies were carried out in accordance with the recommendations in the Guide for the Care and Use of Laboratory Animals of the National Institutes of Health. The protocols were approved by the Institutional Animal Care and Use Committee at the Washington University School of Medicine (assurance number A3381–01). Virus inoculations were performed under anaesthesia that was induced and maintained with ketamine hydrochloride and xylazine, and all efforts were made to minimize animal suffering. Heterozygous K18-hACE2 C57BL/6 J mice (strain: 2B6.Cg-Tg(K18-ACE2)2Prlmn/J) were obtained from The Jackson Laboratory. All animals were housed in groups of 3 to 5 and fed standard chow diets. The photoperiod was 12 h on/12 h off dark/light cycle. The ambient animal room temperature was 21 °C, controlled within ±1 °C and the room humidity was 50%, controlled within ±5%. Eight- to ten-week-old female K18-hACE2 mice were administered indicated doses of S309 or isotype control (anti-WNV hE16^51^) antibody by intraperitoneal injection one day before intranasal inoculation with 10^4^ FFU of BQ.1.1. Weight was recorded daily, and animals were euthanized on day 6 after virus inoculation.

All hamster experiments were performed according to the French legislation and in compliance with the European Community Council Directives (2010/63/UE, French Law 2013–118, 6 February 2013) and according to the regulations of Pasteur Institute Animal Care Committees. The Animal Experimentation Ethics Committee (CETEA 89) of the Institut Pasteur approved this study (200023) before experiments were initiated. Hamsters were housed by groups of 4 animals and manipulated in class III biosafety cabinets in the Pasteur Institute animal facilities accredited by the French Ministry of Agriculture for performing experiments on live rodents. All animals were handled in strict accordance with good animal practice. Male golden Syrian hamsters (*Mesocricetus auratus;* RjHan:AURA) of 5–6 weeks of age (average weight 60–80 grams) were purchased from Janvier Laboratories and handled under specific pathogen-free conditions. The animals were housed and manipulated in isolators in a Biosafety level-3 facility, with ad libitum access to water and food. Before manipulation, animals underwent an acclimation period of one week. Twenty-four hours before infection, the hamsters received an intraperitoneal injection of different concentrations of the monoclonal antibodies S309 (0.6, 1.7, 5 and 15 mg kg^−1^, hamster IgG2a), or the control isotype MPE8 (15 mg kg^−1^, hamster IgG2a). Animal infection was performed as previously described^56^. In brief, the animals were anaesthetized with an intraperitoneal injection of 200 mg kg^−1^ ketamine (Imalgène 1000, Merial) and 10 mg kg^−1^ xylazine (Rompun, Bayer), and 100 µl of physiological solution containing 6 × 10^4^ plaque-forming units of SARS-CoV-2/Omicron_XBB.1.5 (GISAID ID: EPI_ISL_16353849, kindly provided by O. Schwartz and colleagues) was then administered intranasally to each animal (50 µl per nostril). Mock-infected animals received the physiological solution only. Infected and mock-infected hamsters were housed in separated isolators and were followed-up daily, for four days, when the body weight and the clinical score were noted. At day 4 post-inoculation, the animals were euthanized with an excess of anaesthetics (ketamine and xylazine) and exsanguination. Blood samples were collected by cardiac puncture; after coagulation, the tubes were centrifuged at 2,000*g* during 10 min at 4 °C, the serum was collected and frozen at −80 °C until further analyses. The lungs were collected, weighted and frozen at −80 °C until further analyses.

### Measurement of viral RNA levels

Mouse tissues were weighed and homogenized with zirconia beads in a MagNA Lyser instrument (Roche Life Science) in 1 ml of DMEM medium supplemented with 2% heat-inactivated FBS. Tissue homogenates were clarified by centrifugation at approximately 10,000*g* for 5 min and stored at −80 °C. RNA was extracted using the MagMax mirVana Total RNA isolation kit (Thermo Fisher Scientific) on the Kingfisher Flex extraction robot (Thermo Fisher Scientific). RNA was reverse transcribed and amplified using the TaqMan RNA-to-CT 1-Step Kit (Thermo Fisher Scientific). Reverse transcription was carried out at 48 °C for 15 min followed by 2 min at 95 °C. Amplification was accomplished over 50 cycles as follows: 95 °C for 15 s and 60 °C for 1 min. Copies of total (genomic and subgenomic) SARS-CoV-2 *N* gene RNA in samples were determined using a previously published assay^57^. In brief, a TaqMan assay was designed to target a highly conserved region of the *N* gene (forward primer: ATGCTGCAATCGTGCTACAA; reverse primer: GACTGCCGCCTCTGCTC; probe: /56-FAM/TCAAGGAAC/ZEN/AACATTGCCAA/3IABkFQ/). This region was included in an RNA standard to allow for copy number determination down to 10 copies per reaction. The reaction mixture contained final concentrations of primers and probe of 500 and 100 nM, respectively.

For hamster studies, frozen lung fragments were weighted and homogenized with 1 ml of ice-cold DMEM (31966021, Gibco) supplemented with 1% penicillin/streptomycin (15140148, Thermo Fisher) in Lysing Matrix M 2 ml tubes (116923050-CF, MP Biomedicals) using the FastPrep-24 system (MP Biomedicals), and the following scheme: homogenization at 4.0 m s^−1^ for 20 s, incubation at 4 °C for 2 min, and new homogenization at 4.0 m s^−1^ for 20 s. The tubes were centrifuged at 10,000*g* for 1 min at 4 °C. Afterwards, 125 µl of the tissue homogenate supernatant were mixed with 375 μl of Trizol LS (10296028, Invitrogen) and the total RNA was extracted using the Direct-zol RNA MiniPrep Kit (R2052, Zymo Research). The presence of SARS-CoV-2 RNA in these samples was evaluated by one-step quantitative PCR with reverse transcription in a final volume of 12.5 μl per reaction in 384-wells PCR plates using a thermocycler (QuantStudio 6 Flex, Applied Biosystems). In brief, 2.5 μl of RNA were added to 10 μl of a master mix containing 6.25 μl of 2× reaction mix, 0.2 μl of MgSO_4_ (50 mM), 0.5 μl of Superscript III RT/Platinum Taq Mix (2 UI µl^−1^) and 3.05 μl of nuclease-free water containing the nCoV_IP2 primers (nCoV_IP2-12669Fw: 5′-ATGAGCTTAGTCCTGTTG-3′; nCoV_IP2-12759Rv: 5′-CTCCCTTTGTTGTGTTGT-3′) at a final concentration of 400 nM, and the nCoV_IP2 probe (5′-FAM-AGATGTCTTGTGCTGCCGGTA-3′-TAMRA) at a final concentration of 200 nM. The amplification conditions were as follows: 55 °C for 20 min, 95 °C for 3 min, 50 cycles of 95 °C for 15 s and 58 °C for 30 s, and a last step of 40 °C for 30 s. Viral load quantification (expressed as RNA copy number per mg of tissue) was assessed by linear regression using a standard curve of six known quantities of RNA transcripts containing the *RdRp* sequence (ranging from 10^7^ to 10^2^ copies).

### Viral plaque assay

Vero E6-TMPRSS2-ACE2 cells were seeded at a density of 1 × 10^5^ cells per well in 24-well tissue culture plates. The following day, medium was removed and replaced with 200 μl of material to be titrated diluted serially in DMEM supplemented with 2% FBS. One hour later, 1 ml of methylcellulose overlay was added. Plates were incubated for 72 h, and then fixed with 4% paraformaldehyde (final concentration) in PBS for 20 min. Plates were stained with 0.05% (w/v) crystal violet in 20% methanol and washed twice with distilled, deionized water.

### End-point virus titration in hamsters

Lung tissues were homogenized as described above for measurement of viral RNA. To quantify infectious SARS-CoV-2 particles, lung homogenates titrations were performed on confluent Vero E6 cells in 96- well plates. Viral titres were expressed as 50% tissue culture infectious dose (TCID_50_) per mg tissue^58^.

### Transient expression of recombinant SARS-CoV-2 S and flow cytometry

ExpiCHO-S cells were seeded at 6 × 10^6^ cells per ml in a volume of 5 ml in a 50-ml bioreactor. The following day, cells were transfected with SARS-CoV-2 S glycoprotein-encoding pcDNA3.1(+) plasmids (BetaCoV/Wuhan-Hu-1/2019, accession number MN908947, Wu-D614; Omicron BA.2, BQ.1.1, XBB.1, XBB.1.5, BN.1 or BA.2-E340A generated by overlap PCR mutagenesis of the Wu-D614 plasmid) harbouring the Δ19 C-terminal truncation. S-encoding plasmids were diluted in cold OptiPRO SFM (Life Technologies, 12309-050), mixed with ExpiFectamine CHO Reagent (Life Technologies, A29130) and added to cells. Transfected cells were then incubated at 37 °C with 8% CO_2_ with an orbital shaking speed of 250 rpm (orbital diameter of 25 mm) for 24 to 48 h. Transiently transfected ExpiCHO-S cells were harvested and washed twice in wash buffer (PBS 2% FBS, 2mM EDTA). Cells were counted and distributed into round bottom 96-well plates (Corning, 3799) and incubated with serial dilutions of mAb starting at 10 μg ml^−1^. Alexa Fluor 647-labelled Goat anti-human IgG secondary antibody (Jackson ImmunoResearch, 109-606-098) was prepared at 2 μg ml^−1^ and added onto cells after two washing steps. Cells were then washed twice and resuspended in wash buffer for data acquisition at Ze5 cytometer (Bio-Rad).

### Measurement of effector functions triggered by monoclonal antibodies

ADCC assays were performed using ExpiCHO-S cells transiently transfected with SARS-CoV-2 S glycoproteins (Wu-D614, BA.2, BQ.1.1, XBB.1, XBB.1.5, BN.1 or BA.2-E340A) as target cells. Natural killer cells were isolated from fresh blood of healthy donors using the MACSxpress WB NK cell isolation kit, human (Miltenyi Biotec, 130-127-695). Target cells were incubated with titrated concentrations of monoclonal antibody for 10 min and then with primary human natural killer cells at an effector to target ratio ranging from 6:1 to 9:1. ADCC was measured using the LDH release assay (Cytotoxicity Detection Kit (LDH) (Roche, 11644793001)) after 4 h incubation at 37 °C.

ADCP assays were performed using ExpiCHO-S cells transiently transfected with SARS-CoV-2 S glycoproteins and labelled with PKH67 (Sigma-Aldrich) as target cells. PMBCs from healthy donors were labelled with CellTrace Violet (Invitrogen) and used as source of phagocytic effector cells. Target cells (10,000 per well) were incubated with titrated concentrations of monoclonal antibody for 10 min and then mixed with PBMCs (200,000 per well). The next day, cells were stained with APC-labelled anti-CD14 antibody (BD Pharmingen), BV605-labelled anti-CD16 antibody (Biolegend), BV711-labelled anti-CD19 antibody (Biolegend), PerCP/Cy5.5-labelled anti-CD3 antibody (Biolegend), APC/Cy7-labelled anti-CD56 antibody (Biolegend) for the identification of CD14^+^ monocytes. After 20 min, cells were washed and fixed with 4% paraformaldehyde before acquisition on a ZE5 Cell Analyzer (Bio-Rad). Data were analysed using FlowJo software (v10.8.1). The percentage ADCP was calculated as the percentage of monocytes (CD3^−^CD19^–^CD14^+^ cells) positive for PKH67.

### Measurement of effector functions triggered by plasma antibodies

Antibody-dependent activation of human FcγRIIIa by plasma antibodies was quantified using a bioluminescent reporter assay. ExpiCHO-S cells transiently expressing full-length SARS-CoV-2 S from Wu-D614, BA.5, BQ.1.1 or XBB.1 (target cells) were incubated with serial dilutions of plasma from immune donors. After a 20-min incubation, Jurkat reporter cells stably expressing FcγRIIIa V158 and a NFAT-driven luciferase reporter gene (effector cells) were added at an effector to target ratio of 6:1. Signalling was quantified by the luciferase signal produced via activation of the NFAT pathway. Luminescence was measured after 22 h of incubation at 37 °C with 5% CO_2_ with a luminometer using the Bio-Glo-TM Luciferase Assay Reagent according to the manufacturer’s instructions (Promega).

Natural killer cell-mediated ADCC induced by plasma antibodies was measured as described for ADCC except that ExpiCHO-S cells transiently expressing full-length SARS-CoV-2 S from Wu-D614, BA.1, BA.5, BA.2.75.2, BQ.1.1 or XBB.1 (target cells) were incubated with plasma from immune donors at a single dilution (1:200).

### Antigen-specific MBC repertoire analysis of secreted IgGs

Replicate cultures of total unfractionated PBMCs obtained from SARS-CoV-2 infected and/or vaccinated individuals were seeded in 96 U-bottom plates (Corning) in RPMI1640 supplemented with 10% fetal calf serum (Hyclone), sodium pyruvate, MEM non-essential amino acids, stable glutamine, 2-mercaptoethanol, penicillin-streptomycin, kanamycin and transferrin. MBC stimulation and differentiation was induced by adding 2.5 μg ml^−1^ R848 (3 M) and 1,000 U ml^−1^ human recombinant IL-2 at 37 °C and 5% CO_2_, as previously described^59^. After 10 days, the cell culture supernatants were collected for ELISA analysis.

### Enzyme-linked immunosorbent assay

Ninety-six half-area well plates (Corning, 3690) were coated overnight at 4 °C with 25 μl of sarbecovirus RBDs prepared at 5 μg ml^−1^ in PBS pH 7.2. Plates were then blocked with PBS 1% BSA (Sigma-Aldrich, A3059) and subsequently incubated with serial dilutions of monoclonal antibodies for 1 h at room temperature. After 4 washing steps with PBS 0.05% Tween-20 (PBS-T) (Sigma-Aldrich, 93773), goat anti-human IgG-AP secondary antibody (Southern Biotech, 2040-04, diluted 1/500) was added and incubated for 1 h at room temperature. Plates were then washed four times with PBS-T and 4-nitrophenyl phosphate (pNPP, Sigma-Aldrich, 71768) substrate was added. After 30 min incubation, absorbance at 405 nm was measured using a plate reader (BioTek) and data were plotted using Prism GraphPad 9.1.0. To test plasma and MBC-derived antibodies, Spectraplate-384 with high protein binding treatment (custom made from PerkinElmer) were coated overnight at 4 °C with 3 µg ml^−1^ of different SARS-CoV-2 RBDs (produced in house) and S trimers (Acrobiosystems AG, SPN-C52H3, SPN-C522a, SPN-C522e, SPN-C522r, SPN-C522s, SPN-C522t and SPN-C524i) in PBS pH 7.2 or PBS alone as control. Plates were subsequently blocked with Blocker Casein (1%) in PBS (Thermo Fisher Scientific, 37528) supplemented with 0.05% Tween-20 (Sigma-Aldrich, 93773-1KG). The coated plates were incubated with diluted B cell supernatant for 1 h at room temperature. Plates were washed with PBS containing 0.05% Tween-20 (PBS-T), and binding was revealed using secondary goat anti-human IgG-AP (Southern Biotech, 2040-04). After washing, pNPP substrate (Sigma-Aldrich, 71768-25G) was added and plates were read at 405 nm after 1 h or 30 min.

### Blockade of RBD binding to human ACE2

MBC culture supernatants were diluted in PBS and mixed with SARS-CoV-2 Wu RBD mouse Fc-tagged antigen (Sino Biological, 40592-V05H) or with biotinylated BQ.1.1 or XBB.1 RBDs (Acrobiosystems) at a final concentration of 20 ng ml^−1^ and incubated for 30 min at 37 °C. The mix was added for 30 min to ELISA 384-well plates (NUNC, P6366-1CS) pre-coated overnight at 4 °C with 4 µg ml^−1^ human ACE2 (produced in house) in PBS. Plates were washed with PBS containing 0.05% Tween-20 (PBS-T), and RBD binding was revealed using secondary goat anti-mouse IgG-AP (Southern Biotech, 1032-04) or Streptavidin-AP (Jackson ImmunoResearch). After washing, pNPP substrate (Sigma-Aldrich, 71768-25G) was added and plates were read at 405 nm after 1 h.

### Blockade of binding to S

Human anti-S monoclonal antibodies (S2V29 for RBD site Ia, SA55 for RBD site IIa, S309 for RBD site IV^23^, S3H3 for domain C/SD1^60^ and S2P6 for the stem helix^61^) were biotinylated using the EZ-Link NHS-PEO solid phase biotinylation kit (Pierce). Labelled monoclonal antibodies were tested for binding to Wu-G614, BQ.1.1 and XBB.1 S by ELISA and the optimal concentration of each monoclonal antibody to achieve 80% maximal binding was determined. Plasma samples were serially diluted and added to ELISA 96-well plates (Corning) pre-coated overnight at 4 °C with 1 µg ml^−1^ of S (Acrobiosystems) in PBS. After 30 min, biotinylated anti-S monoclonal antibodies were added at the concentration achieving 80% maximal binding and the mixture was incubated at room temperature for 30 min. Plates were washed and antibody binding was revealed using alkaline phosphatase-comjugated streptavidin (Jackson ImmunoResearch). After washing, pNPP substrate (Sigma-Aldrich) was added and plates were read at 405 nm. The percentage of inhibition was calculated as follow: (1 − (absorbance of sample − absorbance of negative control)/(absorbance of positive control − absorbance of negative control)) × 100.

### PNGase F reaction to remove N-linked glycans on BN.1 RBD

Twenty micrograms of purified BN.1 RBD was combined with 2 µl PNGase F (500 units per µl, New England BioLabs, P0704S) and 5 µl of 10× GlycoBuffer 3and H_2_O (if necessary) to bring the total reaction volume to 50 μl. The reaction was incubated at room temperature overnight and used for SPR and mass intact mass spectrometry.

### Intact mass spectrometry analysis and liquid chromatography–mass spectrometry analysis

Four micrograms of PNGase F-treated BN.1 RBD was used for each injection on the liquid chromatography–mass spectrometry (LC–MS) system to acquire intact mass spectrometry signal after separation of protease and protein by liquid chromatography (Agilent PLRP-S reversed phase column). Thermo MS (Q Exactive Plus Orbitrap) was used to acquire intact protein mass under denaturing conditions. BioPharma Finder 3.2 software was used to deconvolute the raw *m*/*z* data to protein average mass.

Peptide mapping with LC–MS was used to profile the site-specific glycosylation sites on BN.1 RBD. Glycopeptides containing only one specific glycan were achieved by selectively digesting with chymotrypsin protease. Twenty micrograms of each digest product (peptide with a single glycan) was analysed by LC–MS (Agilent AdvanceBio peptide mapping column and Thermo Q Exactive Plus Orbitrap MS). Peptide mapping data were analysed on Biopharma Finder 3.2 data analysis software.

### SPR assays to measure binding of ACE2 and S309 Fab to RBDs

Measurements were performed using a Biacore T200 instrument or a Biacore 8k instrument using the Biacore Evaluation software (v.3.2.1). CM5 chips with covalently immobilized anti-Avi polyclonal antibody diluted to a final concentration of 25 µg ml^−1^ (GenScript, A00674-40) were used for surface capture of His–Avi tag containing RBDs. Running buffer was HBS-EP+ pH 7.4 (Cytiva) and measurements were performed at 25 °C. Experiments were performed with a fourfold dilution series of monomeric S309 Fab or ACE2 at 300, 75, 18.8 and 4.7 nM and were run as single-cycle kinetics. Data were double reference-subtracted and fit to a binding model using Biacore Insight software (v4.0.8.20368). The 1:1 binding model was used to determine the kinetic parameters. 2–14 replicates were performed for each ligand (RBDs) and analyte (ACE2 or S309 Fab) pair. For BN.1 RBD–S309 Fab binding, due to a low binding signal because of a slow association rate constant, a constant Rmax calculated from a control analyte was applied to calculate the kinetic parameters. *K*_d_ values are reported as the average of all replicates with the corresponding standard deviation (Supplementary Table 2 for ACE2 binding data and Supplementary Table 4 for S309 Fab binding data)

### Cell–cell fusion assay

Cell–cell fusion assays using a split-GFP system was conducted as previously described^3^. In brief, Vero E6-TMPRSS2-GFP_11_ cells were split into 96-well, glass bottom, black-walled plates (CellVis) at a density of 36,000 cells per well. BHK-21-GFP_1–10_ cells were split into 6-well plates at a density of 1 × 10^6^ cells per well. The following day, the growth medium was removed and replaced with DMEM containing 10% FBS and 1% penicillin-streptomycin and the cells were transfected with 4 µg of S protein using Lipofectamine 2000. Twenty-four hours after transfection, BHK-21-GFP_1–10_ cells expressing the S protein were washed three times using FluoroBrite DMEM (Thermo Fisher) and detached using an enzyme-free cell dissociation buffer (Gibco). The Vero E6-TMPRSS2-GFP_11_ were washed 3 times with FluoroBrite DMEM and 9,000 BHK-21-GFP_1–10_ cells were plated on top of the Vero E6-TMPRSS2-GFP_11_ cells. The cells were incubated at 37 °C and 5% CO_2_ in a Cytation 7 plate Imager (BioTek) and both bright-field and GFP images were collected every 30 min for 18 h. Fusogenicity was assessed by measuring the area showing GFP fluorescence for each image using Gen5 Image Prime v3.11 software.

To measure surface expression of the variant SARS-CoV-2 S protein, 1 × 10^6^ transiently transfected BHK-21-GFP_1–10_ cells were collected by centrifugation at 1,000*g* for 5 min. The cells were washed once with PBS and fixed with 2% paraformaldehyde. The cells were washed twice with flow staining buffer (1% BSA, 1 mM EDTA, 0.1% NaN_3_ in PBS) and labelled with 25 µg ml^−1^ S2L20, an NTD-directed antibody that recognizes all currently and previously circulating SARS-CoV-2 variants, for 45 min. The cells were washed three times with flow staining buffer and labelled with a PE-conjugated anti-human IgG Fc antibody (Thermo Fisher) for 30 min. The cells were washed an additional three times and resuspended in flow staining buffer. The labelled cells were analysed using a BD FACSymphony A3. Cells were gated on singleton events and a total of 10,000 singleton events were collected for each sample. The fraction of S-positive cells was determined in FlowJo 10.8.1 by gating singleton events for the mock transfected cells on PE intensity.

### Flow cytometry analysis of SARS-CoV-2 RBD-reactive MBCs

RBD–streptavidin tetramers conjugated to fluorophores were generated by incubating biotinylated Wu, BA.1, BA.2, BA.4/5 or BQ.1.1 with streptavidin at a 4:1 molar ratio for 30 min at 4 °C. Excess free biotin was then added to the reaction to bind any unconjugated sites in the streptavidin tetramers. The RBD-streptavidin tetramers were washed once with PBS and concentrated with a 30-kDa centrifugal concentrator (Amicon). An additional streptavidin tetramer conjugated to biotin only was generated and included in the staining.

Approximately 5 to 15 million PMBCs were collected 5–72 days post-vaccination for individuals who received either the Wu monovalent mRNA booster or Wu/BA.5 bivalent mRNA booster. The cells were collected by centrifugation at 1,000*g* for 5 mins at 4 °C and washed twice with PBS. The cells were then stained with Zombie Aqua dye (Biolegend; diluted 1:100 in PBS) for 30 min at room temperature after which the cells were washed twice with FACS staining buffer (0.1% BSA, 0.1% NaN_3_ in PBS). The cells were then stained with antibodies for CD20-PECy7 (BD), CD3-Alexa eFluor780 (Thermo Fisher), CD8-Alexa eFluor780 (Thermo Fisher), CD14-Alexa eFluor780 (Thermo Fisher), CD16-Alexa eFluor780 (Thermo Fisher), IgM-Alexa Fluor 647 (BioLegend), IgD-Alexa Fluor 647 (BioLegend), and CD38-Brilliant Violet 785 (BioLegend), all diluted 1:200 in Brilliant Stain Buffer (BD), along with the RBD-streptavidin tetramers for 30 min at 4 °C. The cells were washed three times, resuspended in FACS staining buffer, and passed through a 35-µm filter. The cells were examined using a BD FACSAria III and FACSDiva for acquisition and FlowJo 10.8.1 for analysis. Single live CD20^+^CD3^−^CD8^−^CD14^−^CD16^−^IgM^lo^IgD^lo^CD38^lo^RBD^+^ cells were sorted based on reactivity to the Omicron and Wu RBDs into RNAlater and stored at −80 °C.

### Cryo-EM sample preparation, data collection and data processing

Cryo-EM grids of BQ.1.1 RBD–ACE2–S309, XBB.1 RBD–ACE2–S309 or BN.1 RBD–ACE2–S309 complex were prepared fresh after purification by size-exclusion chromatography. For BQ.1.1 RBD–ACE2–S309 complex, 3 μl of 0.25 mg ml^−1^ BQ.1.1 RBD–ACE2–S309 were loaded onto freshly glow-discharged R 2/2 UltrAuFoil grids^62^, prior to plunge freezing using a vitrobot Mark IV (Thermo Fisher Scientific) with a blot force of 0 and 6 s blot time at 100% humidity and 22 °C. Data were acquired using an FEI Titan Krios transmission electron microscope operated at 300 kV and equipped with a Gatan K3 direct detector and Gatan Quantum GIF energy filter, operated in zero-loss mode with a slit width of 20 eV. For BQ.1.1 RBD–ACE2-S309 data set, automated data collection was carried out using Leginon v3.4^63^ at a nominal magnification of 105,000× with a pixel size of 0.843 Å and stage tilt angle of 0° and 30°. 6,487 micrographs were collected with a defocus range comprised between −0.5 and −2.5 μm. For XBB.1 RBD–ACE2–S309 complex, samples were prepared using a Vitrobot Mark IV (Thermo Fisher Scientific) with R 2/2 UltrAuFoil grids and a Chameleon (SPT Labtech) with self-wicking nanowire Cu R1.2/0.8 holey carbon grids. For XBB.1 RBD–ACE2-S309 data set, 6,355 micrographs from UltrAuFoil grids were collected with a defocus range comprised between −0.2 and −3 μm and stage tilt angle of 0° and 30° and 2,889 micrographs from chameleon grids were collected with a defocus range comprised between −0.2 and −3 μm without tilting the stage. For BN.1 RBD–ACE2–S309 complex, samples were prepared using a Vitrobot Mark IV (Thermo Fisher Scientific) with R 2/2 UltrAuFoil grids, manual blotting/plunging with C-flat holey thick carbon grids and Chameleon (SPT Labtech) with self-wicking nanowire Cu R1.2/0.8 holey carbon grids. For BN.1 RBD–ACE2–S309 data set, 3,822 micrographs from UltrAuFoil grids, 2,000, micrographs from chameleon grids and 1,915 micrographs from C-flat holey thick carbon grids were collected with a defocus range comprised between −0.2 and −3.5 μm and stage tilt angle of 0° and 30°. The dose rate was adjusted to 15 counts per pixel per s, and each movie was acquired in super-resolution mode fractionated in 75 frames of 40 ms. Movie frame alignment, estimation of the microscope contrast transfer function parameters, particle picking, and extraction were carried out using Warp^64^ (v1.0.9).

Two rounds of reference-free 2D classification were performed using cryoSPARC^65^ (v4.2.2) to select well-defined particle images. These selected particles were subjected to two rounds of 3D classification with 50 iterations each (angular sampling 7.5° for 25 iterations and 1.8° with local search for 25 iterations) using Relion^66,67^ (v3.1) with an initial model generated with ab-initio reconstruction in cryoSPARC. 3D refinements were carried out using non-uniform refinement^68^ along with per-particle defocus refinement in CryoSPARC. Selected particle images were subjected to the Bayesian polishing procedure^69^ implemented in Relion before performing another round of non-uniform refinement in cryoSPARC followed by per-particle defocus refinement and again non-uniform refinement. To further improve the density of the BQ.1.1 RBD and XBB.1 RBD, the particles were subjected to focus 3D classification without refining angles and shifts using a soft mask encompassing the ACE2, RBD and S309 variable domains using a tau value of 60 in Relion. To further improve the density of the BN.1 RBD, the particles were subjected to cryoSPARC heterogeneous refinement. Particles belonging to classes with the best resolved local density were selected and subjected to non-uniform refinement using cryoSPARC. Local resolution estimation, filtering, and sharpening were carried out using CryoSPARC. Reported resolutions are based on the gold-standard Fourier shell correlation (FSC) with 0.143 criterion and Fourier shell correlation curves were corrected for the effects of soft masking by high-resolution noise substitution^70,71^.

### Model building and refinement

UCSF Chimera^72^ (v1.17.1) and Coot^73^ (v0.9.6) were used to fit atomic models into the cryo-EM maps. RBD, ACE2 and S309 Fab models were refined and relaxed using Rosetta using sharpened and unsharpened maps^74,75^.

### Statistical analysis

All statistical tests were performed as described in the indicated figure legends using Prism v9.1.0. The number of independent experiments performed are indicated in the relevant figure legends. Comparisons of means between multiple groups of unpaired data were made with Kruskal–Wallis rank test and corrected with Dunn’s test. Statistical significance is set as *P* < 0.05, and *P* values are indicated with: NS, not significant; *P < 0.05; **P < 0.01; ***P < 0.001, ****P < 0.0001. ED_50_, 80% of the maximum binding response (BD_80_), ID_50_ and IC_50_ titres were calculated from the interpolated value from the log(agonist) and the log(inhibitor), versus response using variable slope (four parameters) non-linear regression. Data were plotted and analysed with GraphPad Prism software (version 9.1.0).

### Reporting summary

Further information on research design is available in the Nature Portfolio Reporting Summary linked to this article.

## Online content

Any methods, additional references, Nature Portfolio reporting summaries, source data, extended data, supplementary information, acknowledgements, peer review information; details of author contributions and competing interests; and statements of data and code availability are available at 10.1038/s41586-023-06487-6.

### Supplementary information

Supplementary Figures This file contains Supplementary Figures 1-6.

Reporting Summary

Supplementary Tables **This file contains Supplementary Tables 1-8**.

### Source data

Source Data Fig. 3

## Figures and Tables

**Extended Data Table 1:**
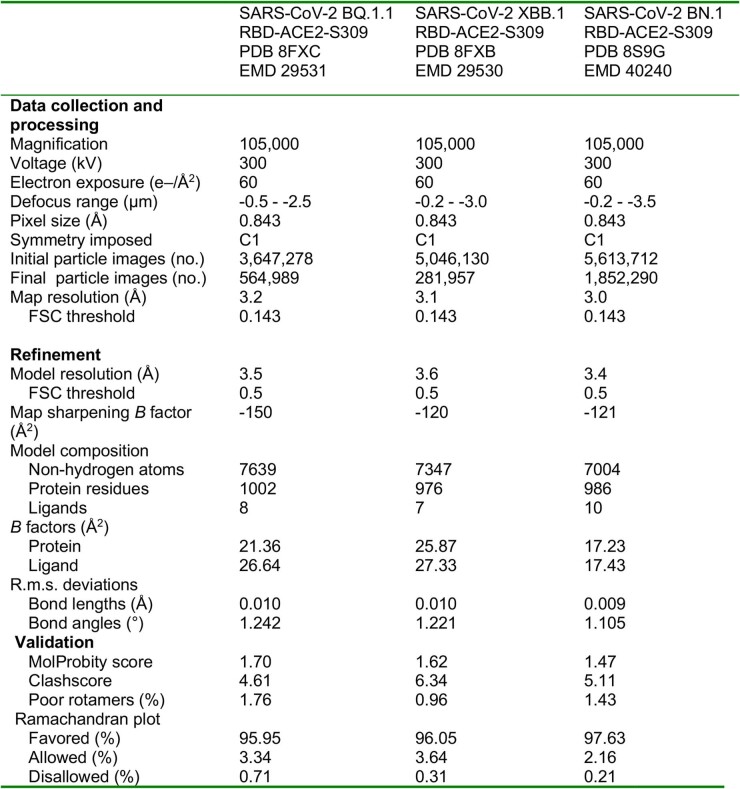
Cryo-EM data collection, refinement and validation statistics

## Data Availability

The cryo-EM maps and atomic coordinates were deposited to the Electron Microscopy Data Bank (EMDB) and the PDB with accession numbers EMD-29531 and 8FXC (BQ.1.1 RBD–ACE2–S309), EMD-29530 and 8FXB (XBB.1 RBD–ACE2–S309), and EMD-40240 and PDB 8S9G (BN.1 RBD–ACE2–S309), respectively. All datasets generated and information presented in the study are available from the corresponding authors on reasonable request. Materials generated in this study can be available on request and may require a material transfer agreement. Source data are provided with this paper.
