# Supplementary material for: Neutralization, effector function and immune imprinting of Omicron variants
Source: Nature. 2023 Aug 30;621(7979):592–601. doi: 10.1038/s41586-023-06487-6 (PMC10511321; doi:10.1038/s41586-023-06487-6)
Supplement: Supplementary file 1 — This file contains Supplementary Figures 1-6. [file 41586_2023_6487_MOESM1_ESM.docx]

**
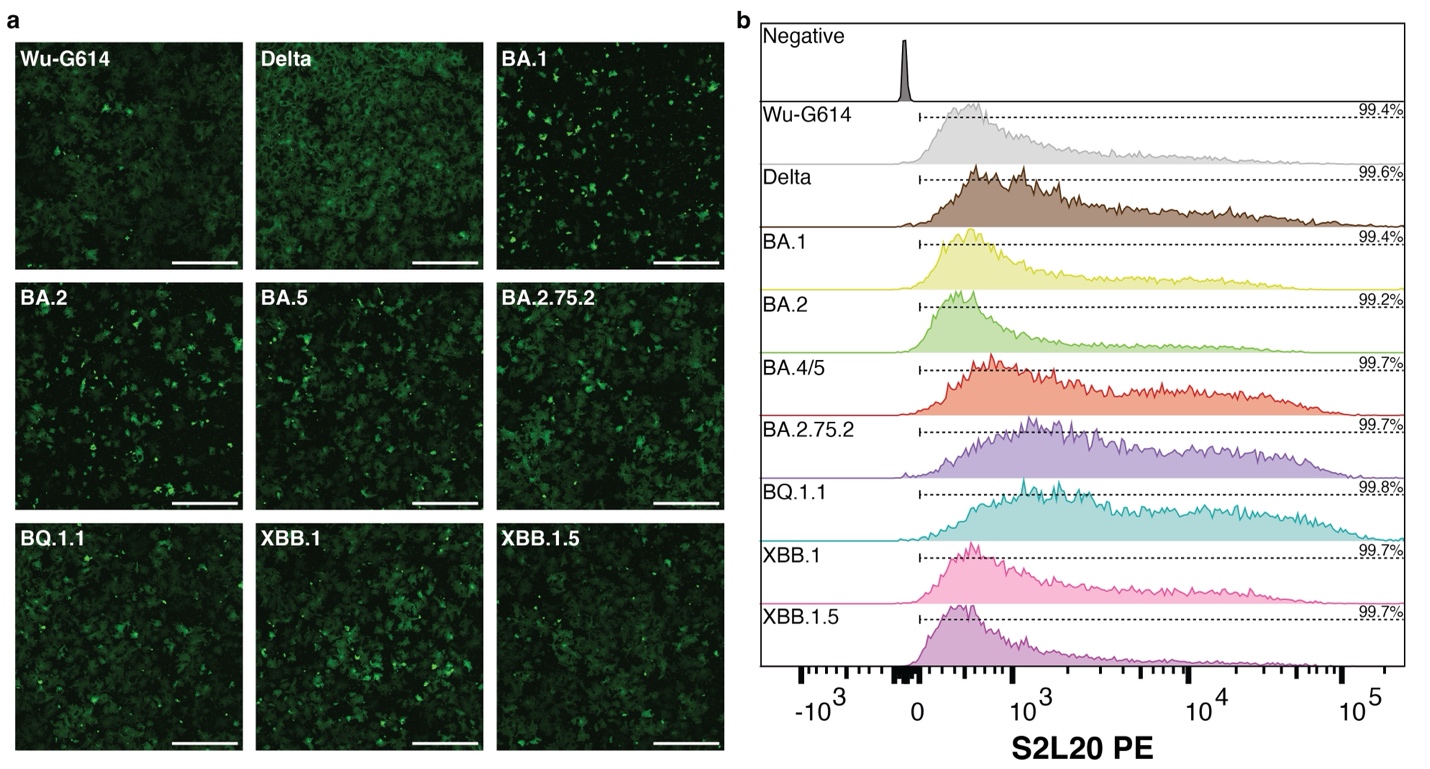
Supplementary Figure 1.** **Membrane fusion assay. a,** Representative cell-cell fusion images showing reconstitution of GFP fluorescence after 18 h. Scale bar: 1 mm. Six images were collected at each 30 minute interval for each variant S analyzed and two independent biological replicates were conducted. **b,** Quantification of SARS-CoV-2 S surface expression by flow cytometry using the NTD-directed Ab S2L20^8,54^ for BHK-21 GFP_1-10_ cells transfected with Wu-G614, Delta, BA.1, BA.2, BA.4/5, BA.2.75.2, BQ.1.1, XBB.1, or XBB.1.5 S proteins. The y-axis is presented as a modal scale scaled to maximum singleton events for that plot. The percentage of S-positive cells is indicated in the top right corner based on the PE intensity relative to mock transfected (negative) cells and represented by the dashed line above each plot.


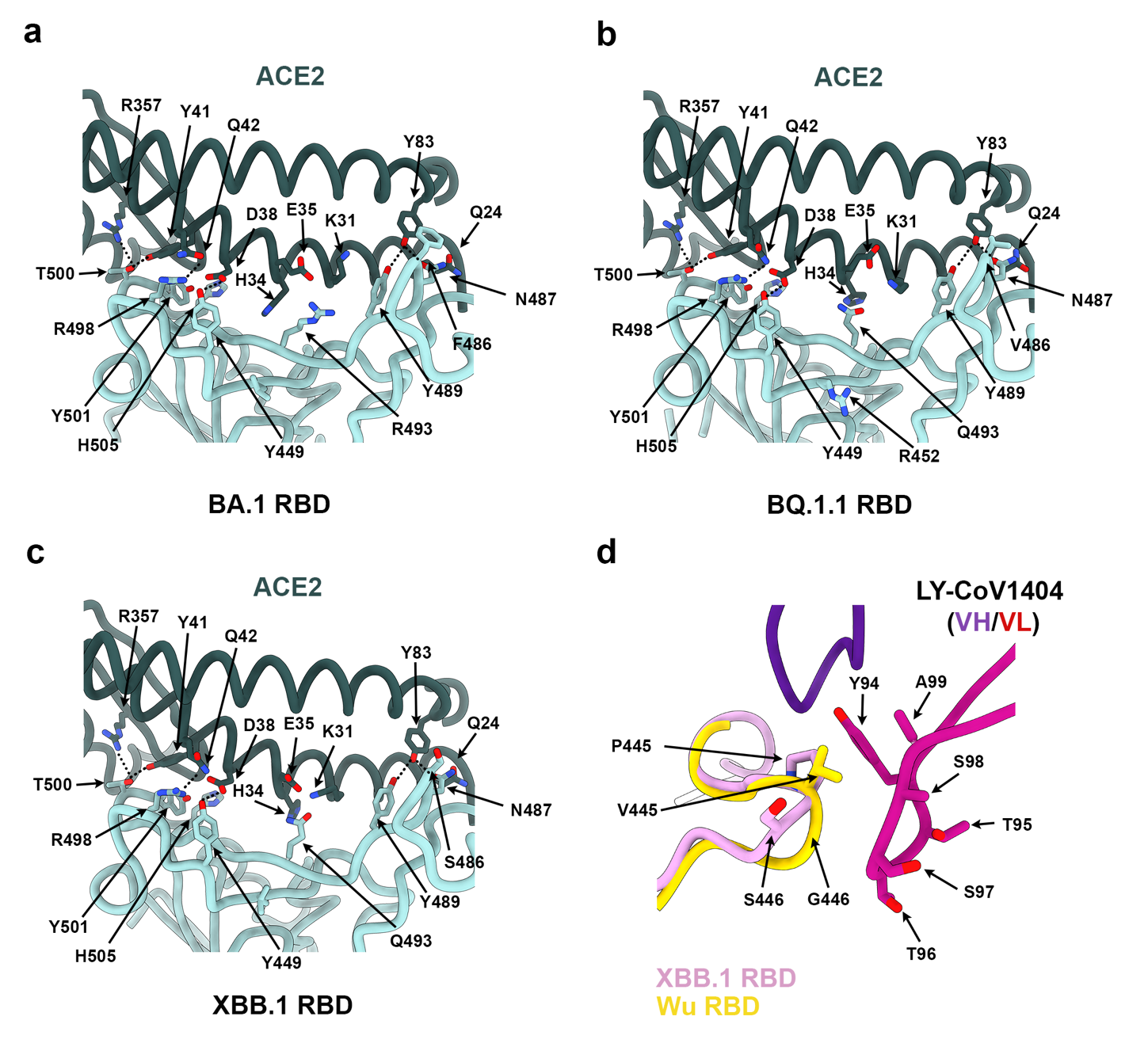


**Supplementary Figure 2. Structural comparisons of the Wu, BA.1, BQ.1.1 and XBB.1 RBD interactions with human ACE2 or therapeutic antibodies. a-c,** Zoomed-in views of the BA.1 RBD (PDB 7TN0, cyan), BQ.1.1 RBD (cyan) or XBB.1 RBD (cyan) interactions with the human ACE2 ectodomain (green). Select side chains are shown and electrostatic interactions are highlighted with dotted lines**. d,** Superimposition of the LYCoV1404-bound Wu RBD (gold, PDB 7MMO) crystal structure to the ACE2- and S309-bound XBB.1 RBD (pink) cryoEM structure (S309 and ACE2 are not shown for clarity).


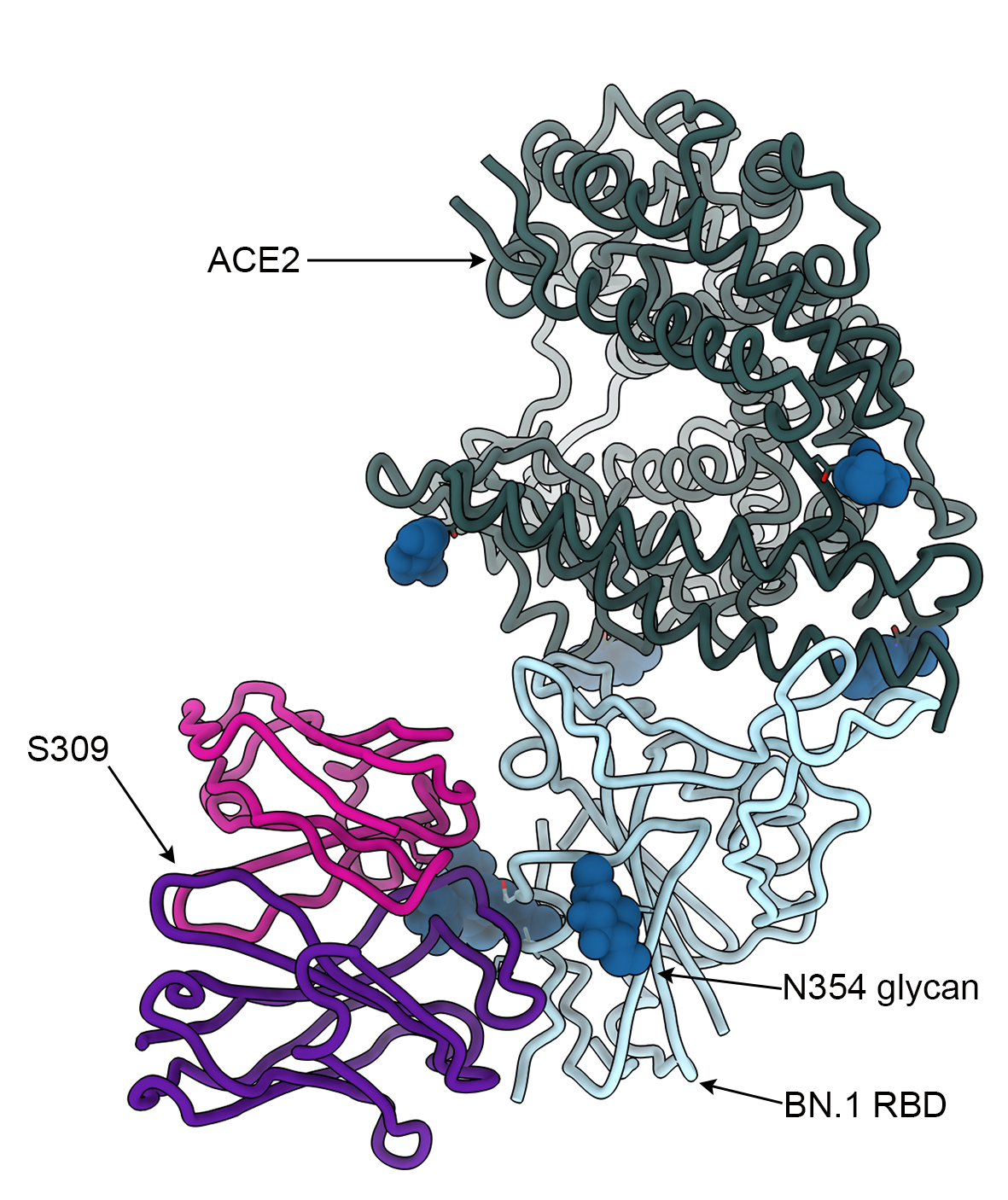


**Supplementary Figure 3.** BN.1 cryoEM structure showing the position of the N354 glycan relative to S309.

**
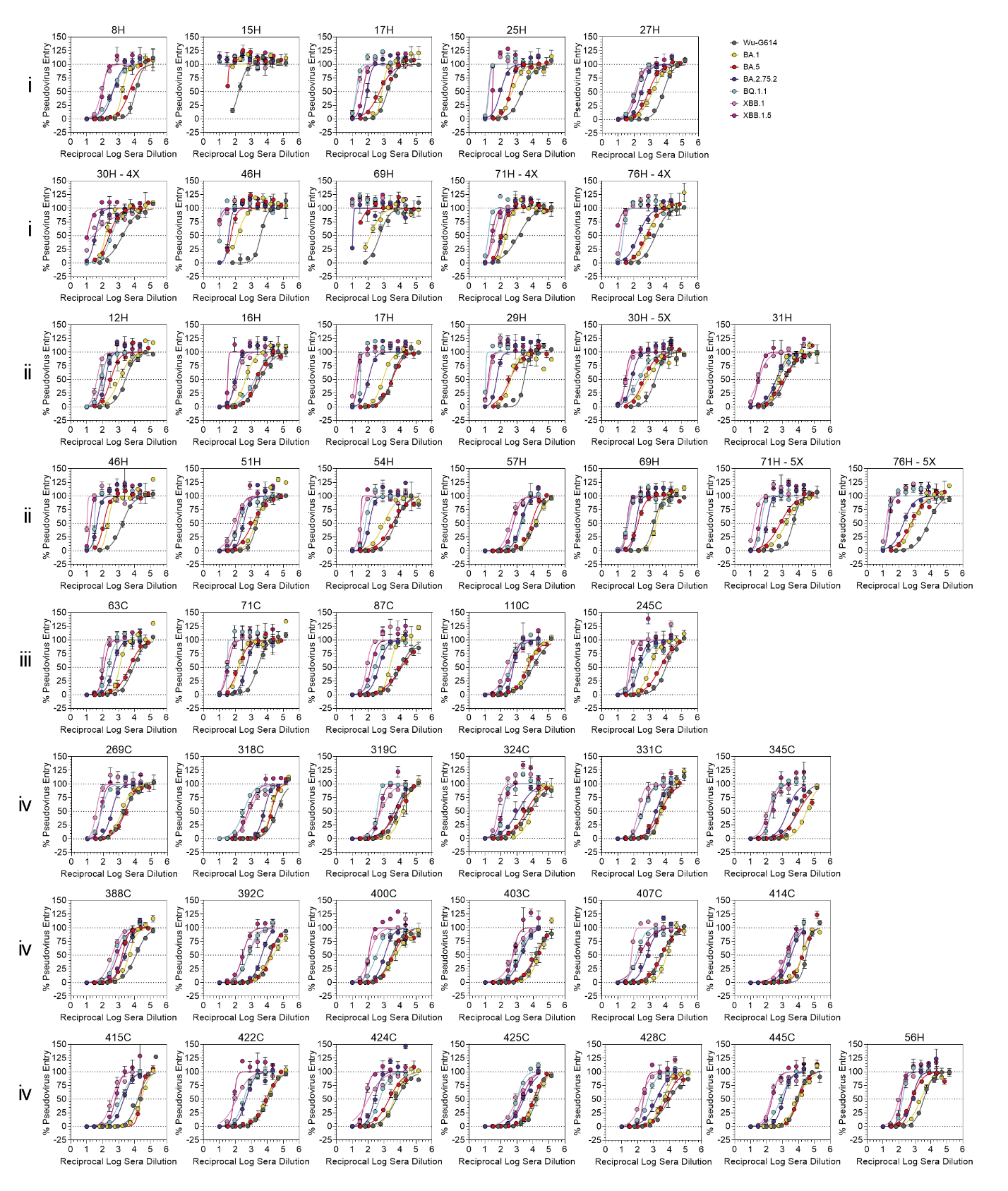
**

**Supplementary Fig. 4.** Dose-response plasma neutralization curves for cohorts i-iv. Means of duplicates ± standard deviation of one representative experiment of at least two biological replicates are presented.

**
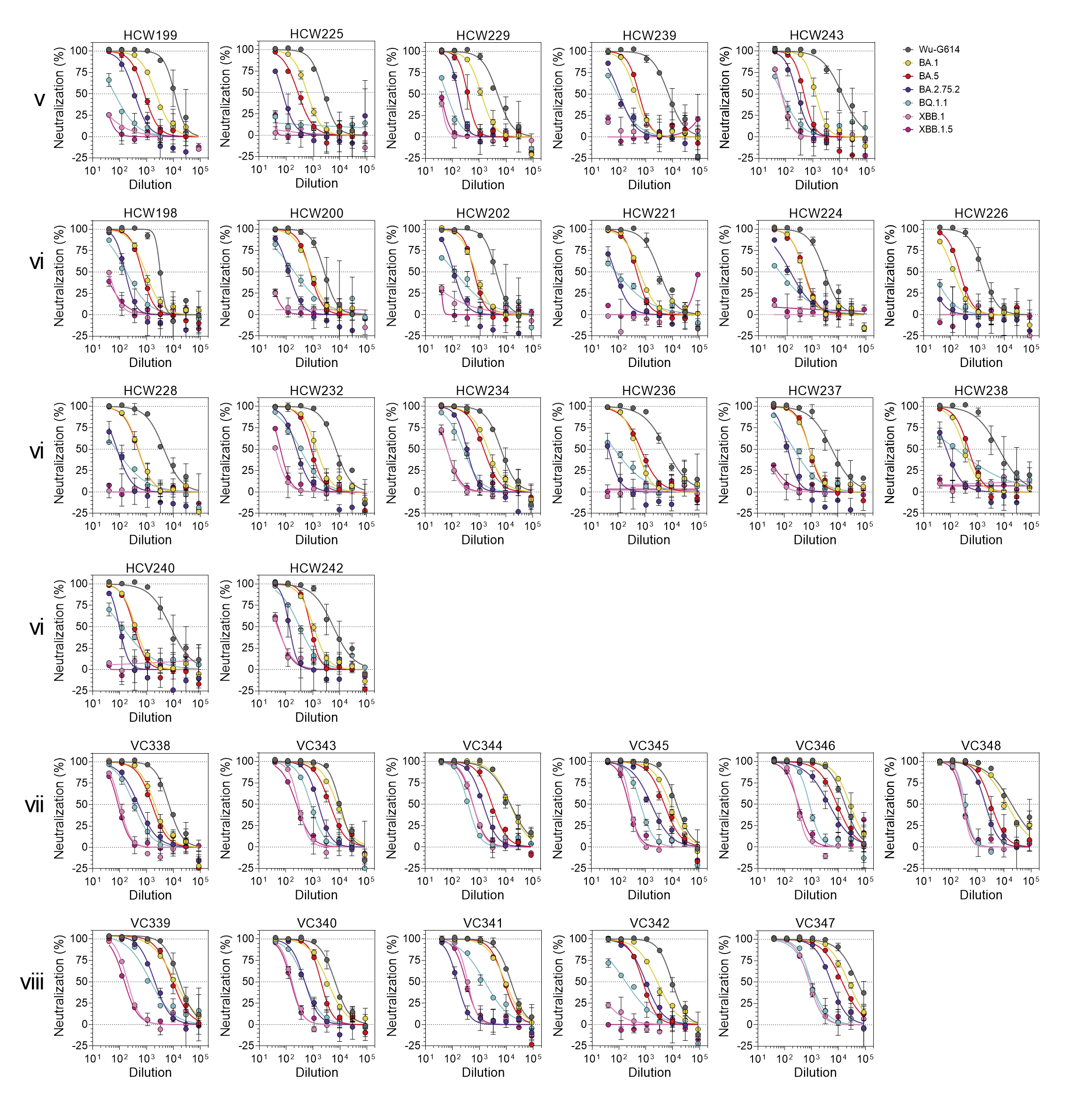
**

**Supplementary Fig. 5.**

Dose-response plasma neutralization curves for cohorts v-viii. Means ± standard deviation of two technical replicates from of one representative experiment are shown.

**
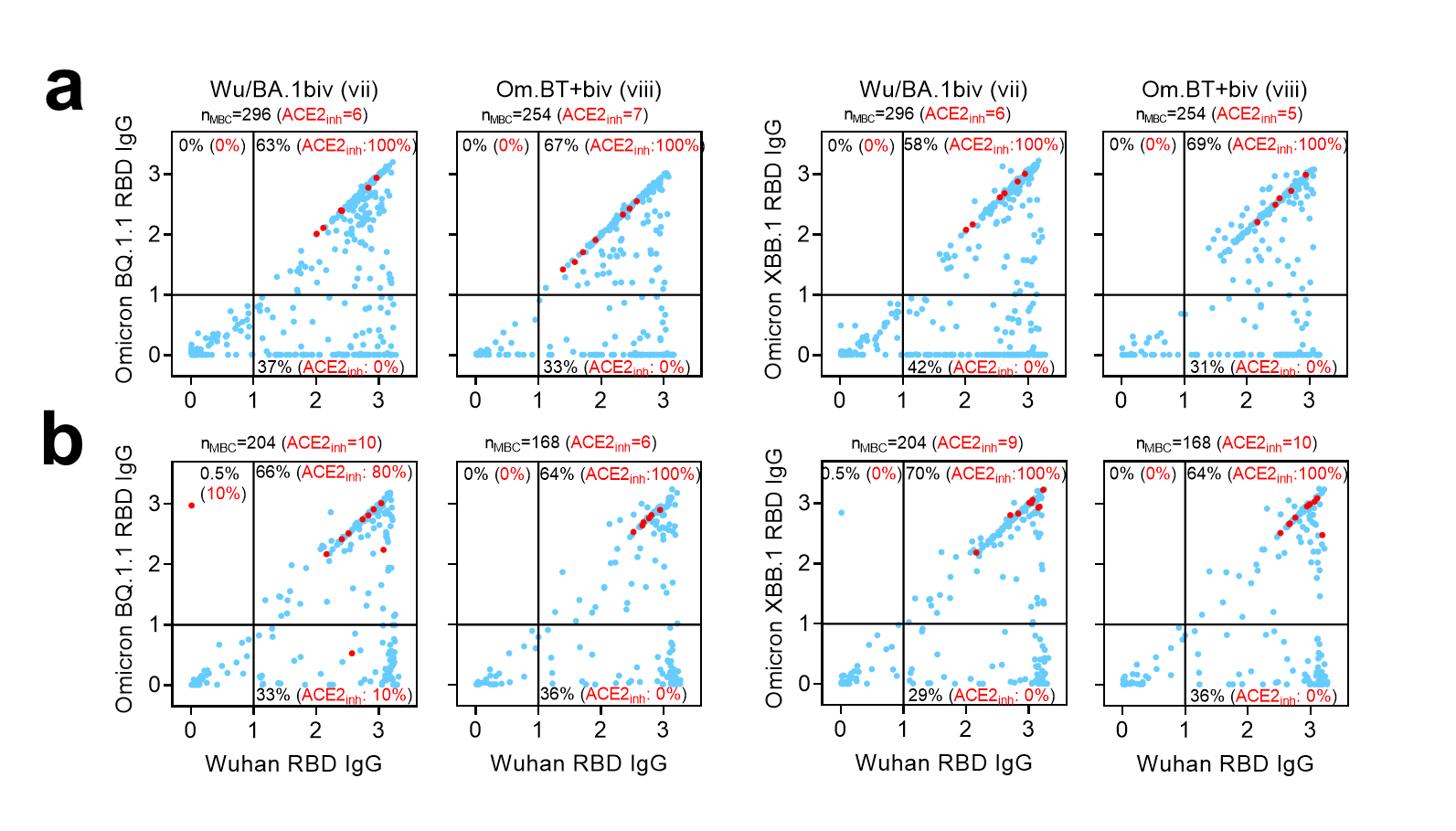
**

**Supplementary Fig. 6.** MBC-derived RBD-directed IgGs inhibiting binding of ACE2 to BQ.1.1 or to XBB.1 RBDs are depicted in red from cohorts vii and viii at 14 days (a) and 3 months (b) after last vaccination. Total and ACE2-inhibiting (ACE2inh) RBD-directed IgG positive cultures are indicated above each graph and reactivity frequencies within each quadrant.
