# Supplementary material for: Neutralization, effector function and immune imprinting of Omicron variants
Source: Nature. 2023 Aug 30;621(7979):592–601. doi: 10.1038/s41586-023-06487-6 (PMC10511321; doi:10.1038/s41586-023-06487-6)
Supplement: Supplementary file 2 — Reporting Summary [file 41586_2023_6487_MOESM2_ESM.pdf]

nature portfolio

Corresponding author(s): Diamond MS, Corti D, Veesler D

Last updated by author(s): Jun 19, 2023

Reporting Summary

Nature Portfolio wishes to improve the reproducibility of the work that we publish. This form provides structure for consistency and transparency in reporting. For further information on Nature Portfolio policies, see our Editorial Policies and the Editorial Policy Checklist.

Statistics

For all statistical analyses, confirm that the following items are present in the figure legend, table legend, main text, or Methods section.

n/a	Confirmed	
☐	☒	The exact sample size (*n*) for each experimental group/condition, given as a discrete number and unit of measurement
☐	☒	A statement on whether measurements were taken from distinct samples or whether the same sample was measured repeatedly
☐	☒	The statistical test(s) used AND whether they are one- or two-sided<br>*Only common tests should be described solely by name; describe more complex techniques in the Methods section.*
☐	☒	A description of all covariates tested
☐	☒	A description of any assumptions or corrections, such as tests of normality and adjustment for multiple comparisons
☐	☒	A full description of the statistical parameters including central tendency (e.g. means) or other basic estimates (e.g. regression coefficient) AND variation (e.g. standard deviation) or associated estimates of uncertainty (e.g. confidence intervals)
☐	☒	For null hypothesis testing, the test statistic (e.g. *F*, *t*, *r*) with confidence intervals, effect sizes, degrees of freedom and *P* value noted<br>*Give P values as exact values whenever suitable.*
☒	☐	For Bayesian analysis, information on the choice of priors and Markov chain Monte Carlo settings
☒	☐	For hierarchical and complex designs, identification of the appropriate level for tests and full reporting of outcomes
☐	☒	Estimates of effect sizes (e.g. Cohen's *d*, Pearson's *r*), indicating how they were calculated

Our web collection on statistics for biologists contains articles on many of the points above.

Software and code

Policy information about availability of computer code

Data collection	FACSDiva v9.0; Octet Data acquisition v11.1; Leginon v3.4; BioTek Gen5 Image Prime v3.11; Biacore T200 Evaluation software v.3.2
Data analysis	FlowJo 10.8.1; GraphPad Prism 9.1.0; Octet Data analysis HT v12.0; Warp (v1.0.9); Relion 3.1; cryoSPARC (v4.2.2); UCSF Chimera (v1.17.1); Coot (v0.9.6); Phenix (dev-4788); Excel for Microsoft 365 (v16); Biacore Insight software (v4.0.8.20368)

For manuscripts utilizing custom algorithms or software that are central to the research but not yet described in published literature, software must be made available to editors and reviewers. We strongly encourage code deposition in a community repository (e.g. GitHub). See the Nature Portfolio guidelines for submitting code & software for further information.

Data

Policy information about availability of data

All manuscripts must include a data availability statement. This statement should provide the following information, where applicable:

- Accession codes, unique identifiers, or web links for publicly available datasets
- A description of any restrictions on data availability
- For clinical datasets or third party data, please ensure that the statement adheres to our policy

The cryoEM maps and atomic coordinates were deposited to the EMDB and PDB with accession numbers EMD-29531 and PDB-8FXC (BQ.1.1/ACE2/S309), EMD-29530 and PDB-8FXB (XBB.1/ACE2/S309) and EMD-40240 and PDB- 8S9G (BN.1/ACE2/S309).

Human research participants

Policy information about studies involving human research participants and Sex and Gender in Research.

Reporting on sex and gender	Findings do not apply to only one sex or gender.<br>Sex and/or gender were not considered in study design.<br>Sex and/or gender was determined on self-reporting.<br>Individual sex and/or gender information is shown as coded in Table S5 and S6 for all the participants analyzed.<br>Sex and/or gender-based analyses were not performed as they are not relevant for this study.
Population characteristics	Individuals who received 3 or 4 doses of the Wuhan-Hu-1 monovalent mRNA vaccine or the Wuhan-Hu-1/BA.5 or Wuhan-Hu-1/BA.1 bivalent mRNA vaccines. Demographic data is provided in Tables S6 and S7.
Recruitment	Enrolled in the HAARVI study or under study protocols approved by the local institutional review boards. The participation to the study is voluntary so there might be a self-selection bias that cannot be eliminated. However, since this bias is present in all the cohorts analyzed, it is not expected to impact the results.
Ethics oversight	University of Washington Human Subjects Division Institutional Review Board (STUDY00000959) and Canton Ticino and Canton Aargau Ethics Committees, Switzerland.

Note that full information on the approval of the study protocol must also be provided in the manuscript.

Field-specific reporting

Please select the one below that is the best fit for your research. If you are not sure, read the appropriate sections before making your selection.

☒ Life sciences ☐ Behavioural & social sciences ☐ Ecological, evolutionary & environmental sciences

For a reference copy of the document with all sections, see nature.com/documents/nr-reporting-summary-flat.pdf

Life sciences study design

All studies must disclose on these points even when the disclosure is negative.

Sample size	For in vivo studies the sample size was chosen based on prior experience with these animal models and previous publications with human cohorts.
Data exclusions	No data were excluded from the analysis.
Replication	Experimental assays were performed at least in two independent replicates. Each replicates was performed with 2 or more technical replicates. All attempts at replication were successful
Randomization	Randomization and blinding were not possible due to pre-defined housing conditions (separated isolators between infected and non-infected animals).
Blinding	Ex vivo analyses were blinded (coded samples).

Reporting for specific materials, systems and methods

We require information from authors about some types of materials, experimental systems and methods used in many studies. Here, indicate whether each material, system or method listed is relevant to your study. If you are not sure if a list item applies to your research, read the appropriate section before selecting a response.

Materials & experimental systems

n/a	Involved in the study	
☐	☒	Antibodies
☐	☒	Eukaryotic cell lines
☒	☐	Palaeontology and archaeology
☐	☒	Animals and other organisms
☒	☐	Clinical data
☒	☐	Dual use research of concern

Methods

n/a	Involved in the study	
☒	☐	ChIP-seq
☐	☒	Flow cytometry
☒	☐	MRI-based neuroimaging

Antibodies

Antibodies used	S309 (PDB: 6WS6; produced in house); S2L20 (PDB: 7N8H; produced in house); anti-Human IgG Fc antibody, PE (Thermo Fisher; Catalog #H10500; RRID: AB_2536552; Lot #2481260); anti CD20-PECy7 (BD; Catalog #335793; Clone L27; Lot #2215050); anti-CD3-APC eFluor780 (Thermo Fisher; Catalog #47-0037-41; Clone OKT3; Lot #2470232); anti-CD8a-APC eFluor780 (Thermo FIsher; Catalog #47-0086-42; Clone OKT8; Lot #2392607); anti-CD14-APC eFluor780 (Thermo Fisher; Catalog #47-0149-42; Clone 61D3; Lot #2488593); anti-CD16-APC eFluor780 (Thermo Fisher; Catalog #47-0168-41; Clone eBioCB16; Lot #2463976); anti-CD38-BV785 (Biolegend; Catalog #303529; Clone HIT2; Lot #B357139); anti-IgD-Alexa Fluor647 (Biolegened; Catalog #348227; Clone: IA6-2; Lot #B368416); anti-IgM-Alexa Fluor647 (Biolegend; Catalog #314535; Clone MHM-88; Lot #B337906); control hE16 mAb (PMID: 17041857); Alexa Fluor647-goat anti-human IgG secondary Ab (Jackson ImmunoResearch, Catalog#109–606–098); AP-conjugated goat anti-human IgG secondary antibody (Southern Biotech, Catalog#2040-04); AP-conjugated secondary goat anti-mouse IgG-AP (Southern Biotech, Catalog#1032-815 04); S2L20 (PMID: 33761326); anti-AVI polyclonal AB (Genscript, Cat# A00674-40)
Validation	anti-Human IgG FC antibody, PE: C57BL/6 mouse splenocytes were incubated with Mouse CCL19 (MIP-3 beta) Recombinant Protein (a human IgG Fc-tagged ligand that interacts with CCR7), followed by CD4 Monoclonal Antibody, APC (Product # 17-0041-82) and staining buffer (left) or 0.5 µg of Anti-IgG Fc Secondary Antibody, PE (right). Cells in the lymphocyte gate were used for analysis.<br>anti-CD20-PECy7: Flow cytometric analysis was performed on whole blood stained with the indicated conjugated antibody. Laser excitation was at 405 nm, 488 nm, or 635 nm. Representative data analyzed with a BD FACS™ brand flow cytometer is shown in the following plots.<br>anti-CD3-APC eFluor780: Staining of human peripheral blood cells. As expected based on known relative expression patterns, CD3 clone OKT3 stains a subset of lymphocytes (T cells) and does not stain monocytes and granulocytes (middle plot). Additional analysis of lymphocytes shows that CD3 clone OKT3 does not stain any CD19+ B cells (right plot).<br>anti-CD8a-APC eFluor780: Staining of normal human peripheral blood cells with Anti-Human CD19 eFluor® 450 (Product # 48-0198-42) and staining buffer (autofluorescence) (left) or Anti-Human CD8a APC-eFluor® 780 (right). Cells in the lymphocyte gate were used for analysis.<br>anti-CD16-APC eFluor780: Staining of human peripheral blood cells. As expected based on known relative expression patterns, CD16 clone CB16 stains all granulocytes, a subset of monocytes and a subset of lymphocytes (NK cells). Details: Normal human whole blood was surface stained with CD16 (clone CB16). After staining, red blood cells were lysed using 1-step Fix/Lyse Buffer. Cells in the lymphocyte (purple histogram), monocyte (orange histogram), or granulocyte (blue histogram) gates were used for analysis.<br>anti-CD14-APC eFluor780: Staining of normal human peripheral blood cells with Mouse IgG1 K Isotype Control APC-eFluor® 780 (Product # 47-4714-82) (blue histogram) or Anti-Human CD14 APC-eFluor® 780 (purple histogram). Cells in the monocyte gate were used for analysis.<br>anti-CD38-BV785: Human peripheral blood lymphocytes were stained with CD38 (clone HIT2) Brilliant Violet 785™ (filled histogram) or mouse IgG1, κ Brilliant Violet 785™ isotype control (open histogram).<br>anti-IgD-Alexa Fluor647: Human peripheral blood lymphocytes were stained with CD19 PE and IgD (clone IA6-2) Alexa Fluor® 647 (top) or mouse IgG2a, κ Alexa Fluor® 647 isotype control (bottom).<br>anti-IgM-Alexa Fluor647: Overnight cultured human peripheral blood mononuclear cells were stained with CD19 PE and IgM (clone MHM-88) Alexa Fluor® 647 (top) or mouse IgG1, κ Alexa Fluor® 647 isotype control (bottom). Data shown was gated on lymphocyte population.<br>anti-Avi polyclonal antibody: reactivity is based on the information on manufacturer's homepage and confirmed in-house. Anti-avi polyclonal antibody was diluted to a final concentration of 25 µg/ml and immobilized on a CM5 chip surface via amine coupling.<br>anti-Human IgG Fc polyclonal antibody (goat), Alexa Fluor647: staining of ExpiCHO-S cells expressing SARS-CoV-2 S variants. Alexa Fluor647-labelled Goat anti-human IgG secondary Ab was prepared at 2 µg/mL and added onto ExpiCHO-S cells after two washing steps. Cells were then washed twice and resuspended in wash buffer for data acquisition at Ze5 cytometer. Manufacturer's website suggested 1:100 to 1:800 working dilution.<br>anti-Human IgG Fc polyclonal antibody (goat), AP: used for ELISA to measure binding of mAbs and MBC-derived Abs to RBD-coated plates. This Ab is added to ELISA plates (1/500 dilution, as routinely used in the lab to increase sensitivity of the ELISA) after 4 washing steps with PBS 0.05% Tween 20 (PBS-T) and incubated for 1 h at room temperature. Plates are then washed four times with PBS-T and 4-nitrophenyl phosphate substrate was added.<br>We described S309, hE16 and S2L20 monoclonal antibodies in the indicated studies. Target validation of these antibodies was performed with multiple binding assays and structural analyses.<br>Reactivity of secondary antibodies listed above is based on the information on manufacturer's homepages.

Eukaryotic cell lines

Policy information about cell lines and Sex and Gender in Research

Cell line source(s)	Cell lines used in this study were obtained from ATCC (HEK293T and VeroE6), Thermo Fisher Scientific (ExpiCHO-S cells, FreeStyle 293-F cells and Expi293F cells), Takara (Lenti-X 293T cells), kindly gifted from Jesse Bloom (HEK293T-ACE2), or generated in-house (VeroE6-TMPRSS2, BHK-21-GFP1-10 or VeroE6-TMPRSS2-GFP11).
Authentication	None of the cells lines used were authenticated.
Mycoplasma contamination	Cells lines were not tested for mycoplasma contamination.
Commonly misidentified lines<br>(See ICLAC register)	No commonly misidentified cell lines were used in the study.

Animals and other research organisms

Policy information about studies involving animals; ARRIVE guidelines recommended for reporting animal research, and Sex and Gender in Research

Laboratory animals	Heterozygous K18-hACE2 C57BL/6 J mice (strain: 2B6.Cg-Tg(K18-ACE2)2Prlmn/J; eight- to ten-week-old) were obtained from The Jackson Laboratory.<br>Golden Syrian hamsters (Mesocricetus auratus; RjHan:AURA) of 5-6 weeks of age were purchased from Janvier Laboratories.
Wild animals	The study did not involve wild animals
Reporting on sex	Only female mice were used for reproducibility of the model which was set up with female animals.<br>Only male hamsters were used.
Field-collected samples	No field collected samples were used in the study
Ethics oversight	Animal studies were carried out in accordance with the recommendations in the Guide for the are and Use of Laboratory Animals of the National Institutes of Health. The protocols were approved by the Institutional Animal Care and Use Committee at the Washington University School of Medicine (assurance number A3381–01) or the Animal Experimentation Ethics Committee (CETEA 89) of the Institut Pasteur

Note that full information on the approval of the study protocol must also be provided in the manuscript.

Flow Cytometry

Plots

Confirm that:

☒ The axis labels state the marker and fluorochrome used (e.g. CD4-FITC).

☒ The axis scales are clearly visible. Include numbers along axes only for bottom left plot of group (a 'group' is an analysis of identical markers).

☒ All plots are contour plots with outliers or pseudocolor plots.

☒ A numerical value for number of cells or percentage (with statistics) is provided.

Methodology

Sample preparation	PBMC Analysis: Frozen PMBCs were thawed on ice, collected by centrifugation at 1,000 x g for 5 mins at 4C and washed twice with PBS. The cells were then stained with Zombie Aqua dye (Biolegend; diluted 1:100 in PBS) for 30 mins at room temperature after which the cells were washed twice with FACS staining buffer (0.1% BSA, 0.1% NaN3 in PBS). The cells were then stained with antibodies for CD20-PECy7 (BD), CD3-Alexa eFluor780 (ThermoFisher), CD8-Alexa eFluor780 (ThermoFisher), CD14-Alexa eFluor780 (ThermoFisher), CD16-Alexa eFluor780 (ThermoFisher), IgM-Alexa Fluor647 (BioLegend), IgD-Alexa Fluor647 (BioLegend), and CD38-Brilliant Violet 785 (BioLegend), all diluted 1:200 in Brilliant Stain Buffer (BD), along with the RBD-streptavidin tetramers (Wuhan-Hu-1 RBD-Dylight488; BA.1/2/5 RBD-BV421; BQ.1.1 RBD-Alexa568; Streptavidin-biotin-BV711) for 30 mins at 4C. The cells were washed three times, resuspended in FACS staining buffer, and passed through a 35 µm filter.<br><br>Spike Expression: Transiently transfected BHK-21-GFP1-10 cells were collected by centrifugation at 1,000 x g for 5 min. The cells were washed once with PBS and fixed with 2% paraformaldehyde. The cells were washed twice with flow staining buffer (1% BSA, 1 mM EDTA, 0.1% NaN3 in PBS) and labeled with 250 µg/mL of S2L20, an NTD-directed antibody that recognizes all currently and previously circulating SARS-CoV-2 variants, for 45 minutes. The cells were washed three times with flow staining buffer and labeled with a PE-conjugated anti-Human IgG Fc antibody (Thermo Fisher) for 30 mins. The cells were washed an additional three times and resuspended in flow staining buffer.
Instrument	BD FACSAria III; BD FACSymphony A3
Software	FACS Diva v9.0; FlowJo 10.8.1
Cell population abundance	The frequency and counts of memory B cells are provided in Extended Data Fig9.
Gating strategy	PBMC Analysis: Lymphocytes were selected based on SSC-A vs FSC-A. Single cells were selected based on FSC-H vs FSC-A. Live cells were then selected based on FSC-A vs Zombie-Aqua with live cells being Aqua negative/low. B cells were then selected based on CD20-PECy7 vs CD3/8/14/16-Alexa eFluor780 with the positive population being PECy7 high and CD3/8/14/16 negative. Memory B cells were selected based on CD38-BV785 vs IgD/M-Alexa647 with memory B cells being BV785 negative/low and Alexa647 negative/low. Memory B cells binding streptavidin-biotin were selected based on FSC-A vs Streptavidin-biotin-BV711 with cells not binding streptavidin-biotin (BV711 negative) being selected. RBD-binding memory B cells were identified based on Omicron RBD pool-BV421 vs Wuhan-Hu-1 RBD-Dylight 488 with BV421 and/or Dylight 488-positive cells being selected. Final quadrant gates were set on the RBD-positive memory B cells to determine specificity based on Omicron RBD pool-BV421 vs Wuhan-Hu-1 RBD-Dylight 488 or BQ.1.1 RBD-Alexa568 vs Omicron RBD pool-BV421.

Spike Expression: Cells were selected using SSC-A vs FSC-A and single cells were selected using FSC-H vs FSC-A. Spike expression was measured by PE intensity, using the mock transfected cells to establish the spike-negative population.

☒ Tick this box to confirm that a figure exemplifying the gating strategy is provided in the Supplementary Information.
