# Supplementary material for: Neutralization, effector function and immune imprinting of Omicron variants
Source: Nature. 2023 Aug 30;621(7979):592–601. doi: 10.1038/s41586-023-06487-6 (PMC10511321; doi:10.1038/s41586-023-06487-6)
Supplement: Supplementary file 3 — This file contains Supplementary Tables 1-8. [file 41586_2023_6487_MOESM3_ESM.pdf]

Supplementary Table 1. Kinetics of monomeric human ACE2 binding to immobilized SARS-CoV-2 variant RBDs as measured by biolayer interferometry.

	**K_D (nM)**	**k_{on} ($M^{-1}s^{-1}$)**	**k_{off}(s^{-1})**
**Wu**	101.1 ± 7.3	1.30×10^5	1.31×10^{-2}
**BA.4/5**	12.8 ± 2.2	1.45×10^5	1.82×10^{-3}
**BA.2.75.2**	26.2 ± 1.7	1.35×10^5	3.52×10^{-3}
**BQ.1.1**	13.7 ± 1.4	1.39×10^5	1.89×10^{-3}
**XBB.1**	88.4 ± 11.9	1.47×10^5	1.29×10^{-2}
**XBB.1.5**	26.8 ± 5.0	1.20×10^5	3.14×10^{-3}

Values are presented as mean ± standard deviations obtained from 2-3 batches of each RBD and ACE2.

Supplementary Table 2. Kinetics of monomeric human ACE2 binding to immobilized SARS-CoV-2 variant RBDs as measured by surface plasmon resonance.

RBD	Average k_{on} (1/Ms)	stdev(ka)	Average k_{off} (1/s)	stdev(kd)	Average K_D(nM)	stdev(K_D)	# of replicates
Wu	7.18E+04	6.39E+03	6.19E-03	1.62E-04	86.91	8.82	7
Wu-E340A	5.66E+04	8.96E+03	6.01E-03	8.01E-04	106.71	4.78	6
BQ.1.1	4.22E+04	7.97E+03	7.91E-04	2.08E-05	19.26	3.21	6
BA.2.75.2	3.53E+04	7.28E+02	1.50E-03	5.43E-05	42.38	1.32	3
BA.4/5	3.31E+04	3.97E+03	9.32E-04	1.30E-04	28.22	2.74	8
XBB	4.48E+04	8.15E+03	4.19E-03	8.52E-04	93.48	4.72	3
XBB.1.5	5.58E+04	1.20E+03	1.48E-03	2.45E-05	26.58	0.16	4

Supplementary Table 3. Statistically significant differences of mean neutralization titers.

**Fig. 3a**	**Mean rank diff.**	**Significant?**	**Adjusted P Value**
Wu-D614 vs. BA.2	-40.4	***	<0.001
Wu-D614 vs. BQ.1	-44.9	***	<0.001
Wu-D614 vs. BQ.1.1	-61.5	***	<0.001
Wu-D614 vs. BF.7	-56.5	***	<0.001
Wu-D614 vs. BN.1	-67	***	<0.001
**Fig. 3b**	**Mean rank diff.**	**Significant?**	**Adjusted P Value**
Wuhan vs. BA.2	-48.4	Yes	<0.001
Wuhan vs. BQ.1	-35.1	Yes	0.01
Wuhan vs. BQ.1.1	-43.1	Yes	<0.001
Wuhan vs. BN.1	-56.3	Yes	<0.001
Wuhan vs. E340A	-60.3	Yes	<0.001
**Fig. 3d**	**Mean rank diff.**	**Significant?**	**Adjusted P Value**
sotrovimab vs. S309-GRLR	35.2	*	0.04
sotrovimab vs. S309-GRLR	42.2	**	0.003
sotrovimab vs. S309-GRLR	40.7	*	0.02
**Fig. 3d**	**Mean rank diff.**	**Significant?**	**Adjusted P Value**
sotrovimab vs. S309-GRLR	40.3	**	0.007
sotrovimab vs. S309-GRLR	37	*	0.02
sotrovimab vs. S309-GRLR	34.6	*	0.04
sotrovimab vs. S309-GRLR	48.3	**	0.009
sotrovimab vs. S309-GRLR	41.5	*	0.05
sotrovimab vs. sotrovimab	53.8	**	0.002
sotrovimab vs. S309-GRLR	48.8	**	0.008

Supplementary Table 4. Kinetics of S309 Fab binding to immobilized SARS-CoV-2 variant RBDs as measured by surface plasmon resonance.

RBD	average k_{on} (1/Ms)	stdev(k_{on})	average k_{off} (1/s)	stdev (k_{off})	average K_D (nM)	stdev(K_D)	number of replicates
Wu	8.86E+04	8.76E+03	1.38E-05	1.05E-05	0.20	0.12	12
Wu-E340A	NB	N/A	NB	N/A	NB	N/A	6
Delta (His avi)	8.42E+04	1.02E+04	2.56E-05	4.89E-06	0.33	0.08	4
BA.1	2.21E+04	2.27E+03	2.48E-04	2.54E-05	11.35	2.08	6
BA.2	2.16E+04	2.01E+03	7.05E-04	5.25E-05	32.88	2.79	8
BA.2.75.2	2.55E+04	3.14E+03	2.80E-05	5.52E-06	0.86	0.25	6
BQ.1	2.51E+04	1.39E+03	4.40E-04	3.60E-05	17.63	2.31	5
BQ.1.1	2.27E+04	1.67E+03	6.43E-04	7.37E-05	28.47	3.89	8
XBB	2.57E+04	2.24E+03	4.12E-05	2.00E-05	1.56	0.74	6
XBB.1.5	2.76E+04	2.41E+03	4.45E-05	3.01E-06	1.61	0.08	8
CH.1.1	3.33E+04	6.40E+02	1.67E-05	4.56E-07	0.50	0.02	4
BN.1	8.92E+02	1.14E+02	6.85E-05	3.08E-05	74.70	28.01	5
BN.1 + PNGase F	1.50E+03	1.97E+02	2.59E-04	3.58E-06	174.4	20.5	2
BN.1-T356K	2.09E+04	8.70E+00	4.50E-05	4.27E-06	2.16	0.2	2

NB: no binding; N/A: not applicable.

Supplementary Table 5. Donors' demographics.

Donor ID	Gender	Age	Vaccine doses	COVID diagnosis	SARS-CoV-2 variant	Δ sample-vaccine (days)	Cohort
**8H**	M	79	4	no	/	72	Wu$_4$vacc
**15H**	M	52	4	no	/	5	Wu$_4$vacc
**17H**	M	76	4	no	/	21	Wu$_4$vacc
**25H**	M	60	4	no	/	16	Wu$_4$vacc
**27H**	M	59	4	no	/	42	Wu$_4$vacc
**30H - 4X**	F	61	4	no	/	17	Wu$_4$vacc
**46H**	M	60	4	no	/	15	Wu$_4$vacc
**69H**	F	68	4	no	/	97	Wu$_4$vacc
**71H - 4x**	F	58	4	no	/	48	Wu$_4$vacc
**76H - 4x**	F	38	4	no	/	13	Wu$_4$vacc
**12H**	F	42	4	no	/	34	Wu/BA.5biv
**16H**	F	46	4	no	/	33	Wu/BA.5biv
**17H**	M	76	5	no	/	18	Wu/BA.5biv
**29H**	M	64	5	no	/	37	Wu/BA.5biv
**30H - 5x**	F	61	5	no	/	30	Wu/BA.5biv
**31H***	F	/	3	no	/	54	Wu/BA.5biv
**46H**	M	60	5	no	/	16	Wu/BA.5biv
**51H**	F	50	4	no	/	33	Wu/BA.5biv
**54H**	F	34	4	no	/	27	Wu/BA.5biv
**57H**	M	36	5	no	/	46	Wu/BA.5biv
**69H**	F	68	5	no	/	42	Wu/BA.5biv
**71H - 5x**	F	58	5	no	/	20	Wu/BA.5biv
**76H - 5x**	F	38	5	no	/	60	Wu/BA.5biv
**110C**	M	51	5	yes	WA-1	38	preOm+biv
**245C**	F	28	4	yes	WA-1	25	preOm+biv
**63C**	F	36	4	yes	WA-1	33	preOm+biv
**71C**	F	69	5	yes	WA-1	28	preOm+biv
**87C**	M	76	5	yes	WA-1	30	preOm+biv
**269C**	M	41	4	yes	Gamma/P.1 & Om. BA.1	30	Om.BT+biv
**318C**	F	/	4	yes	Omicron	42	Om.BT+biv
**319C**	M	/	4	yes	Omicron	28	Om.BT+biv
**324C**	F	24	4	yes	Omicron	32	Om.BT+biv
**331C**	M	25	5	yes	Omicron	33	Om.BT+biv
**345C**	M	/	4	yes	Omicron	28	Om.BT+biv
**388C**	M	34	4	yes	Omicron BA.5	45	Om.BT+biv
**392C**	M	/	6	yes	Omicron BA.5	40	Om.BT+biv
**400C**	F	21	4	yes	Omicron BA.2	22	Om.BT+biv
**403C**	F	21	4	yes	Omicron BA.2.12.1	33	Om.BT+biv
**407C**	F	33	4	yes	Omicron BA.2	34	Om.BT+biv
**414C**	M	/	4	yes	Omicron BA.4	51	Om.BT+biv
**415C**	F	/	4	yes	Omicron BA.2	30	Om.BT+biv
**422C**	F	20	4	yes	Omicron BA.2	27	Om.BT+biv
**424C**	F	21	4	yes	Omicron BA.2.12.1	29	Om.BT+biv
**425C**	M	45	4	yes	Omicron BA.2.12.1	31	Om.BT+biv
**428C**	F	22	4	yes	Omicron BA.5	13	Om.BT+biv
**445C**	F	/	4	yes	Omicron BA.5	36	Om.BT+biv
**56H**	M	46	4	yes	Omicron[&]	28	Om.BT+biv
**HCW199**	M	67	3	no	/	18	Wu$_3$vacc
**HCW225**	F	50	3	no	/	25	Wu$_3$vacc
**HCW229**	F	45	3	no	/	20	Wu$_3$vacc
**HCW239**	F	33	3	no	/	26	Wu$_3$vacc
**HCW243**	F	29	3	no	/	27	Wu$_3$vacc
**HCW198**	M	52	3	yes	pre Omicron	18	preOm+vacc
**HCW200**	F	41	3	yes	pre Omicron	13	preOm+vacc
**HCW202**	M	46	3	yes	pre Omicron	13	preOm+vacc
**HCW221**	F	39	3	yes	pre Omicron	20	preOm+vacc
**HCW224**	F	49	3	yes	pre Omicron	18	preOm+vacc
**HCW226**	F	48	3	yes	pre Omicron	25	preOm+vacc
**HCW228**	M	35	3	yes	pre Omicron	20	preOm+vacc
**HCW232**	F	32	3	yes	pre Omicron	25	preOm+vacc
**HCW234**	M	27	3	yes	pre Omicron	19	preOm+vacc
**HCW236**	F	34	3	yes	pre Omicron	27	preOm+vacc
**HCW237**	F	45	3	yes	pre Omicron	20	preOm+vacc
**HCW238**	M	27	3	yes	pre Omicron	26	preOm+vacc

**HCW240**	F	30	3	yes	pre Omicron	26	preOm+vacc
**HCW242**	F	30	3	yes	pre Omicron	25	preOm+vacc
**VC338**	F	34	4	no	/	13/82	Wu/BA.1biv
**VC343**	F	25	4	no	/	14/83	Wu/BA.1biv
**VC344**	M	27	4	no	/	13/83	Wu/BA.1biv
**VC345**	M	53	4	no	/	14/82	Wu/BA.1biv
**VC346**	M	57	4	no	/	14/82	Wu/BA.1biv
**VC348**	M	30	4	no	/	14/82	Wu/BA.1biv
**VC349**	F	48	4	no	/	33/63	Wu/BA.1biv
**VC339**	F	45	4	yes	Omicron BA.1/BA.2	14/84	Om.BT+biv
**VC340**	F	36	4	yes	Omicron BA.1/BA.2	14/83	Om.BT+biv
**VC341**	F	31	4	yes	Omicron BA.1/BA.2	14/83	Om.BT+biv
**VC342**	F	28	4	yes	Omicron BA.1/BA.2	14/83	Om.BT+biv
**VC347**	M	37	4	yes	Omicron BA.1/BA.2	14/81	Om.BT+biv

[§]TP, timepoint. *Donor 31H received the Janssen COVID-19 vaccination as the primary vaccine series.
[&]determined by longitudinal N ELISA.

Supplementary Table 6. Kidney transplant recipients' and healthcare workers' demographics.

Donor ID	Gender	Age	No. of immuno-suppressive drugs*	Vaccine doses	COVID diagnosis	SARS-CoV-2 variant	Δ sample-vaccine (days)	Cohort
KTR-004	M	68	2	4	no	/	83	KTR Wu$_4$vacc
KTR-007	M	77	3	4	no	/	140	KTR Wu$_4$vacc
KTR-009	M	72	2	4	no	/	46	KTR Wu$_4$vacc
KTR-010	F	70	3	4	no	/	51	KTR Wu$_4$vacc
KTR-011	M	56	2	4	no	/	80	KTR Wu$_4$vacc
KTR-013	F	46	1	4	no	/	92	KTR Wu$_4$vacc
KTR-026	M	37	2	4	no	/	63	KTR Wu$_4$vacc
KTR-027	F	66	3	4	no	/	35	KTR Wu$_4$vacc
KTR-030	M	58	2	4	no	/	71	KTR Wu$_4$vacc
KTR-039	M	66	2	4	no	/	77	KTR Wu$_4$vacc
KTR-042	M	78	3	4	no	/	40	KTR Wu$_4$vacc
KTR-047	M	52	2	4	no	/	76	KTR Wu$_4$vacc
KTR-050	M	34	2	4	no	/	84	KTR Wu$_4$vacc
KTR-054	M	70	2	4	no	/	18	KTR Wu$_4$vacc
KTR-056	M	74	2	4	no	/	70	KTR Wu$_4$vacc
KTR-059	M	63	2	4	no	/	78	KTR Wu$_4$vacc
KTR-060	M	50	2	4	no	/	71	KTR Wu$_4$vacc
KTR-061	M	71	2	4	no	/	54	KTR Wu$_4$vacc
KTR-071	F	59	3	4	no	/	35	KTR Wu$_4$vacc
KTR-083	M	71	3	4	no	/	82	KTR Wu$_4$vacc
KTR-085	F	59	2	4	no	/	71	KTR Wu$_4$vacc
KTR-094	M	66	2	4	no	/	41	KTR Wu$_4$vacc
KTR-095	M	63	3	4	no	/	74	KTR Wu$_4$vacc
KTR-096	M	62	2	4	no	/	43	KTR Wu$_4$vacc
KTR-101	M	61	2	4	no	/	72	KTR Wu$_4$vacc
KTR-102	M	72	3	4	no	/	133	KTR Wu$_4$vacc
KTR-017	F	81	2	4	yes	pre Omicron	58	KTR preOm+Wu$_4$vacc
KTR-021	M	45	2	4	yes	pre Omicron	84	KTR preOm+Wu$_4$vacc
KTR-031	F	77	2	4	yes	pre Omicron	59	KTR preOm+Wu$_4$vacc
KTR-084	F	24	3	4	yes	pre Omicron	65	KTR preOm+Wu$_4$vacc
KTR-099	M	52	2	4	yes	pre Omicron	93	KTR preOm+Wu$_4$vacc
HCW-001	M	52	/	3	no	/	90	HCW Wu$_3$vacc
HCW-002	F	44	/	3	no	/	78	HCW Wu$_3$vacc
HCW-003	F	41	/	3	no	/	76	HCW Wu$_3$vacc
HCW-004	M	48	/	3	no	/	90	HCW Wu$_3$vacc
HCW-005	F	57	/	3	no	/	83	HCW Wu$_3$vacc
HCW-008	F	48	/	3	no	/	86	HCW Wu$_3$vacc
HCW-009	F	54	/	3	no	/	81	HCW Wu$_3$vacc
HCW-011	M	69	/	3	no	/	93	HCW Wu$_3$vacc
HCW-012	F	60	/	3	no	/	94	HCW Wu$_3$vacc
HCW-013	F	43	/	3	no	/	55	HCW Wu$_3$vacc
HCW-016	F	62	/	3	no	/	6	HCW Wu$_3$vacc
HCW-017	F	33	/	3	no	/	50	HCW Wu$_3$vacc
HCW-018	M	33	/	3	no	/	110	HCW Wu$_3$vacc
HCW-019	F	62	/	3	no	/	29	HCW Wu$_3$vacc
HCW-020	F	64	/	3	no	/	90	HCW Wu$_3$vacc
HCW-021	M	44	/	3	no	/	28	HCW Wu$_3$vacc
HCW-022	F	44	/	3	no	/	28	HCW Wu$_3$vacc
HCW-023	F	46	/	3	no	/	22	HCW Wu$_3$vacc
HCW-024	M	63	/	3	no	/	35	HCW Wu$_3$vacc
HCW-025	F	54	/	3	no	/	94	HCW Wu$_3$vacc
HCW-026	F	63	/	3	no	/	28	HCW Wu$_3$vacc
HCW-027	F	49	/	3	no	/	38	HCW Wu$_3$vacc
HCW-028	F	56	/	3	no	/	29	HCW Wu$_3$vacc
HCW-031	F	58	/	3	no	/	35	HCW Wu$_3$vacc
HCW-033	F	59	/	3	no	/	31	HCW Wu$_3$vacc
HCW-037	F	59	/	3	no	/	45	HCW Wu$_3$vacc
HCW-038	F	22	/	3	no	/	61	HCW Wu$_3$vacc
HCW-039	F	60	/	3	no	/	23	HCW Wu$_3$vacc
HCW-010	F	39	/	3	yes	pre Omicron	69	HCW preOm+Wu$_3$vacc
HCW-014	F	29	/	3	yes	pre Omicron	89	HCW preOm+Wu$_3$vacc
HCW-015	F	36	/	3	yes	pre Omicron	86	HCW preOm+Wu$_3$vacc
HCW-029	F	49	/	3	yes	pre Omicron	77	HCW preOm+Wu$_3$vacc
HCW-030	F	56	/	3	yes	pre Omicron	35	HCW preOm+Wu$_3$vacc
HCW-034	F	38	/	3	yes	pre Omicron	28	HCW preOm+Wu$_3$vacc
HCW-036	F	49	/	3	yes	pre Omicron	34	HCW preOm+Wu$_3$vacc

*any of the following: cyclosporin, tacrolimus, MMF/MPA, azathioprine, everolimus/sirolimus, belatacept or glucocorticoids

Supplementary Table 7. Statistically significant differences of mean neutralization and binding titers within and between cohorts

Fig. 4a	Mean rank diff.	Significant?	Adjusted P Value	Cohort
Wu-G614 vs. BQ.1.1	169	*	0.03	Wu4vacc (i)
Wu-G614 vs. XBB.1	192	**	0.003	Wu4vacc (i)
Wu-G614 vs. XBB.1.5	187	**	0.004	Wu4vacc (i)
BA.1 vs. BA.1	-145	*	0.04	Wu4vacc (i)/Om.BT+biv (iv)
BA.5 vs. BA.5	-148	*	0.03	Wu4vacc (i)/Om.BT+biv (iv)
BA.2.75.2 vs. BA.2.75.2	-151	*	0.02	Wu4vacc (i)/Om.BT+biv (iv)
Wu-G614 vs. BA.2.75.2	148	*	0.03	Wu/BA.5biv (ii)
Wu-G614 vs. BQ.1.1	147	*	0.03	Wu/BA.5biv (ii)
Wu-G614 vs. XBB.1	202	***	<0.001	Wu/BA.5biv (ii)
Wu-G614 vs. XBB.1.5	198	***	<0.001	Wu/BA.5biv (ii)
BA.1 vs. XBB.1	148	*	0.03	Wu/BA.5biv (ii)
BA.1 vs. XBB.1.5	144	*	0.04	Wu/BA.5biv (ii)
Wu-G614 vs. BQ.1.1	129	*	0.01	Om.BT+biv (iv)
Wu-G614 vs. XBB.1	162	***	<0.001	Om.BT+biv (iv)
Wu-G614 vs. XBB.1.5	152	***	<0.001	Om.BT+biv (iv)
BA.1 vs. BQ.1.1	129	*	0.01	Om.BT+biv (iv)
BA.1 vs. XBB.1	162	***	<0.001	Om.BT+biv (iv)
BA.1 vs. XBB.1.5	152	***	<0.001	Om.BT+biv (iv)
BA.5 vs. XBB.1	149	***	<0.001	Om.BT+biv (iv)
BA.5 vs. XBB.1.5	139	**	0.002	Om.BT+biv (iv)
**Fig.4b**	**Mean rank diff.**	**Significant?**	**Adjusted P Value**	**Cohort**
Wu-G614 vs. XBB.1	159	*	0.01	Wu3vacc (v)
Wu-G614 vs. XBB.1.5	161	*	0.01	Wu3vacc (v)
Wu-G614 vs. BA.2.75.2	114	***	<0.001	preOm+vacc (vi)
Wu-G614 vs. BQ.1.1	108	***	<0.001	preOm+vacc (vi)
Wu-G614 vs. XBB.1	151	***	<0.001	preOm+vacc (vi)
Wu-G614 vs. XBB.1.5	151	***	<0.001	preOm+vacc (vi)
BA.1 vs. XBB.1	99.6	**	0.005	preOm+vacc (vi)
BA.1 vs. XBB.1.5	98.9	**	0.006	preOm+vacc (vi)
BA.5 vs. XBB.1	97	**	0.009	preOm+vacc (vi)
BA.5 vs. XBB.1.5	96.3	*	0.01	preOm+vacc (vi)
**Fig. 4c RBD**	**Mean rank diff.**	**Significant?**	**Adjusted P Value**	**Cohort**
Wu-G614 vs. BA.1	108	***	<0.001	preOm+vacc (vi)
Wu-G614 vs. BA.5	94.9	***	<0.001	preOm+vacc (vi)
Wu-G614 vs. BQ.1.1	90.9	**	0.001	preOm+vacc (vi)
Wu-G614 vs. XBB.1	90.8	**	0.001	preOm+vacc (vi)
**Fig. 4c S**	**Mean rank diff.**	**Significant?**	**Adjusted P Value**	**Cohort**
BA.1 vs. BA.1	-112	*	0.04	preOm+vacc (vi)/Wu/BA.5biv (ii)
BA.2.75.2 vs. BA.2.75.2	-116	*	0.02	preOm+vacc (vi)/Wu/BA.5biv (ii)
BQ.1.1 vs. BQ.1.1	-117	*	0.02	preOm+vacc (vi)/Wu/BA.5biv (ii)
XBB.1 vs. XBB.1	-113	*	0.04	preOm+vacc (vi)/Wu/BA.5biv (ii)
**Fig. 4d**	**Mean rank diff.**	**Significant?**	**Adjusted P Value**	**Cohort**
		no		

*** (p-value < 0.001); ** (p < 0.002), * (p < 0.033) Kruskal-Wallis rank test and corrected with Dunn's test

Supplementary Table 8. Statistically significant differences of mean neutralization and binding titers within and between cohorts

ED Fig. 7a (neutralization)	Mean rank diff.	Significant?	Adjusted P Value	Cohort
Wu-G614 vs. BA.5	127	**	0.004	DP Wu4vacc
Wu-G614 vs. BA.2.75.2	194	***	<0.001	DP Wu4vacc
Wu-G614 vs. BQ.1.1	224	***	<0.001	DP Wu4vacc
Wu-G614 vs. XBB.1	229	***	<0.001	DP Wu4vacc
Wu-G614 vs. XBB.1.5	238	***	<0.001	DP Wu4vacc
BA.1 vs. BA.2.75.2	113	*	0.03	DP Wu4vacc
BA.1 vs. BQ.1.1	144	***	<0.001	DP Wu4vacc
BA.1 vs. XBB.1	148	***	<0.001	DP Wu4vacc
BA.1 vs. XBB.1.5	157	***	<0.001	DP Wu4vacc
BA.5 vs. XBB.1.5	111	*	0.03	DP Wu4vacc
Wu-G614 vs. BA.2.75.2	156	***	<0.001	HCW Wu3/4vacc
Wu-G614 vs. BQ.1.1	179	***	<0.001	HCW Wu3/4vacc
Wu-G614 vs. XBB.1	261	***	<0.001	HCW Wu3/4vacc
Wu-G614 vs. XBB.1.5	262	***	<0.001	HCW Wu3/4vacc
BA.1 vs. BQ.1.1	121	**	0.009	HCW Wu3/4vacc
BA.1 vs. XBB.1	203	***	<0.001	HCW Wu3/4vacc
BA.1 vs. XBB.1.5	204	***	<0.001	HCW Wu3/4vacc
BA.5 vs. XBB.1	185	***	<0.001	HCW Wu3/4vacc
BA.5 vs. XBB.1.5	186	***	<0.001	HCW Wu3/4vacc
**ED Fig. 7a (binding)**	**Mean rank diff.**	**Significant?**	**Adjusted P Value**	**Cohort**
Wu-G614 vs. BA.5	111	**	0.004	DP Wu4vacc
Wu-G614 vs. BQ.1.1	98.9	*	0.02	DP Wu4vacc
Wu-G614 vs. XBB.1	103	*	0.01	DP Wu4vacc
Wu-G614 vs. BA.1	191	***	<0.001	HCW Wu3/4vacc
Wu-G614 vs. BA.5	162	***	<0.001	HCW Wu3/4vacc
Wu-G614 vs. BQ.1.1	147	***	<0.001	HCW Wu3/4vacc
Wu-G614 vs. XBB.1	146	***	<0.001	HCW Wu3/4vacc
BA.1 vs. BA.2.75.2	-116	**	0.002	HCW Wu3/4vacc
**ED Fig. 7b (neutralization)**	**Mean rank diff.**	**Significant?**	**Adjusted P Value**	**Cohort**
Wu-G614 vs. Wu-G614	-208	***	<0.001	KTR Wu4vacc / HCW Wu3vacc
Wu-G614 vs. Wu-G614	-224	**	0.003	KTR Wu4vacc / HCW preOm+Wu3vacc
BA.1 vs. BA.1	-217	***	<0.001	KTR Wu4vacc / HCW Wu3vacc
BA.1 vs. BA.1	-247	***	<0.001	KTR Wu4vacc / HCW preOm+Wu3vacc
BA.5 vs. BA.5	-178	***	<0.001	KTR Wu4vacc / HCW Wu3vacc
BA.5 vs. BA.5	-220	**	0.004	KTR Wu4vacc / HCW preOm+Wu3vacc
Wu-G614 vs. BA.2.75.2	238	***	<0.001	HCW Wu3vacc

Wu-G614 vs. BQ.1.1	235	***	<0.001	HCW Wu3vacc
Wu-G614 vs. XBB.1	272	***	<0.001	HCW Wu3vacc
Wu-G614 vs. XBB.1.5	283	***	<0.001	HCW Wu3vacc
BA.1 vs. BA.2.75.2	185	***	<0.001	HCW Wu3vacc
BA.1 vs. BQ.1.1	182	***	<0.001	HCW Wu3vacc
BA.1 vs. XBB.1	219	***	<0.001	HCW Wu3vacc
BA.1 vs. XBB.1.5	230	***	<0.001	HCW Wu3vacc
BA.5 vs. BA.2.75.2	146	**	0.001	HCW Wu3vacc
BA.5 vs. BQ.1.1	143	**	0.002	HCW Wu3vacc
BA.5 vs. XBB.1	180	***	<0.001	HCW Wu3vacc
BA.5 vs. XBB.1.5	192	***	<0.001	HCW Wu3vacc
**ED Fig. 7b (binding)**	**Mean rank diff.**	**Significant?**	**Adjusted P Value**	**Cohort**
Wu-G614 vs. Wu-G614	-227	***	<0.001	KTR Wu4vacc / HCW Wu3vacc
Wu-G614 vs. Wu-G614	-254	***	<0.001	KTR Wu4vacc / HCW preOm+Wu3vacc
BA.1 vs. BA.1	-133	**	0.01	KTR Wu4vacc / HCW Wu3vacc
BA.1 vs. BA.1	-212	**	0.006	KTR Wu4vacc / HCW preOm+Wu3vacc
BA.5 vs. BA.5	-149	***	<0.001	KTR Wu4vacc / HCW Wu3vacc
BA.5 vs. BA.5	-231	***	<0.001	KTR Wu4vacc / HCW preOm+Wu3vacc
BA2.75.2 vs. BA2.75.2	-181	***	<0.001	KTR Wu4vacc / HCW Wu3vacc
BA2.75.2 vs. BA2.75.2	-249	***	<0.001	KTR Wu4vacc / HCW preOm+Wu3vacc
BQ.1.1 vs. BQ.1.1	-230	**	0.001	KTR Wu4vacc / HCW preOm+Wu3vacc
XBB.1 vs. XBB.1	-126	*	0.02	KTR Wu4vacc / HCW Wu3vacc
Wu-G614 vs. BA.1	140	**	0.002	HCW Wu3vacc
Wu-G614 vs. XBB.1	134	**	0.005	HCW Wu3vacc

*** (p-value < 0.001); ** (p < 0.002), * (p < 0.033) Kruskal-Wallis rank test and corrected with Dunn’s test
